# PLSKO: a robust knockoff generator to control false discovery rate in omics variable selection

**DOI:** 10.1093/bioinformatics/btaf475

**Published:** 2025-08-29

**Authors:** Guannan Yang, Ellen Menkhorst, Evdokia Dimitriadis, Kim-Anh Lê Cao

**Affiliations:** Melbourne Integrative Genomics, School of Mathematics and Statistics, The University of Melbourne, Parkville, VIC 3010, Australia; Department of Obstetrics and Gynaecology, The University of Melbourne, Parkville, VIC 3010, Australia; Gynaecology Research Centre, Royal Women’s Hospital, Parkville, VIC 3010, Australia; Department of Obstetrics and Gynaecology, The University of Melbourne, Parkville, VIC 3010, Australia; Gynaecology Research Centre, Royal Women’s Hospital, Parkville, VIC 3010, Australia; Melbourne Integrative Genomics, School of Mathematics and Statistics, The University of Melbourne, Parkville, VIC 3010, Australia

## Abstract

**Motivation:**

Integrating the knockoff framework with any variable-selection method delivers stringent false discovery rate (FDR) control without recourse to *p*-values, offering a powerful alternative for differential expression analysis of high-throughput omics datasets. However, existing knockoff generators rely on restrictive modelling assumptions or coarse approximations that often inflate the FDR when applied to real-world data.

**Results:**

We introduce Partial Least Squares Knockoff (PLSKO), an efficient, assumption-free generator that remains robust across diverse omics platforms. Our extensive simulations show that PLSKO is the only method to maintain FDR control with sufficient power in complex non-linear settings. Our semi-simulation studies drawn from RNA-seq, proteomics, metabolomics, and microbiome experiments confirm PLSKO generates valid knockoff variables. In pre-eclampsia multi-omics case studies, we combine PLSKO with Aggregation Knockoff to address the randomness of knockoffs and improve power, and demonstrate the method’s ability to recover biologically meaningful features.

**Availability and implementation:**

Our proposed algorithm is available on Github (https://github.com/guannan-yang/PLSKO) and Zenodo (https://doi.org/10.5281/zenodo.16879594)

## 1 Introduction

High-throughput technologies are widely used to measure system-wide biological variables, such as genes, proteins, metabolites, and bacteria. Analytical methods have been developed to extract useful information from these datasets. For example, differential expression (DE) analysis aims to identify biological variables whose expression or abundance levels vary between experimental groups; penalized regression aims to identify variables that are predictive of an outcome variable. These variables are called ‘discoveries’, and can then be used to generate hypotheses for further validation and investigation. It is therefore important to generate reliable discoveries that are not false positives.

False discovery rate (FDR) is a statistical criterion to control false positives in discoveries. Controlling FDR under a certain cut-off (e.g. 0.05) ensures the reliability of multiple testing in high-throughput data analysis. Most FDR control approaches, such as the Benjamini–Hochberg (BH) procedure, rely on a set of valid *p*-values (Benjamini and Hochberg 1995). However, the calculation of *p*-values requires strong distribution assumptions and is only achievable under a simple model or a simple estimation. For more complex data such as high-throughput data, valid *p*-values are difficult to calculate.

Barber and Candès (2015) introduced ‘knockoffs’, a framework for obtaining FDR control in feature selection while bypassing the standard calculation of *p*-values. The basic principle is to create artificial ‘knockoff’ variables that resemble the true correlation structure of the original data, but without using any information from the response variable. The original variables and their knockoff copy are run together into a model to measure the importance of each variable, so that the knockoff variables act as negative controls. A threshold is then derived to select the important variable set with controlled FDR.

The knockoff filtering procedure can be used with any feature selection method that generates importance measures under certain conditions (e.g. lasso penalized regression). The aim is to identify the true important variables that are directly related to response of interest, while excluding variables that are only indirectly related to the response through their correlation with those true important variables. However, the application of knockoff to high-throughput biological data remains limited. The procedure heavily depends on the construction of knockoff variables, which can be challenging in practice when the distributions of the explanatory variables are unknown. Biological data exhibit remarkable diversity, encompassing a wide range of data types derived from measuring various biological molecules or entities, and from various technological platforms. Even when considering the marginal distribution of a single biological variable, assumptions on the data distribution can be questionable (Li *et al.* 2022). A second-order knockoff procedure was introduced assuming variables follow a multivariate Gaussian distribution (Candès *et al.* 2018), but this can over-simplify the distribution of biological data. Furthermore, the joint distribution of biological variables is complex given their dependency structure (e.g. RNA sequencing, microbiome), thus invalidating the assumptions of second-order knockoffs.

Besides the distribution assumption of the knockoff procedures, another challenge is the high dimensionality of the data, where the number of variables far exceeds the number of samples. The ‘curse of dimensionality’ leads to either underfitting or overfitting modelling. For example, KnockoffScreen (He *et al.* 2021) can underfit on the conditional distribution when the number of samples is small, leading to inflated FDR as it fails to control on other variables. Another example is second-order approximation (SOA) knockoffs based on sample covariance, which is prone to overfitting and generates knockoff variables identical to the original ones, resulting in a lack of power (Candès *et al.* 2018). Therefore, knockoff variable generators need to be robust to different types of distributions, and be able to fit high-dimensional biological data efficiently.

We developed a new knockoff variable generator, Partial Least Squares Knockoff (PLSKO) as a first step of knockoff filtering. PLSKO is efficient, assumption-free and applicable to various types of data distribution, such as omics data. We demonstrate the robustness and improved performances in FDR control and statistical power compared to a wide range of existing knockoff generators in both simulation and semi-simulation studies. We apply PLSKO to multi-omics studies in pre-eclampsia, and compare our results to classical differential analysis. The selected features can then be used to identify biomarkers and for downstream analyses and modelling.

## 2 Materials and methods

### 2.1 Knockoff filtering: FDR control in variable selection

In the following, we denote *y* a clinical outcome or phenotype variable of interest with *n* observations, and X an (n×p) expression matrix where *p* is the number of omics variables (e.g. genes, proteins). $X_{j}$ represents a column from X for gene *j*. We first introduce knockoff filtering and then describe our proposed approach PLSKO.

#### 2.1.1 Controlled variable selection

Knockoff filtering is a procedure for controlled variable selection. Knockoff aims to select ‘relevant’ variables that are conditionally dependent on the response variable given all the other covariates, that is, a subset of X that directly affects *y*. $X_{j}$ is referred to as ‘null’ if it is conditionally independent of *y* given the other p−1 variables. As we consider the correlation among the variables into account, controlled variable selection ensures the hypotheses are mutually independent in multiple testing, thus avoiding ill-posed Type I error control and improving power. In practice, controlled variable selection improves the interpretability of the results and makes further investigation of causal relationships possible.

#### 2.1.2 False discovery rate

Given any variable selection procedure, a selected variable set is an estimate of the true important variable with random error. FDR is the expected false discovery proportion (FDP), that is, the proportion of null variables among all selected variables. FDP fluctuates around the FDR in any FDR-controlling procedure due to the randomness in both data and selection procedure. A knockoff filter selects variables controlling the FDR with finite sample guarantees (Barber and Candès 2015).

#### 2.1.3 Main steps of the knockoff filtering

**Figure 1.**
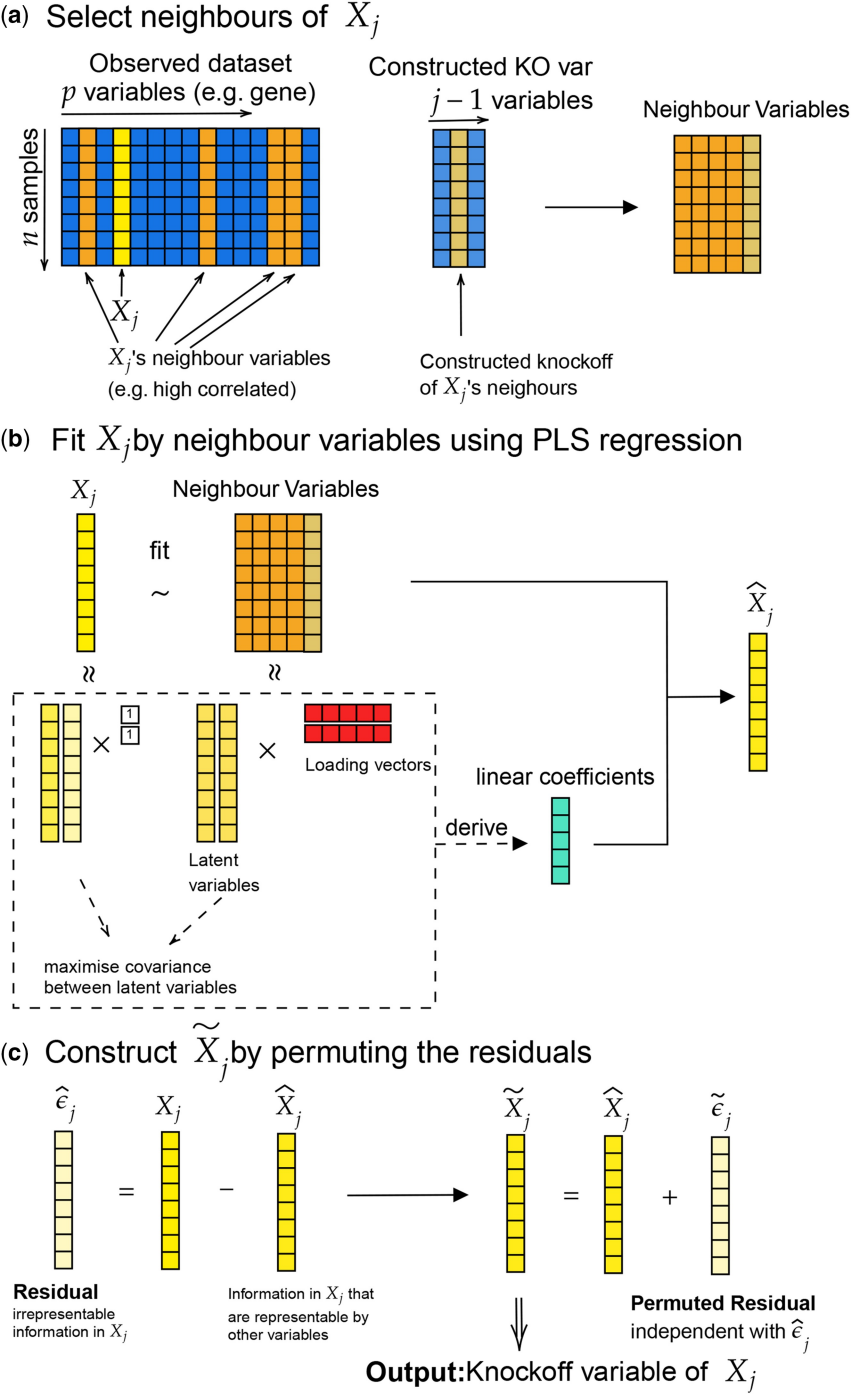
Steps of PLSKO to generate a knockoff variable ${\tilde{X}}_{j}$ of $X_{j}$. (a) We define neighbour variables that are either highly correlated with $X_{j}$, or based on a pre-defined list. (b) We then fit a PLS regression on $X_{j}$ using its neighbours and their knockoffs as predictors. (c) Finally, we calculate then permute the residuals to create the knockoff ${\tilde{X}}_{j}$. We iterate this process for each of the variables $X_{j}$, j=1,…,p.

Knockoff filtering consists of three main steps (summarized in Fig. 1, available as supplementary data at *Bioinformatics* online and described in detail in Section 3.1, available as supplementary data at *Bioinformatics* online):

**Figure 2.**
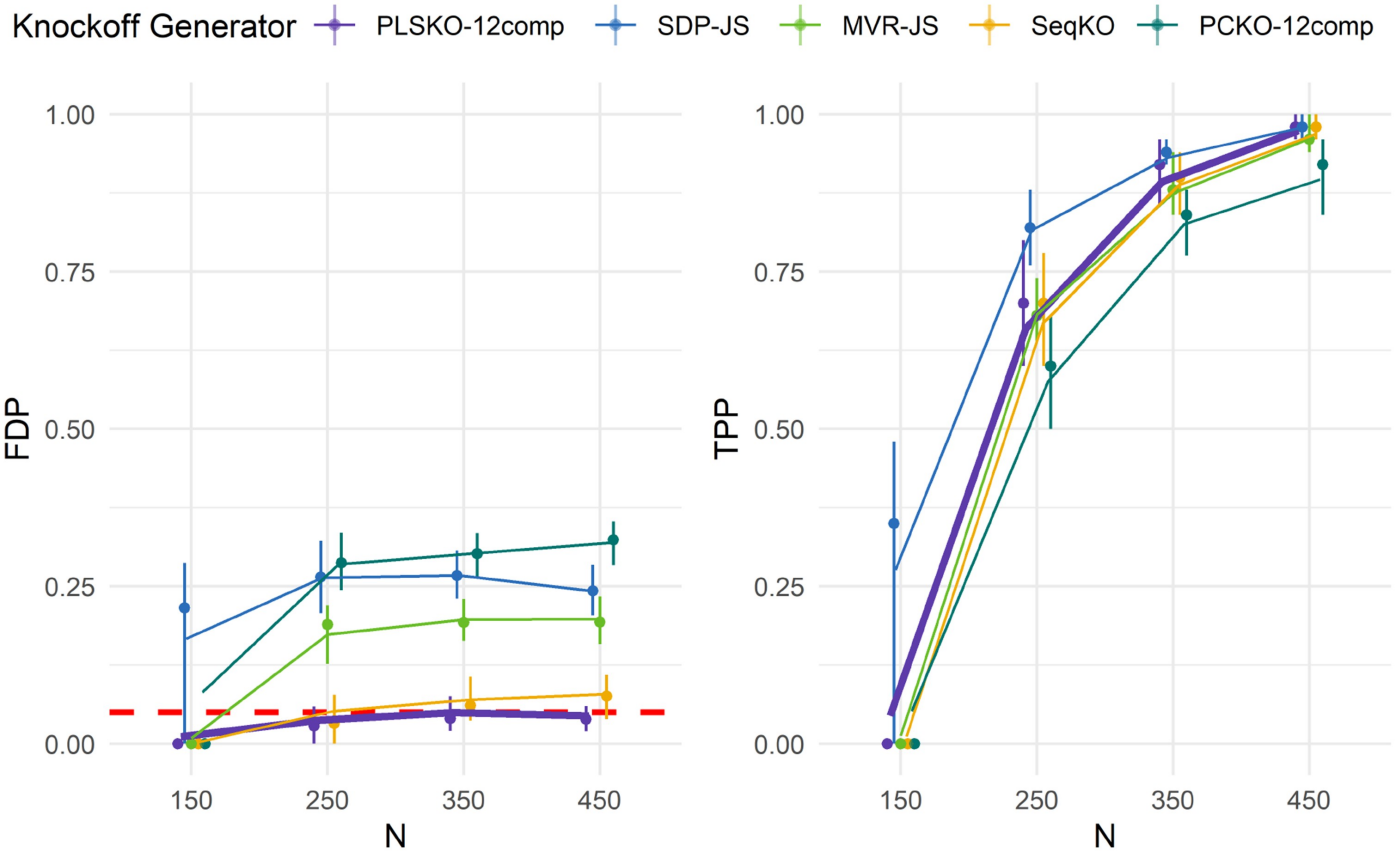
Simulations: FDR and power of PLSKO and other knockoff generators when X is generated from a quadratic factor model w.r.t. sample size, with p=500, five blocks, three latent factors, 10% signal proportion and a target FDR of 0.05 across 100 replications. Threshold $T_{+}$ is applied to control FDR. The lines represent the mean values across replicates, and the vertical bars indicate the interquartile range with the median shown as dots. Refer to Fig. 2, available as supplementary data at *Bioinformatics* online, for extended results.

- *Knockoff variable construction.* Knockoff variables, $\tilde{X}(n\times p)$, can be generated satisfying two properties of model-X knockoff for high-dimensional data where p>n (Candès *et al.* 2018): (i) the joint distribution of the original variables and the knockoff variables remain the same when swapping any subset of variables with their knockoff and (ii) the knockoff variables are constructed regardless of the information from *y*. As a ‘negative control’, a knockoff variable contains all the information from its original variable which can be reconstructed by the other variables. We describe existing approaches to construct model-X knockoff in Method 3.2, available as supplementary data at *Bioinformatics* online.
- *Important statistics calculation.* A variable selection model (e.g. lasso penalized regression) is then run on *both* original and generated knockoff variables as covariates, with the response variable *y*. Importance scores, $W_{j},j=1,\ldots ,p$, can be obtained for $X_{j}$ as the ‘true’ importance score of the variable after removing the ‘negative control’ part. A valid *W* of null variables should be distributed symmetrically to zero (Barber and Candès 2015). A large positive value of $W_{j}$ provides some evidence that the distribution of *y* depends upon $X_{j}$, whereas small positive or negative values provide evidence of null variables. For example, an option to calculate $W_{j}$ is the lasso coefficient difference (LCD) between $X_{j}$ and its knockoff ${\tilde{X}}_{j}$, i.e. $W_{j}=|\text{coef}(X_{j})|-|\text{coef}({\tilde{X}}_{j})|$.
- *Threshold identification and variable selection.* To control FDR at a pre-specified level *q* (e.g. 0.05), we determine the threshold *T* as the minimum value that allows the proportion of variables with negative W≤−T compared to the variables with positive W≥T lower (or equal) than *q*. With a well-defined *W*, the number of null variables in the selected variable set can be estimated by the number of variables with large negative *W* which is expected to be equal to the number of null variables (i.e. false discoveries with large positive *W*). We select variables with $W_{j}\geq T$ with modified FDR controlled. Alternatively, we can increment the number of negatives in discoveries by 1 to identify the threshold $T_{+}$ to control FDR in a slightly conservative manner (both options are detailed in Method 3.1, available as supplementary data at *Bioinformatics* online).

#### 2.1.4 Sequential Conditional Independent Pairs

Sequential Conditional Independent Pairs (SCIP) has been proposed as a general algorithm for exact knockoff variable construction. It does not require distribution assumptions on X (Candès *et al.* 2018). SCIP forms the basis of our proposed approach. The algorithm (Algorithm 1, available as supplementary data at *Bioinformatics* online) is based on the pairwise exchangeability property of model-X knockoffs, that is, a pair of a variable and its knockoff is distributionally non-distinguishable conditioning on all other variables and their knockoffs. To reduce computational costs, we can generate ${\tilde{X}}_{j}$ by sampling from the conditional distribution of $X_{j}$ given the neighbours of $X_{j}$ and ${\tilde{X}}_{j}$, with the assumption of conditional independence to non-neighbours (He *et al.* 2021).

### 2.2 Knockoff variable construction for biological data

Our new method ‘PLSKO’ incorporates partial least squares regression (PLS, Wold 1966) into the SCIP algorithm, a valid model-X knockoff generation algorithm (Candès *et al.* 2018). PLSKO addresses the limitations of existing knockoff generators for biological data, which tend to either under- or over-fit due to high dimensionality and unspecific distribution.

#### 2.2.1 PLS regression

PLS encompass a wide class of techniques for modelling relations between sets of variables. The assumption of PLS is that predictors and the response are driven by a small number of latent variables. PLS have been widely applied to high-dimensional biological data due to its high computational and statistical efficiency when p>n (Boulesteix and Strimmer 2007). In our proposed PLSKO, we use PLS regression to calculate the conditional distribution in a sequential manner, with the aim of effectively controlling the information explainable by other variables. Details can be found in Method 3.3.4, available as supplementary data at *Bioinformatics* online.

#### 2.2.2 Main steps of PLSKO

The first step of PLSKO is optional (Fig. 1). Given a set of biological variables X, we can define neighbour variables. Neighbour variables refer to other variables that are assumed to capture most of the dependency or predictive information about $X_{j}$ and are used to conditionally model its distribution. This step aims to reduce the number of variables being controlled in the conditional distribution, thus improving computational efficiency. We assume that a variable is conditionally independent of its non-neighbours given its neighbours. This assumption allows us to estimate the conditional distribution of $X_{j}$ by only controlling its neighbours, which is theoretically equivalent to controlling on all variables. Neighbour variables can be determined using different approaches. By default, our method defines neighbours as variables highly correlated with $X_{j}$. Alternatively, neighbours can be predefined, such as network-inferred relationships or leveraging prior biological knowledge.

PLSKO generates knockoff variables from the first variable in the dataset to the last, individually, as in the SCIP algorithm. For each variable $X_{j}$, j=1…p, PLSKO calculates the conditional distribution of $X_{j}$ (given its neighbours and their knockoff variables) by fitting a PLS regression where $X_{j}$ is the response and the covariates are neighbour variables and neighbours’ constructed knockoff variables. By sampling from the estimated conditional distribution—adding the fitted values to permuted residuals—we generate the knockoff variable $\tilde{X_{j}}$. This permutation-based approach is non-parametric and ensures PLSKO can be robustly applied to different types of data with unspecified distribution while controlling FDR. From PLSKO, we obtain the knockoff variable dataset $\tilde{X_{j}}$  (n×p) as the output of the first step of knockoff filtering. We can then proceed with the subsequent two steps of the knockoffs described above to select important variables. Here, we used the LCD from lasso as the importance statistics in this paper, but since PLSKO is a knockoff generator, it can be combined with other model, such as Bayesian variable selection, without affecting FDR control. However, the choice of model may influence power. Full methodological details are available in Methods 3.3 and Algorithm 2, available as supplementary data at *Bioinformatics* online.

#### 2.2.3 Parameter specification of PLSKO

There are three types of parameters to consider in PLSKO:

- *Neighbour list.* In our default setting, we define neighbours based on sample correlations. The threshold value can be pre-specified, or based on a quantile of all correlations, depending on how sparse the co-expression network is assumed to be. Generally, a lower threshold increases knockoff similarity to the original variables, which can lead to overfitting, smaller Type I error but also lower power.
- *Number of latent components in PLS.* The larger the number of latent components, the more similar the knockoff to the original variables, which can lead to a lower Type I error but also lower power.
- *Variable kept in sparse PLS.* We provide the option of sparse PLS, a lasso penalized variant designed for variable selection (sPLS, Lê Cao *et al.* 2008). This version improves the conditional distribution calculation. The percentage of variables kept on each component is user-defined.

The robustness tests of PLSKO (Figs 7–10, available as supplementary data at *Bioinformatics* online) and the semi-simulation on real data (Figs 18–21, available as supplementary data at *Bioinformatics* online) suggest an appropriate neighbour threshold is crucial to ensure the FDR control, while the number of components has less impact but may still affect the performance in both FDR control and power. We provide a function to tune the parameters and ensure FDR control (see Method 3.3.1, available as supplementary data at *Bioinformatics* online).

#### 2.2.4 Partial least squares–Aggregation of Multiple Knockoffs

In our case studies, we used the Aggregation of Multiple Knockoffs procedure to improve stability and reduce randomness in knockoff generation (AKO, Nguyen *et al.* 2020). We refer to this method as ‘PLS-AKO’. We generated multiple knockoff datasets using PLSKO, then calculated LCD as the importance score *W*, based on which we computed an empirical *p*-value for each $X_{j}$ in each run. These empirical *p*-values from multiple runs were aggregated using a quantile-based method to obtain a single aggregated *p*-value for each $X_{j}$. The FDR is then controlled by applying the BH procedure to the aggregated *p*-values, selecting variables accordingly. Aggregating multiple knockoff runs generally resulted in a set of selected variables with lower FDR, higher power, and higher stability compared to the average of single-run knockoffs. Details can be found in Methods 3.3.3 and 3.9, available as supplementary data at *Bioinformatics* online (comparisons to the multiple knockoff by Gimenez and Zou 2019 and Nguyen *et al.* 2020).

### 2.3 Benchmark methods

We performed a series of simulation experiments with varying settings to compare the performance of knockoff generators, including SOA methods, SCIP methods, and PLSKO.

SOA methods benchmarked in this study included semi-definite program (SDP) knockoff (Candès *et al.* 2018) and minimized reconstructability knockoff with minimum variance-based reconstructability loss (MVR, Spector and Janson 2022). Since this type of method requires mean and covariance of X to be known or estimated, our benchmark considered ground truth covariance when available (namely ‘oracle’) or James–Stein (JS) style shrunk covariance (Ledoit and Wolf 2003), the latter used at default method in the knockoff R package for high-dimensional data. SCIP-based knockoff generators include SeqKnockoff (seqko) with lasso regression for conditional distribution approximation (Kormaksson *et al.* 2021) and KOBT with PC regression (PCKO, Jiang et al. 2021). We also evaluated a knockoff generator for data following factor model, namely ‘Intertwined probabilistic factors decoupling’ (Fan *et al.* 2020). These methods are detailed in Methods 3.4 and 3.5, available as supplementary data at *Bioinformatics* online.

In our benchmark, we examined the effect of various neighbourhood thresholds and the number of components in PLSKO, denoted as:

- PLSKO: Arbitrary and liberal setting. Neighbour threshold of 80% quantiles, meaning the top 20% of the most correlated pairs are used for PLS fitting, and five components.
- PLSKO-full: Most conservative setting. No neighbour filtering and all other variables are used for fitting. In simulations the number of factors is set to be the known true rank; for real data, the number of factors of PLSKO is chosen by the $PC_{p1}$ criterion (Bai and Ng 2002).
- PLSKO-full-sparse: Parameters are the same as PLSKO-full but using sPLS regression with 20% of variables kept.

### 2.4 Pre-eclampsia studies

Pre-eclampsia is a life-threatening disease of pregnancy and a leading cause of maternal and neonatal morbidity and mortality, diagnosed by sudden-onset hypertension (>20 weeks of gestation) and other maternal organ or placental dysfunction (Dimitriadis *et al.* 2023). We analysed two case–control studies of pre-eclampsia for both our semi-simulations and our case studies. The first study contains maternal circulating cell-free RNA-seq (cfRNA) data from 71 samples (16 pre-eclamptic, 55 normotensive) from Moufarrej *et al.* (2022). The second study is a multi-omics study that includes proteomics, metabolomics, and microbiome data with either 36 samples (18 pre-eclampsia, 18 normotensive) or 49 samples (microbiome data only, 29 preeclamptic, 20 normotensive), from Marić *et al.* (2022). To avoid repeated measurements and maintain the assumption of *i.i.d.* samples in knockoff filtering, we only retained the first sample of each individual (before 12 weeks of gestation).

## 3 Results

### 3.1 Simulation results

Our simulations aimed to answer the following: (i) Does PLSKO control FDR with high power? and (ii) How robust are knockoff generators when their assumptions are violated?

####  

**Figure 3.**
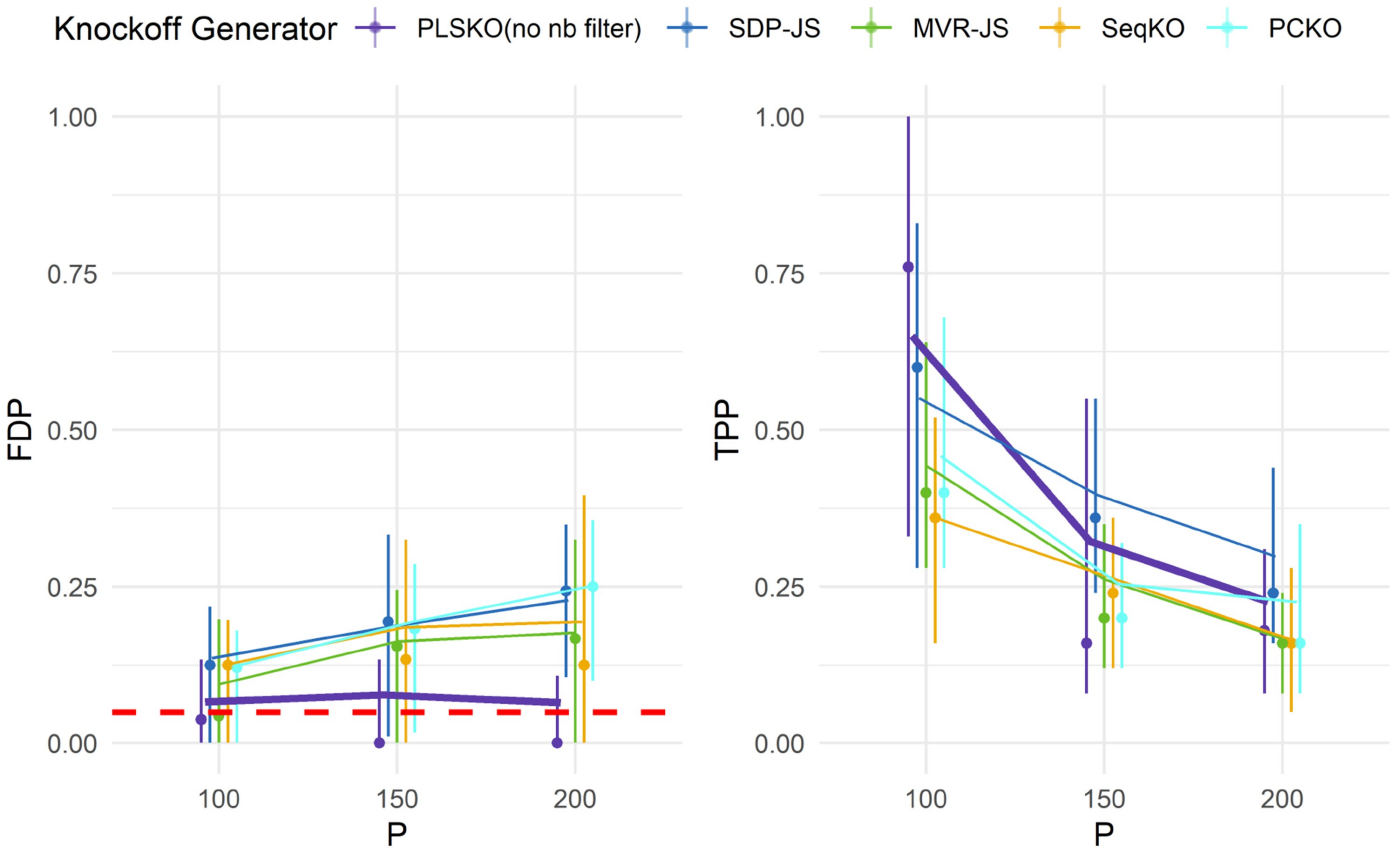
Semi-simulations: FDR and power of PLSKO and other knockoff generators on cell-free RNA-seq data w.r.t. numbers of variables, with target FDR = 0.05, sample size n=71, number of important variables $p_{s}=25$, over 50 repetitions. Threshold *T* is applied to control modified FDR. The lines represent the mean values across replicates, and the vertical bars indicate the interquartile range with the median shown as dots. See Fig. 11a, available as supplementary data at *Bioinformatics* online, for extended results.

Most knockoff generators have been shown to control FDR in simplistic simulations, where X follow a multivariate Gaussian distribution. We have confirmed these results, and also showed that the FDR was controlled when X is generated from a Gaussian factor model (Figs 3–6, available as supplementary data at *Bioinformatics* online). In these scenarios, the relationships between variables are linear, which may not capture the complexity of real biological data. To test whether PLSKO and other methods can robustly handle more challenging cases where the relationships between variables are non-linear, we simulated data from a quadratic factor model: half of the variables were generated from a block factor model and the other half as their square value. We randomly selected a subset of variables as important variables to generate a response *y* as a linear combination of these variables. For each given parameter configuration, we simulated 100 high-dimensional datasets, with p=500. We then generated knockoff variables with different generators, and used the LCD as the importance statistics *W* and the threshold $T_{+}$ in knockoff filtering at target FDR q=0.05. The FDP and the true positive proportion (TPP) of the selected variable set were used to assess the validity and performance of the knockoff generators. The average FDP gives an estimate of FDR, whereas the average TPP gives an estimate of power (defined in Method 3.5.3, available as supplementary data at *Bioinformatics* online). Simulations are detailed in Method 3.5 and Table 8, available as supplementary data at *Bioinformatics* online.

####  

PLSKO with 12 components was the only method that empirically controlled the FDR for any sample size, while PLSKO with nine components and SeqKnockoff had relatively low FDR<0.10 (Fig. 2; Fig. 2, available as supplementary data at *Bioinformatics* online). Other methods had a large FDR. Especially, the SOA method SDP with JS covariance and PCKO had an FDR>0.15, even for a number of components in the principal component regression similar to those in PLSKO (9 and 12). Due to its iterative nature, the PLS regression results in a better fit in controlling other variables with a non-linear relationship compared to principal component regression in PCKO. PLSKO was highly efficient as it utilizes a relatively small number of components, and its power of PLSKO did not reduce when we increased the number of components.

### 3.2 Semi-simulation results

The assumption that biological data follow a Gaussian or factor model might not be realistic. We showed in our simulation results that violation of assumption from knockoff generators, or poor fitness can lead to inflated FDR. Thus, we next used real biological data X, but we simulated *y*, and checked the validity and robustness of knockoff generators, including PLSKO, on different types biological data with unknown distribution.

####  

We performed semi-simulations based on the two pre-eclampsia studies. We ran the semi-simulation for each dataset over 50 repetitions. In each run, we first randomly selected a subset of variables $X^{\mathrm{subset}}$ to avoid zero power in high-dimensional data, and to retain the joint distribution of the original X. Similar to our simulations, we simulated *y* from a set of randomly selected important variables from $X^{\mathrm{subset}}$. After generating knockoffs for $X^{\mathrm{subset}}$, we applied knockoff filtering with the threshold *T* a target level of FDR (cfRNA: 0.05, multi-omic datasets: 0.10); and used FDP and TPP as a measure of performances from each repetition.

####  

For datasets from both cfRNA and multi-omics studies, we found that both PLSKO-full and PLSKO-full-sparse were the only methods controlling for the modified FDR close to the controlled level, regardless of the number of variables in $X^{\mathrm{subset}}$ (cfRNA: Fig. 3; Fig. 11, available as supplementary data at *Bioinformatics* online, multi-omics: Fig. 13, available as supplementary data at *Bioinformatics* online). The other methods, including PLSKO with arbitrary and liberal parameters (three components and 80% top correlation) had inflated FDR around 0.2. We determined the number of components in PLSKO-full and PLSKO-full-sparse based on the $PC_{p1}$ criterion from Bai and Ng (2002), ranging from 4 to 8 with a mode of 6 in the repeatedly sampled $X^{\mathrm{subset}}$. Despite the inherent trade-off between FDR and power, both methods were able to generate knockoff variables with the lowest FDR and modestly enhanced power. Additionally, PLSKO-full-sparse performed similarly to PLSKO-full but only included 20% of variables on each component for fitting the conditional distribution. This result suggests that sparse PLS regression did not cause under-fitting. Since RNA-seq counts are assumed to marginally follow a negative binomial distribution (Robinson and Smyth 2008), which violates the assumption in SOA methods, we further normalized the RNA-seq data to ensure that $X^{\mathrm{subset}}$ follow a Gaussian distribution. However, we still found that the FDR was inflated and power was low (Fig. 11b, available as supplementary data at *Bioinformatics* online), suggesting poor fitness of covariance and information loss.

In summary, both PLSKO-full and PLSKO-full-sparse with no prior neighbour screening and a number of components determined by the $PC_{p1}$ criterion were robust to different types of real biological data without over-simplifying the assumptions on X. We found that the other knockoff generators and PLSKO with arbitrary liberal setting failed due to insufficient fitness on these complex data.

### 3.3 Case studies

We then applied PLSKO on the pre-eclampsia cfRNA and multi-omics studies, where the response variable is preeclampsia or not. We first reduced the number of variables in the datasets by focusing solely on circulating placenta-elevated genes and proteins. As abnormal placenta is central to preeclampsia, our hypothesis was that this subset of genes and proteins might reflect changes from placenta. In the cfRNA dataset, we selected 81 placenta-elevated genes defined according to The Human Protein Atlas (Uhlén *et al.* 2015). In the proteomics dataset, we selected 36 placenta-released proteins from plasma according to Degnes *et al.* (2022). For the microbiome data, we kept 144 Operational Taxonomic Units (OTUs) filtered with a sum of counts >0.01% of the total sum of counts.

We generated knockoff variable sets for each dataset using PLSKO. Semi-simulations on the real data were performed to tune the parameters of PLSKO to ensure that the knockoff variables controlled the FDR empirically (Figs 18, 20, and 21, available as supplementary data at *Bioinformatics* online). The LCD generated from logistic lasso regression was used as importance statistic. We did not include any covariate as this information was not publicly available. We discussed, however, how to include covariates in Section 3.8.3, available as supplementary data at *Bioinformatics* online. To accommodate for the randomness of knockoff generators, we ran PLSKO 50 times and aggregated the result using PLS-AKO with target FDR level 0.05. We compared our selection with two commonly used methods for DE, limma (Smyth 2005) and Wilcoxon test with BH procedure (Table 1).

**Table 1.** Genes, proteins from plasma, and OTUs (reported either at the species ‘s_’, or genus ‘g_’ levels) identified as frequently selected by PLSKO run 50 times (frequency >0.1), and results aggregated with PLS-AKO.

Dataset	Predictors	Selected feature	PLSKO frequency	PLS-AKO selection	Declared DE
cell-free transcriptomics (*n* = 71)	Placenta-specific elevated gene expression (*P* = 81)	TENT5A/FAM46A	0.88	Yes	Yes^l,w^
		PHACTR2	0.84	Yes	No
		MBNL3	0.64	Yes	Yes${}^{w}$
		GSE1	0.62	Yes	No
		HEMGN	0.46	Yes	No
		MAFK	0.38	Yes	No
		BPGM	0.36	Yes	Yes${}^{w}$
		CSF2RB	0.22	No	No
		COBLL1	0.14	No	No
		TRIM10	0.12	No	No
Proteomics from multi-omics study (*n* = 36)	Placenta-release protein (*P* = 36)	HSPB1	0.4	Yes	No
		CXCL10	0.32	Yes	No
		DKK1	0.2	No	No
		SERPINE1	0.2	No	No
Microbiome data (*N* = 49)	144 OTUs	g_*Corynebacterium*	0.3	Yes	No
		s_*F. nucleatum*	0.3	Yes	No

Selections are compared with differential expression (DE) tests limma (^l^) and Wilcoxon tests (^w^).

In the cfRNA data, 11 genes were selected more than 10% of the time across the 50 repetitions by PLSKO. Amongst those genes, seven were selected by PLS-AKO, whereas limma selected one and the Wilcoxon test three of these genes. Some of the genes only selected by PLSKO are likely to be biologically meaningful in the context of pre-eclampsia. For example, MAFK serves as a critical partner of NRF2 in regulating antioxidant responses, a pathway that is activated in pre-eclampsia due to oxidative stress and inflammation (Tossetta *et al.* 2023). The second most frequently selected variable, *PHACTR2*, was identified only by PLSKO and was not significantly related to pre-eclampsia in a marginal test (see Table 4, available as supplementary data at *Bioinformatics* online) but became significant when controlling for *TENT5A* using logistic regression with Wald test (see Table 5, available as supplementary data at *Bioinformatics* online). This suggests that *PHACTR2*’s effect was masked in a marginal test but became detectable when accounting for confounding factors. Three highly correlated variables, *MBNL3*, *HEMGN*, and *BPGM* were identified by PLSKO. We then performed downstream logistic regressions to illustrate the differences between variable selection methods. We found that when all seven variables were fitted in the regression model, only one remained significant. However, removing any two (either *MBNL3*, *HEMGN*, and/or *BPGM*) made the others five variables significant (see Table 6, available as supplementary data at *Bioinformatics* online). These results illustrate how PLSKO, as a controlled variable selection method, can address collinearity and account for potential confounders, ensuring that important variables can be identified even when they are correlated with others.

In each of the multi-omic studies, PLSKO and PLS-AKO selected several biological variables missed by the DE tests. All proteins selected by PLSKO have been reported to be related to preeclampsia, including SERPINE1 (Zhao *et al.* 2013), HSPB1 (Martin *et al.* 2022), CXCL10 (Gotsch *et al.* 2007), DKK1 (Kasoha *et al.* 2021). Similarly, the selected OTUs included the species *Fusobacterium. nucleatum* (Barak *et al.* 2007) and the genus *Corynebacterium* (Pax *et al.* 2024). Results with various types of prefiltering are presented in Tables 1 and 2, available as supplementary data at *Bioinformatics* online.

The selection frequency from multiple runs of PLSKO serves as a proxy for a variable’s predictive power, reflecting its stability under the knockoff filtering procedure. Some selected variables presented low selection frequency, which may result from limited statistical power or false discoveries selected in only a few runs. Low power can stem from high correlations between variables, small sample sizes, noise in the response variables, or a suboptimal variable selection model. To enhance power and FDR control, we recommend aggregating multiple runs using PLS-AKO, ideally with at least 25 runs.

Overall, our application of PLSKO in biological datasets showed that our method is able to identify biologically relevant omics features with higher power than marginal DE tests.

## 4 Discussion

PLSKO is a new knockoff variable generator for biological high-throughput data. It is a filtering approach leading to the selection of important variables related to a response of interest with FDR control. PLSKO can be paired with various variable selection models depending on the type of analysis and biological questions. Our results in simulations, semi-simulations and applications in multiple types of omics data (RNA-seq, proteomics, microbiome) show that PLSKO is more robust in FDR control and has higher statistical power than existing knockoff generators. Compared to classic DE analysis, we also showed that PLSKO selected more variables that were biologically highly relevant.

In our case studies, we filtered subsets of variables before applying PLSKO. Despite the theoretical guarantees for FDR control, practical limitations—such as zero power and empirical FDR inflation—were observed when our method was applied to data with p>3n at targeted FDR level of 0.05 (Figs 11 and 12, available as supplementary data at *Bioinformatics* online). To ensure effective FDR control, we therefore recommend reducing the number of variables before applying PLSKO. The choice of variable reduction approach should align with the biological question of interest and can include methods such as meta-gene aggregation, most variable genes, or knowledge-driven strategies.

Our method may select different sets of variables compared to other DE methods, such as limma and Wilcoxon test. This discrepancy arises from fundamental differences in hypothesis testing: DE methods assess marginal associations, whereas PLSKO evaluates conditional associations (see Section 2.1.1). Marginal tests select both truly important variables and those correlated with them without distinction. Moreover, when the assumption on the relationship between *X* and *y* is violated, DE methods may fail to control the FDR (see Figs 14–17, available as supplementary data at *Bioinformatics* online). In contrast, PLSKO maintains FDR control regardless of the assumption. However, another potential factor contributing to differences in selection is the lower power of PLSKO in extremely high-dimensional settings, which may lead to missing some important variables. Future extensions of PLSKO can address the issues of low power in extreme high-dimensional settings.

PLSKO has demonstrated robustness across different types of omics data, when being applied to single omics. This suggests its potential for methodological extension to multi-omics studies to ensure statistical validity. Future work should focus on enabling broader applications in integrative omics research, enhancing biological insights, and improving reproducibility in complex disease studies.

## Supplementary Material

btaf475_Supplementary_Data

## Data Availability

All analyses were conducted in R. Code and vignette are available in Github (https://github.com/guannan-yang/PLSKO) and Zenodo (https://doi.org/10.5281/zenodo.16879594). The R packages used are listed in Method 3.10, available as supplementary data at *Bioinformatics* online.
