# Supplementary material for: PLSKO: a robust knockoff generator to control false discovery rate in omics variable selection
Source: Bioinformatics. 2025 Aug 29;41(9):btaf475. doi: 10.1093/bioinformatics/btaf475 (PMC12449248; doi:10.1093/bioinformatics/btaf475)
Supplement: btaf475_Supplementary_Data [file btaf475_supplementary_data.zip › PLSKO_minorRevision1_Templated (1).pdf]

# Supplementary Material of ‘PLSKO: a robust knockoff generator to control false discovery rate in omics variable selection’

Guannan Yang[1], Ellen Menkhorst[2,3], Evdokia Dimitriadis[2,3], and Kim-Anh Lê Cao[1,*]

[1]Melbourne Integrative Genomics, School of Mathematics and Statistics, The University of Melbourne, Parkville, Victoria, Australia
[2]Department of Obstetrics and Gynaecology, School of Mathematics and Statistics, The University of Melbourne, Parkville, Victoria, Australia
[3]Gynaecology Research Centre, School of Mathematics and Statistics, The University of Melbourne, Parkville, Victoria, Australia
[*]Corresponding author. kimanh.lecao@unimelb.edu.au

# S1 Supplementary Figures

## S1.1 Method diagram

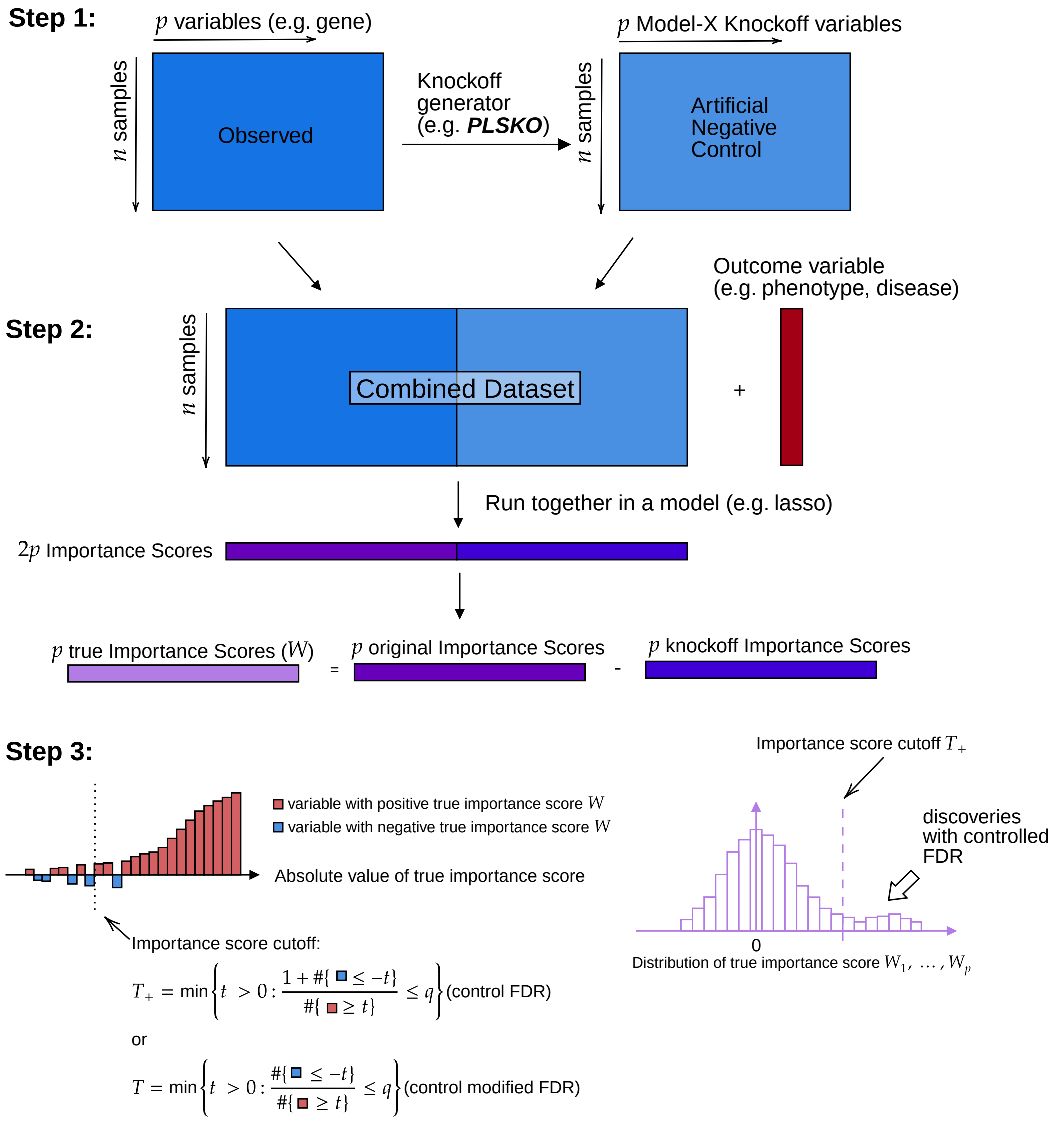


Figure S1: Overview of knockoff filtering. Step 1: Knockoff variable construction: we generate knockoff variables that follow model-X properties as the artificial negative control. Step 2: we run the original and knockoff variables together to calculate importance score that measures the *true* importance of each variable to the response variable $y$ by contrasting it with its knockoff. For example, the lasso coefficient difference (LCD) can be used, but other models and importance statistics that follow the *flip-sign* property are also applicable. Step 3:we determine a threshold to control the proportion of large negative importance scores over the large positive scores that is less than the nominal FDR level and select the variables with larger importance scores.

## S1.2 Simulation results

### S1.2.1 Benchmark to existing knockoff generators

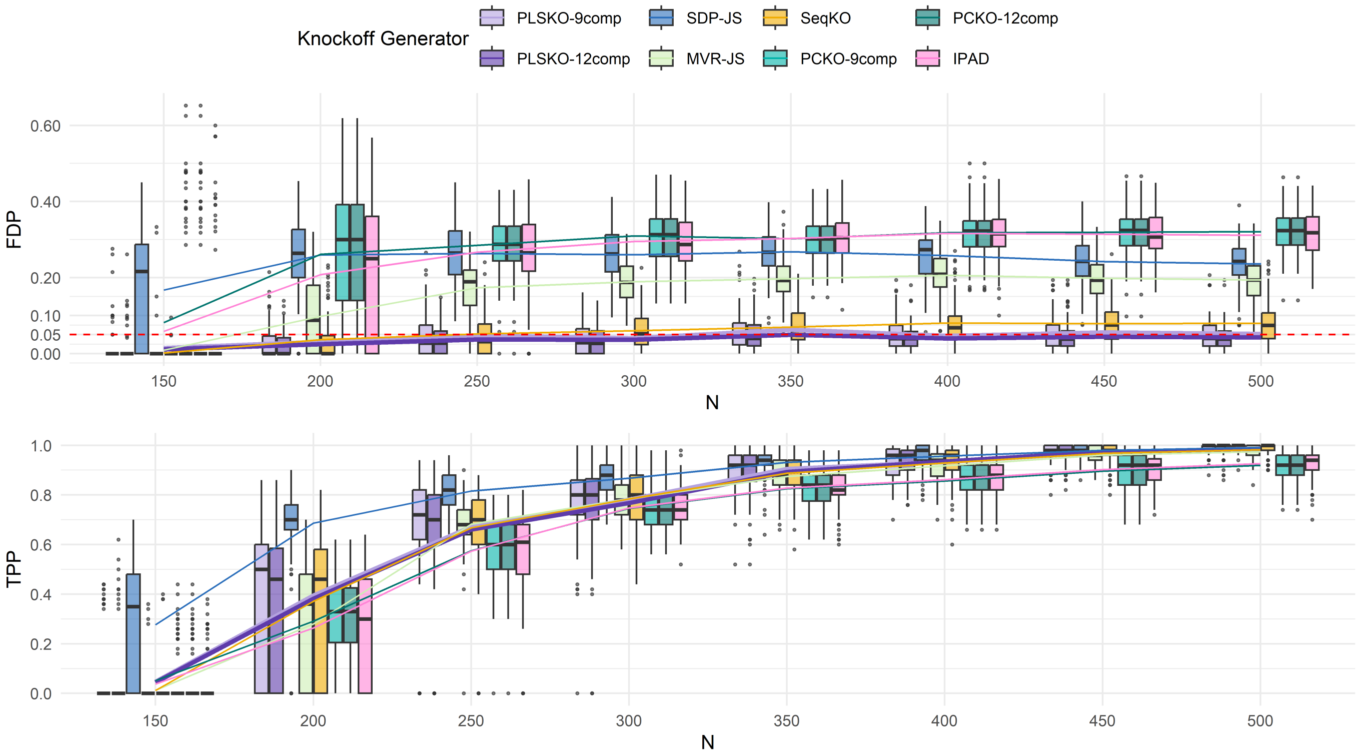


Figure S2: Expanded result of Figure 2. Simulations showing the FDR and power of PLSKO and other knockoff generators when $X$ is generated from a quadratic factor model w.r.t sample size, with $p = 500$, 5 blocks, 3 latent factors, 10% signal proportion and a target FDR of 0.05 across 100 replications. Threshold $T_+$ is applied to control FDR. FDP: false discovery proportion; TPP: true positive proportion. Solid lines represent the mean of FDP and TPP, i.e., the estimates of FDR and power.

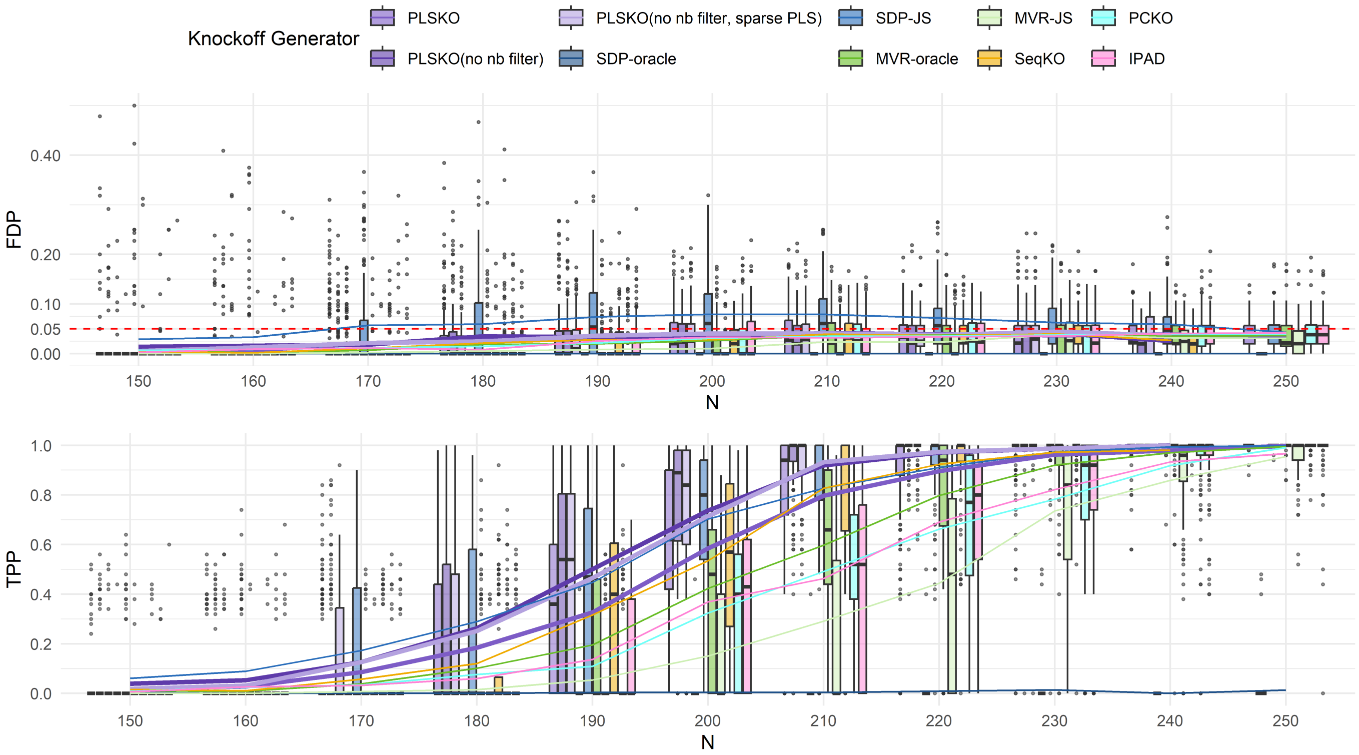


Figure S3: Simulation results: FDR and power of PLSKO and other knockoff generators when $\mathbf{X}$ follows a Gaussian factor model w.r.t sample size, with $p = 500$, 5 blocks, 3 latent factors, 10% signal proportion and a target FDR of 0.05 across 100 replications. Threshold $T_+$ is applied to control FDR. FDP: false discovery proportion; TPP: true positive proportion. Solid lines represent the mean of FDP and TPP, i.e., the estimates FDR and power.

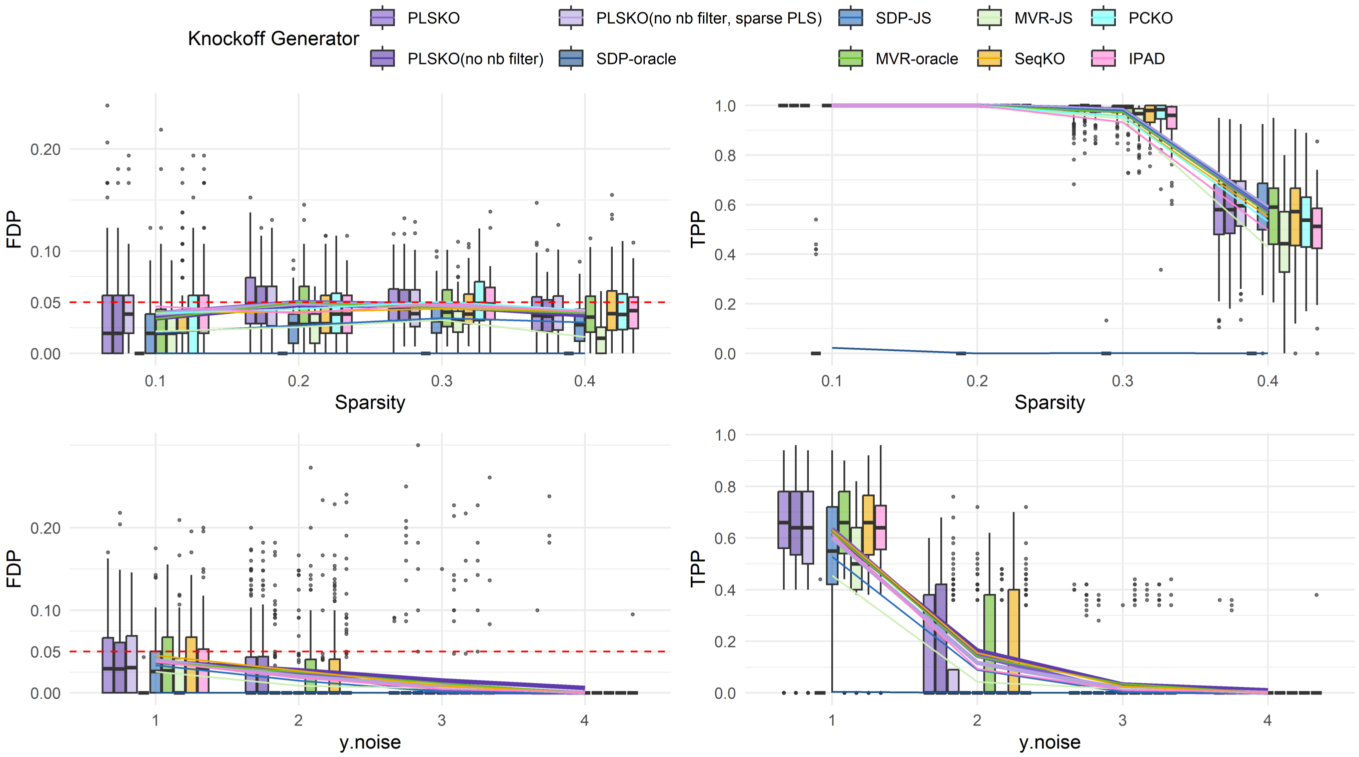


Figure S4: Simulation on block factor model with $y$ generated when $\mathbf{X}$ follows a Gaussian factor model w.r.t (a) varying proportions of important variables $p_s$ and (b) varying noise added to $y$, with $n = 500, p = 500$, 5 blocks, 3 latent factors, 10% signal proportion and a target FDR of 0.05 across 100 replications. Sparsity represents the proportion of important variables, that is, 0.1 represents 10% of $p$ variables used to generate $y$, and so on. y.noise ($c$): a random noise $\epsilon \sim \mathcal{N}(0, p_s \times c^2)$ is added to $y$, representing the ratio of noise to signal, that is when $c = 1$ the noise signal ratio is 16:1. Threshold $T_+$ is applied to control FDR.

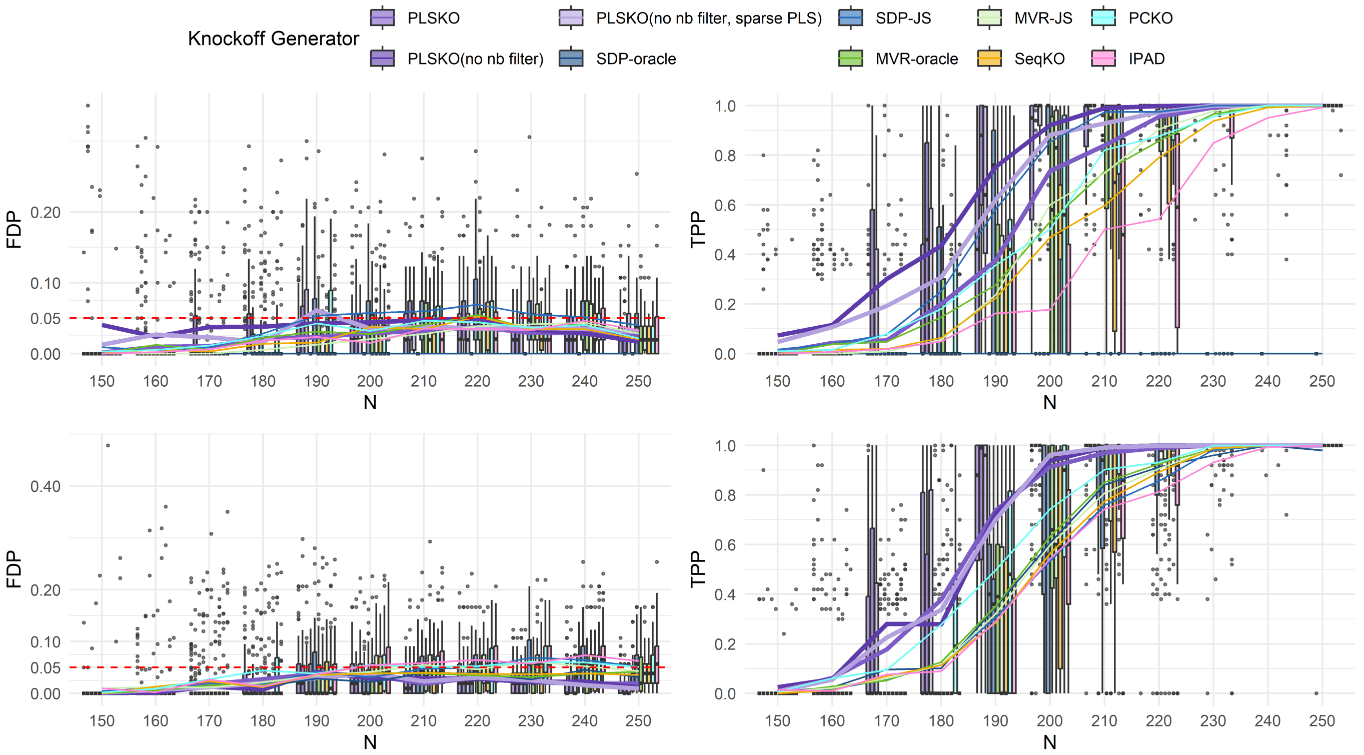


Figure S5: Simulation experiments on multivariate Gaussian distribution with (a) block equi-correlated covariance ($\rho = 0.5$) and (b) AR1 covariance ($\rho = 0.5$), across over varying sample sizes, with $p = 500$, 5 blocks, 3 latent factors, 10% signal proportion and a target FDR of 0.05 across 100 replications.

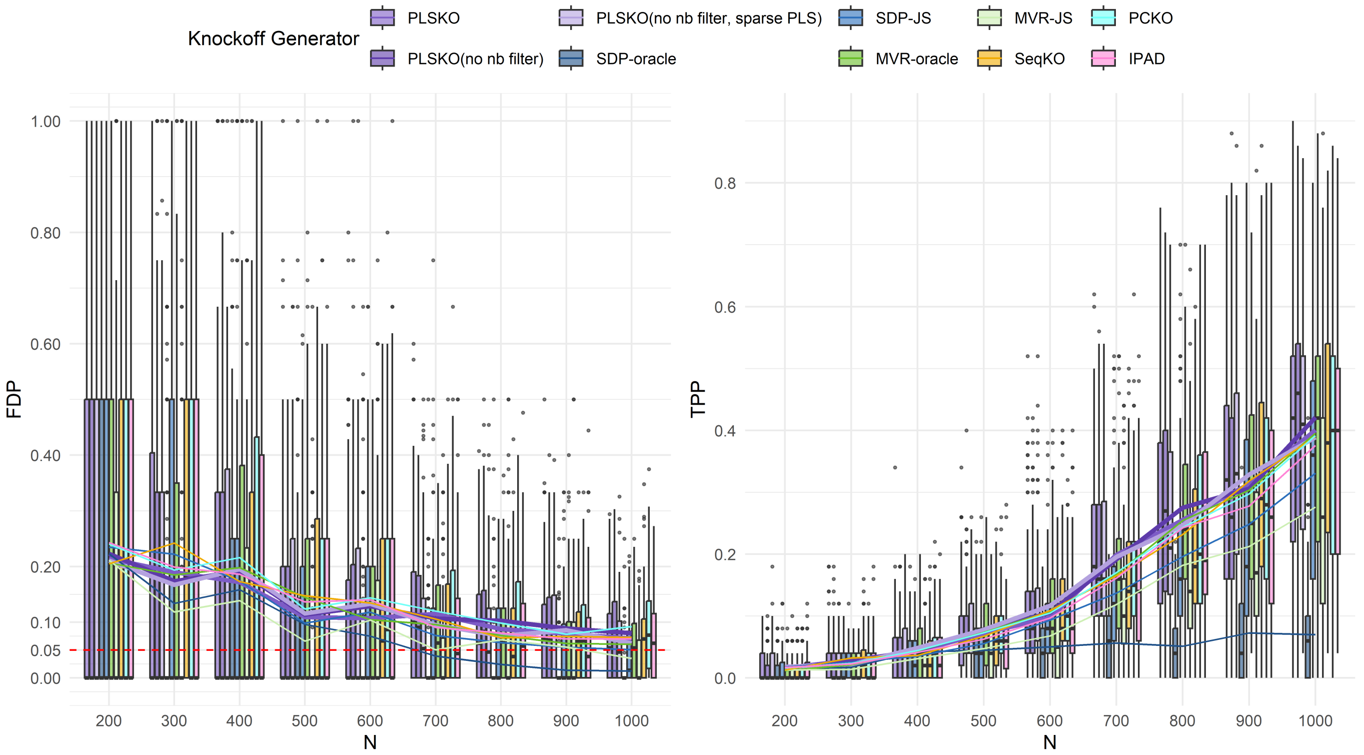


Figure S6: Simulation with varying sample size with categorical $y$ (controlled on modified FDR) when $X$ follows Gaussian factor model with $p = 500$, 5 blocks, 3 latent factors, 10% signal proportion, $y$ generated by Sigmoid function of the linear combination of signal variables and a target FDR of 0.05 across 100 replications. Lasso logistic regression and the coefficient difference are used as the importance score. Lower power was observed compared to LCD applied to linearly generated $y$ as categorical $y$ contains less information. Modified FDR is controlled here by applying threshold T, since controlling FDR using the conservative threshold $T_+$ results in no power. PLSKO and its variants perform similarly to other knockoff generators.

### S1.2.2 Sensitivity analysis

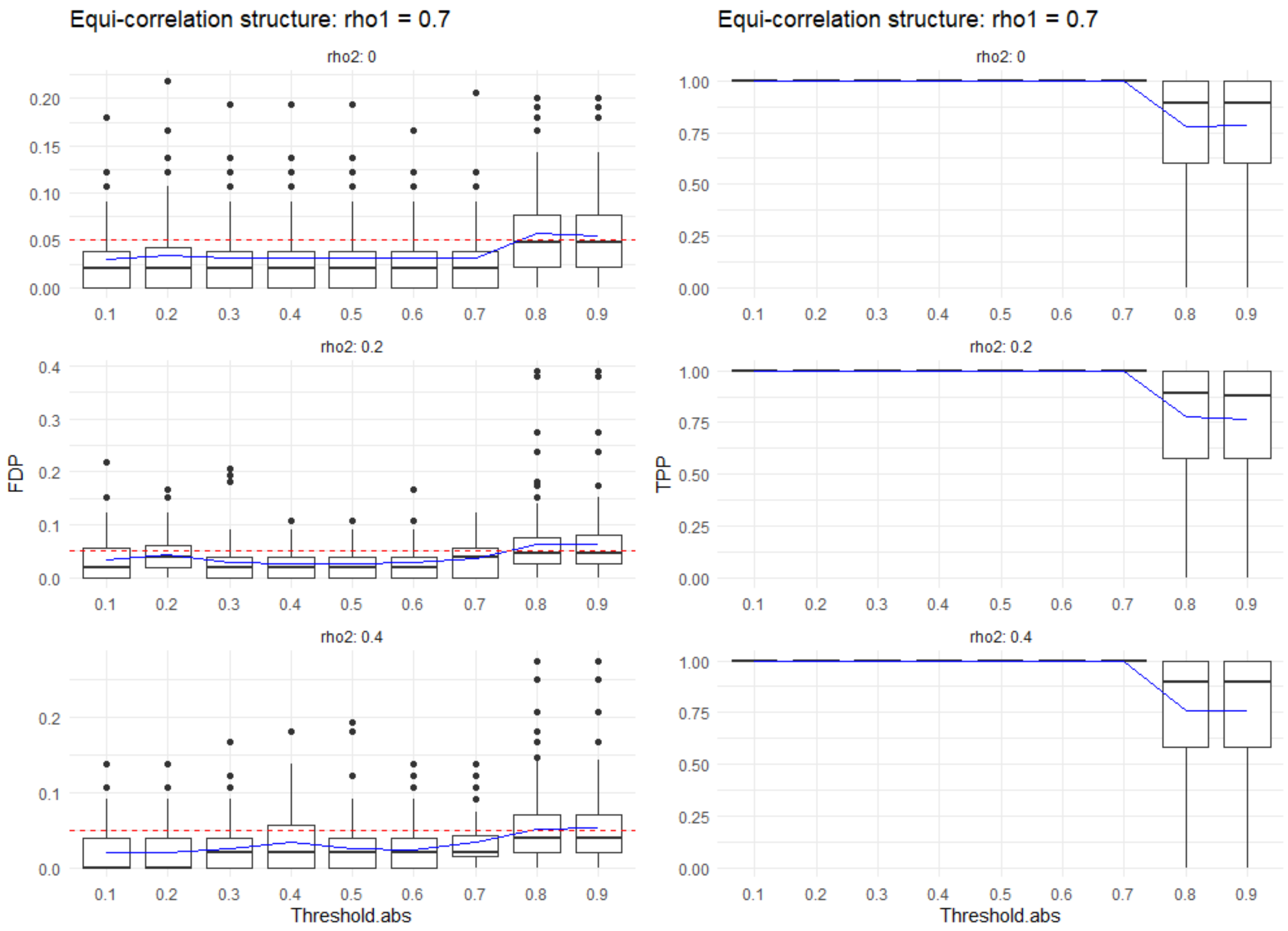


Figure S7: Simulation results of PLSKO performances under different levels of correlation between non-neighbour blocks when $X$ follows a multivariate Gaussian distribution with equi-correlation covariance w.r.t correlation levels between blocks and absolute values in the neighbour threshold. **Simulation setting**: $X$: $n = 300, p = 500(100 \times 5 blocks)$, within-block correlation $\rho_1 = 0.7$; $y$: linear model with 50 true variables, with no noise; PLSKO configurations: $ncomp = 3, q = 0.05$ no sparse PLS applied. $T_+$ is applied for knockoff filtering. The FDP and TPP are calculated over 100 repetitions. **Results:** The true neighbour threshold is 0.7, and the empirical FDP and TPP are under the targeted level when the threshold is set to or lower than 0.7. When the threshold is set to 0.8, the empirical FDP is slightly inflated. This shows the robustness of PLSKO to some extent as long as the threshold ensures all the within-block neighbours are included.

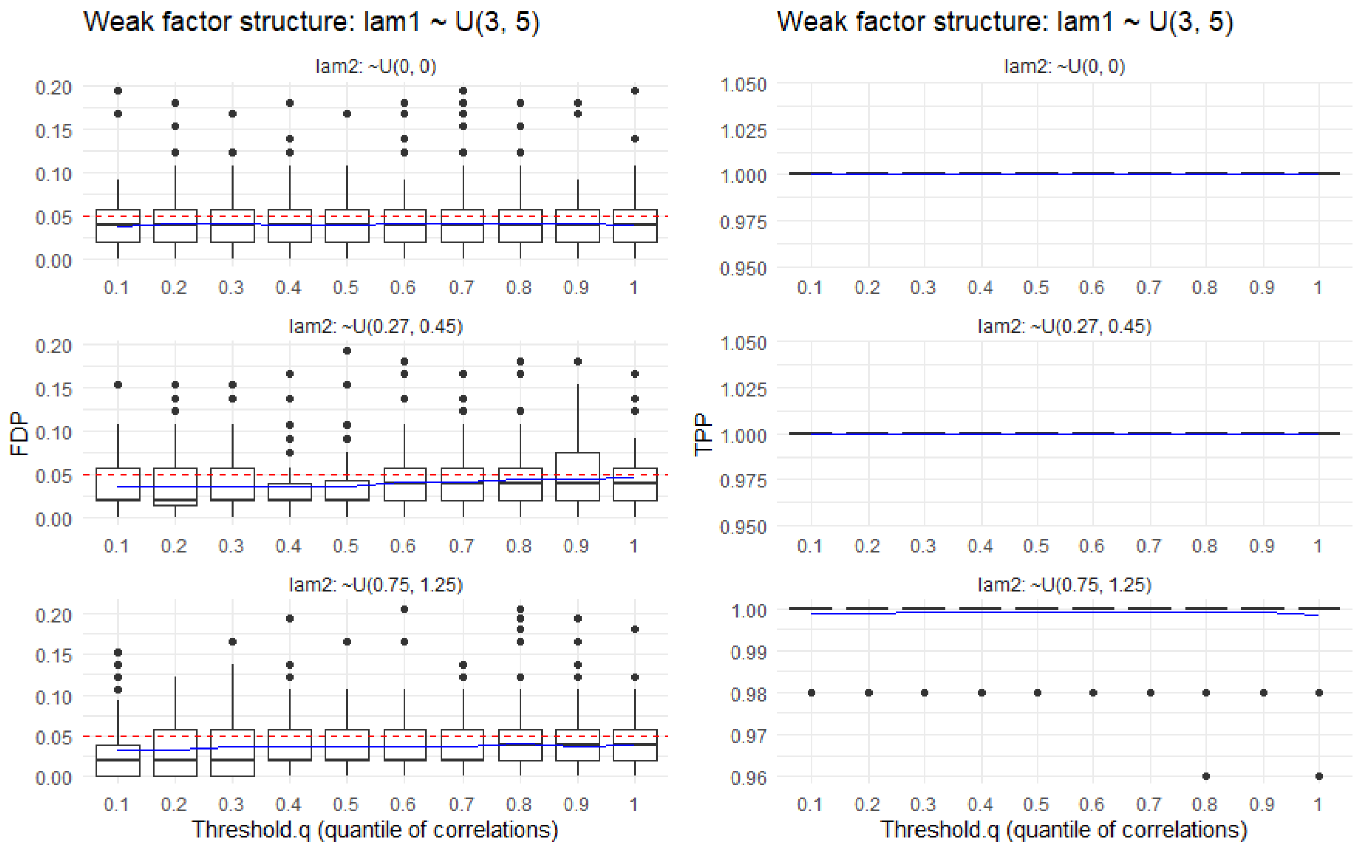


Figure S8: Figure S8: Simulation results of PLSKO performances under different levels of correlation between non-neighbour blocks when $X$ follows a Gaussian factor model with rank of 3 in each block w.r.t the amplitudes of non-zero loadings and the quantile of sample correlations as the neighbour threshold. **Simulation setting**: $X$: $n = 300, p = 500(100 \times 5blocks)$, within-block loadings $\lambda_1$ from uniform distribution $U(3, 5)$, random noise follows $N(0, 1)$; $y$: linear model with 50 true variables, with no noise. PLSKO configurations: $ncomp = 3, q = 0.05$ and no sparse PLS regression applied. $T_+$ is applied for knockoff filtering. The FDP and TPP are calculated over 100 repetitions. **Results:** The true neighbour proportions is 0.8, and the empirical FDP and TPP are under the targeted level across all the neighbour threshold settings. When the non-zero loading amplitudes increase to $U(0.75, 1.25)$, the power slightly drops. These results show the robustness of PLSKO to some extent as long as the threshold ensures all the within-block neighbours are included. This can be partially explained by the fact that although the variable and its non-neighbours are correlated, the non-neighbours are also correlated with the variable's neighbours. By controlling the neighbours, the conditional independence assumption can be satisfied empirically in this linear setting.

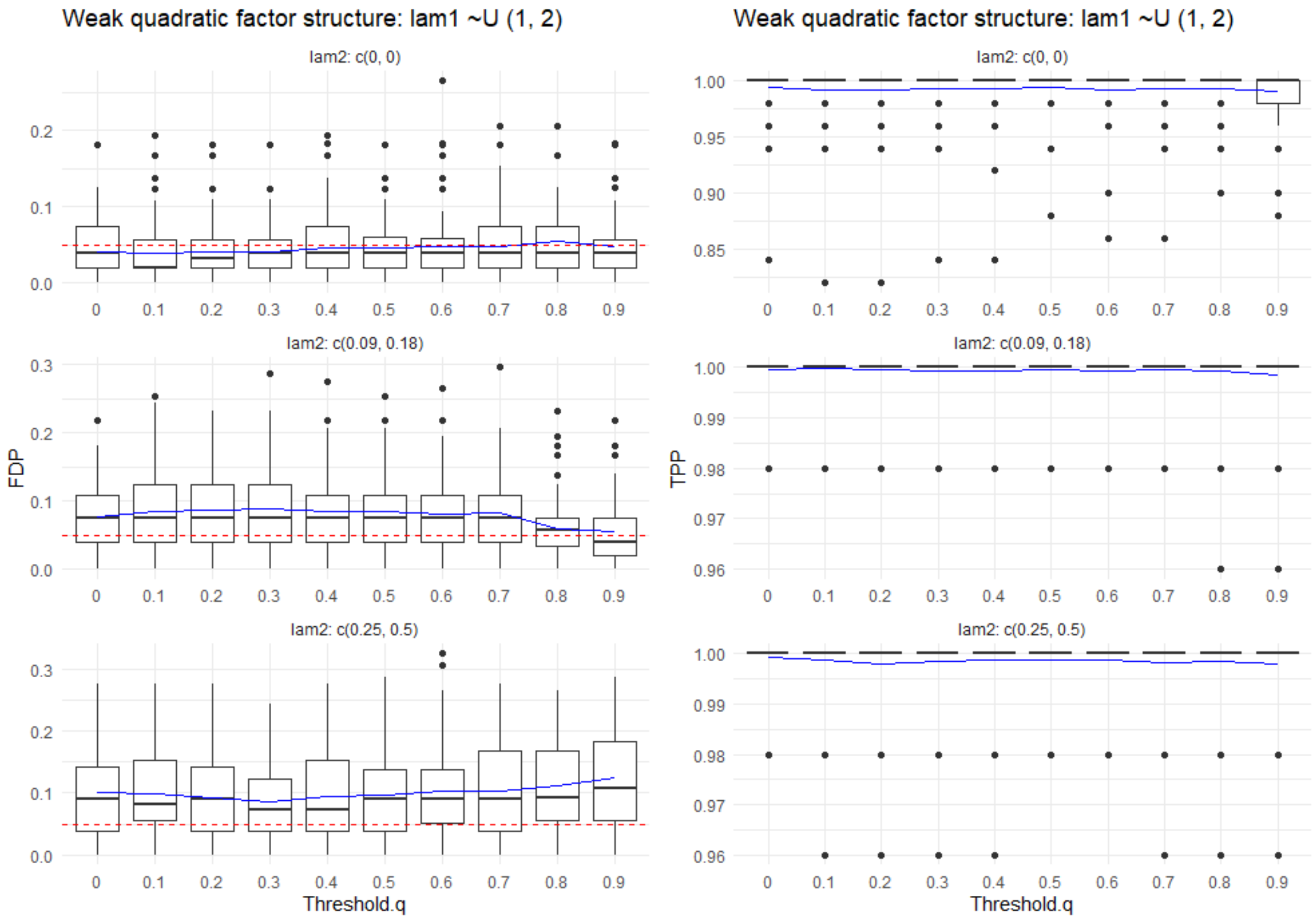


Figure S9: Simulation results of PLSKO performances under different levels of correlation between non-neighbour blocks when the half of $X$ follows a Gaussian factor model with rank of 3 and the second half is the quadratic form of the first half in each block w.r.t the amplitudes of non-zero loadings and the quantile of sample correlations as the neighbour threshold. **Simulation setting**: $X$: $n = 500, p = 500(100 \times 5blocks)$, within-block loadings $\lambda_1$ from uniform distribution $U(3, 5)$, random noise follows $N(0, 1)$ ; $y$: linear model with 50 true variables, with no noise. PLSKO configurations: $ncomp = 3, q = 0.05$ and no sparse PLS regression applied. $T_+$ is applied for knockoff filtering. The FDP and TPP are calculated over 100 repetitions. **Results:** The true neighbour proportions is 0.8, and the empirical FDP and TPP are under the targeted level only when the block correlation is zero and the neighbour proportions threshold is set to lower 0.8. The empirical FDR is inflated as the block correlation increases. This shows the limitation of PLSKO when using the sample correlation as the neighbour threshold in some cases. In non-linear relationships, the sample correlation often cannot capture the true relationship between variables, therefore excluding the true neighbours in the conditional distribution calculation. Meanwhile, the non-linear presence in the data might lead the conditional independence assumption to be violated, as the 'non-neighbours' (defined by the blocks) are no longer fully representable by the neighbours. We further investigated the cause by examining the performance of PLSKO with a pre-defined neighbour by block list in the Supplemental Figure S10.

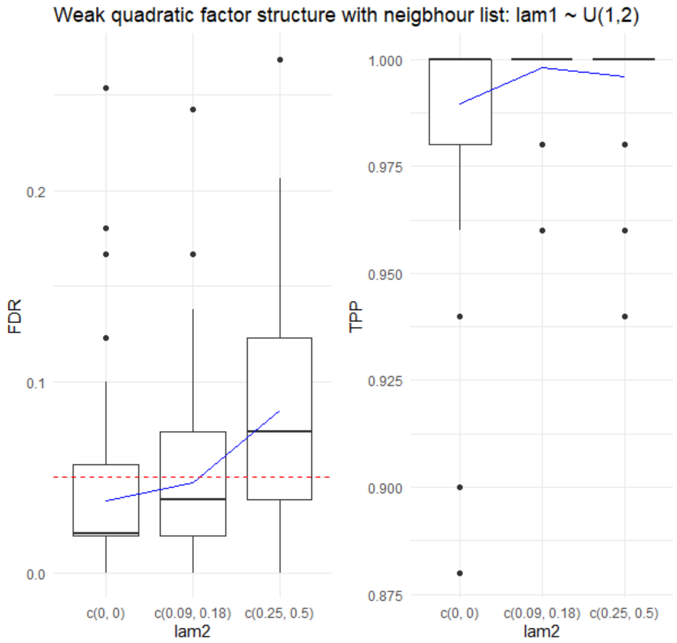


Figure S10: Simulation results of PLSKO performances under different levels of correlation between non-neighbour blocks in quadratic factor model with a pre-defined true neighbour list. Data generation and PLSKO configurations are the same as in Figure S9. The FDP and TPP are calculated over 100 repetitions. $T_+$ is applied for knockoff filtering. **Results:** By using the "true" neighbour list which is defined by the blocks, the empirical FDP has been improved across all the neighbour threshold settings. However, when the across-block correlation increased to some extent, the empirical FDP was inflated. This shows the limitation of PLSKO when the neighbourhood is poorly defined due to significant non-linear relationships between non-neighbours.

## S1.3 Semi-simulations results

### S1.3.1 Benchmark to existing knockoff generators

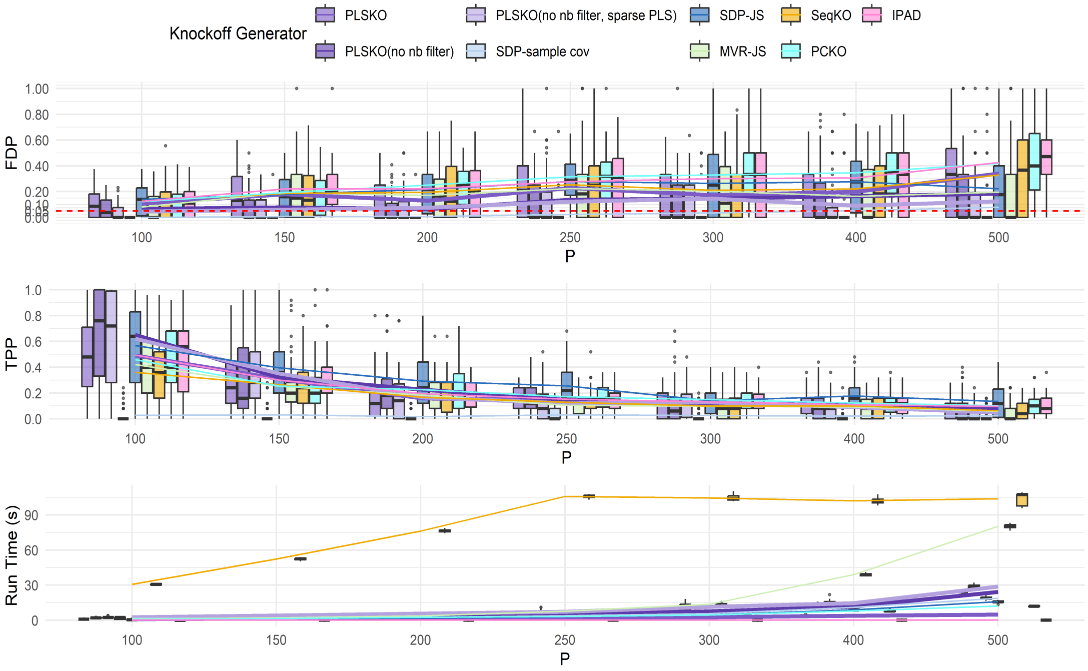


(a) Expanded result of Figure 3. Semi-simulations showing the FDR and power of PLSKO and other knockoff generators on cfRNA data across varying numbers of variables, with target FDR = 0.05, sample size $n = 71$, number of important variable $p_s = 25$ over 50 repetitions. Threshold $T$ is applied to control modified FDR. FDP: false discovery proportion; TPP: true positive proportion. Solid lines represent the mean of FDP and TPP, i.e., the estimates of FDR and power. Threshold $T$ is applied to control modified FDR.

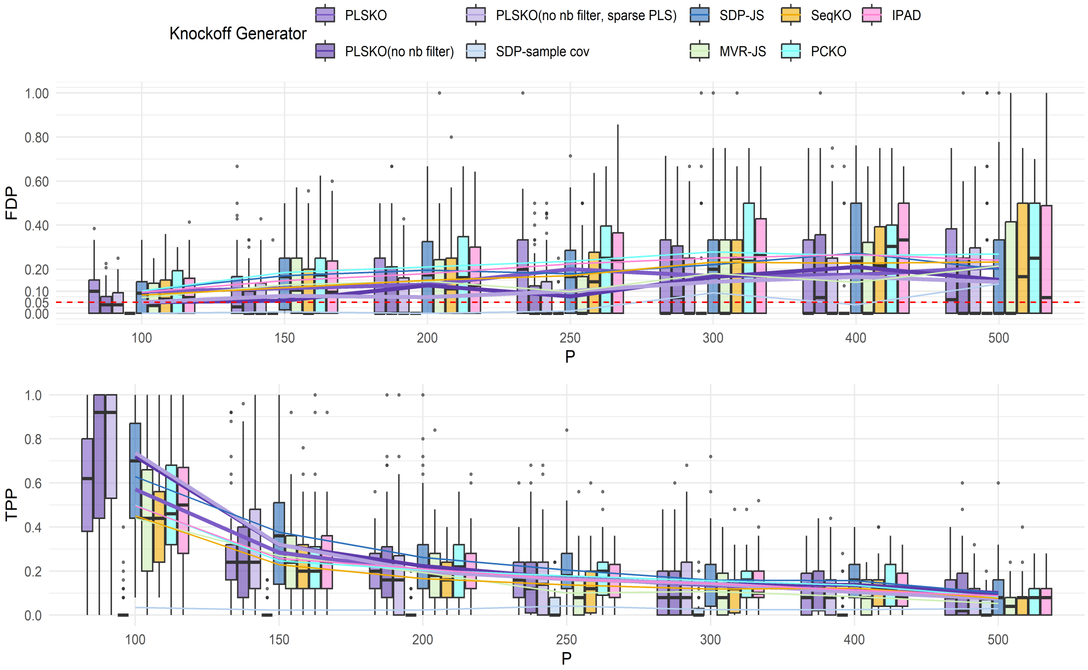


(b) Semi-simulation: performances of knockoff generators on marginally-normalised cfRNA data across varying numbers of variables, with target (modified) FDR = 0.05, sample size $n = 71$, number of important variable $p_s = 25$ over 50 repetitions. Threshold $T$ is applied to control modified FDR.

Figure S11

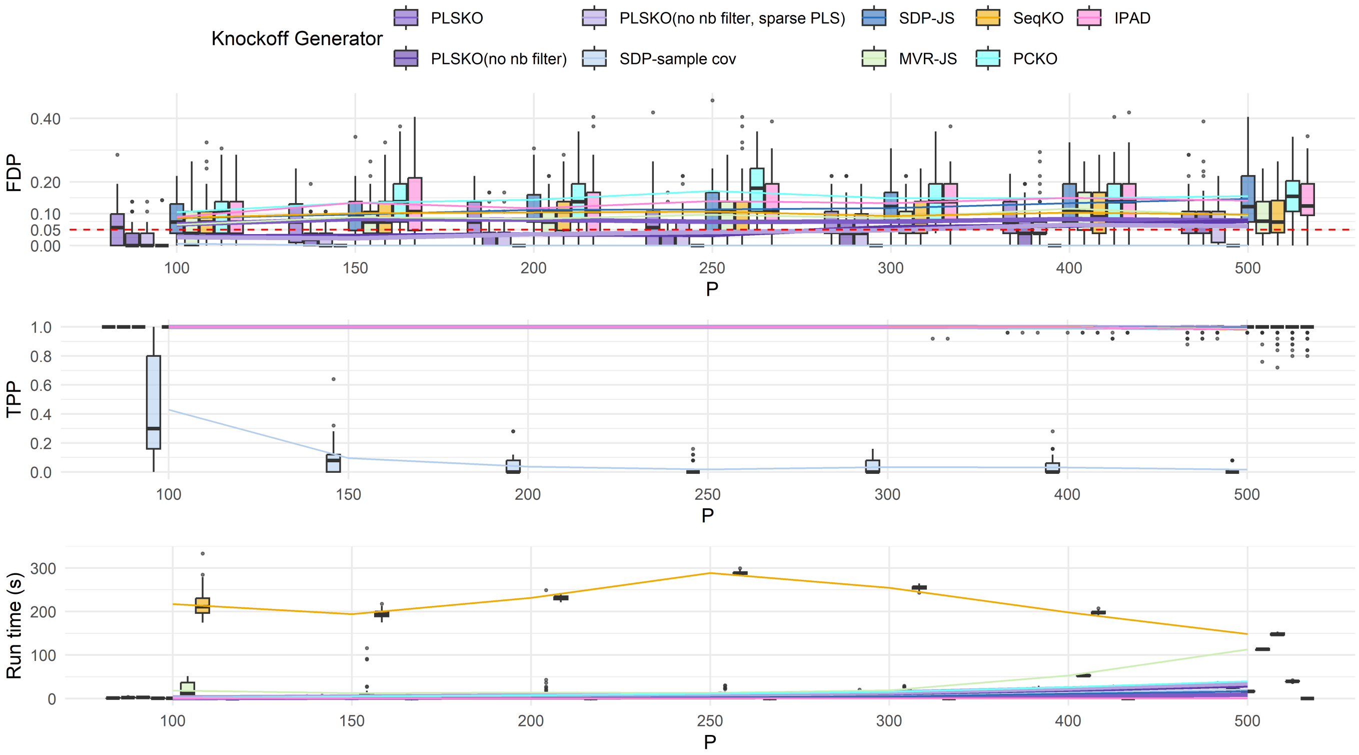


Figure S12: FDR and power of PLSKO and other knockoff generators on SomaLogic proteomics data w.r.t numbers of variables, with target FDR = 0.05, sample size $n = 166$, number of important variables $p_s = 25$, over 50 repetitions. Threshold $T$ is applied to control the modified FDR.

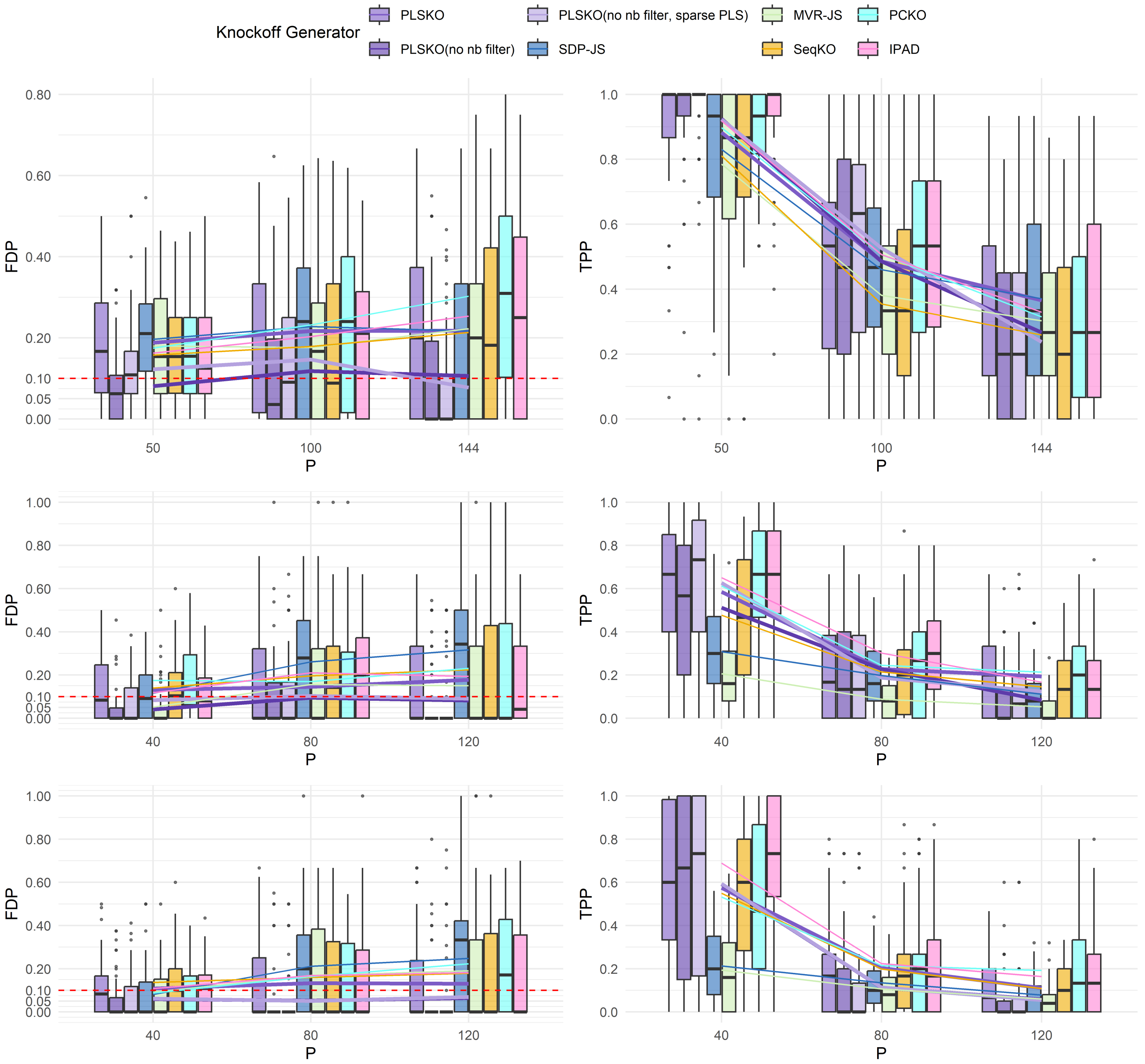


Figure S13: Semi-simulations: FDR and power of PLSKO and other knockoff generators on the microbiome (upper), proteomics (middle) and urine metabolome (lower) data across varying numbers of variables, with target FDR $= 0.10$, sample size $n = 36$, number of important variable $p_s = 15$ over 50 repetitions. Threshold $T$ is applied to control modified FDR. FDP: false discovery proportion; TPP: true positive proportion. Solid lines represent the mean of FDP and TPP, i.e., the estimates of FDR and power.

### S1.3.2 Benchmark to common variable selection methods

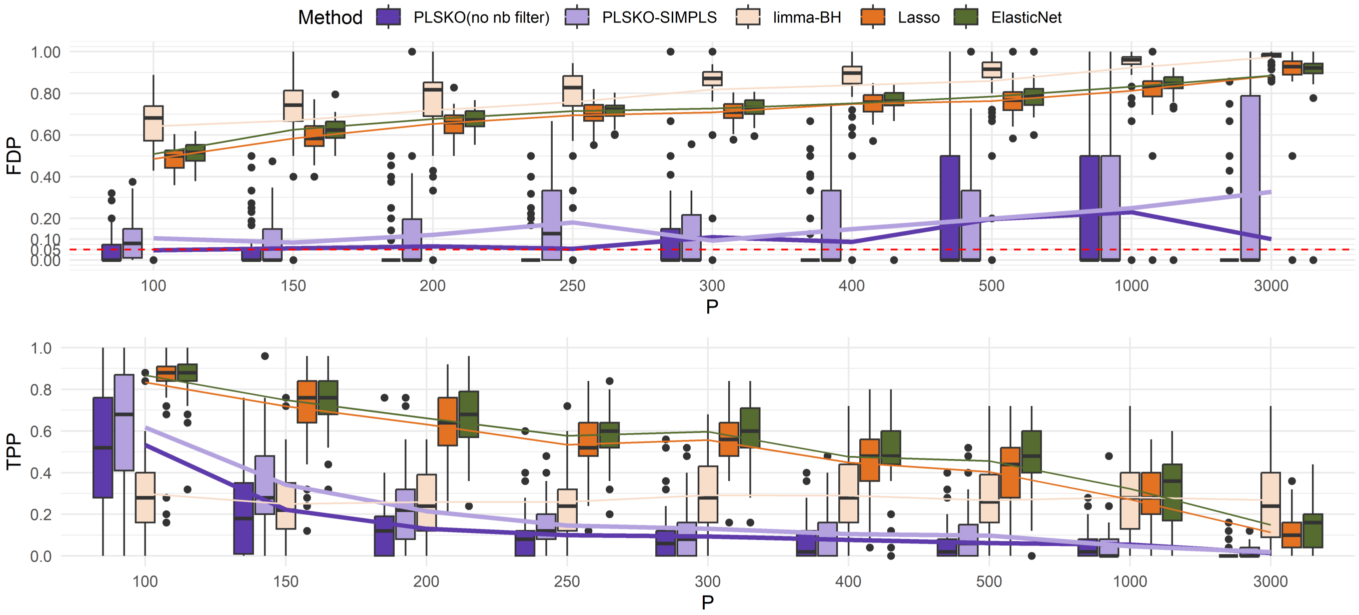


| Run time (s, Mean(SD)) | P=100 | P=150 | P=200 | P=250 | P=300 | P=400 | P=500 | P=1000 | P=3000 |
|---|---|---|---|---|---|---|---|---|---|
| *PLSKO(no nb filter)* | 7.351(0.94) | 9.802(1.152) | 12.457(1.309) | 13.347(0.876) | 15.573(0.775) | 21.041(0.751) | 29.009(0.91) | 87.966(2.152) | 620.717(43.005) |
| *PLSKO-SIMPLS* | 9.091(2.099) | 8.304(1.105) | 9.534(1.023) | 9.738(0.632) | 11.57(0.501) | 15.669(0.494) | 20.613(0.639) | 61.827(0.971) | 438.679(5.612) |

Figure S14: Semi-simulation (linear response variable) results of FDP, TPP and run time of PLSKO compared to Lasso, ElasticNet, limma and Wilcoxon test with Benjamini-Hochberg adjustment on the cfRNA data w.r.t. number of variables. **Simulation setting**: $X$: $n = 71$; $y$: linear combination with 25 true variables, with no noise. PLSKO configurations: $ncomp = 6, threshold.abs = 0, q = 0.05$ and no sparse PLS regression applied. $T$ is applied for knockoff filtering. The FDP and TPP are calculated over 50 repetitions. **Results:** As expected, Lasso and ElasticNet failed in FDR control since these models do not include an FDR control procedure. limma with BH approach also failed to control FDR, since limma tests on marginal association between each variable and the response, which leads to the selection of not only the true signal variables that generate $y$, but also other variables that are correlated with them. PLSKO has a better FDP control than Lasso, ElasticNet and limma with BH adjustment and a much lower TPP to Lasso and ElasticNet. Regarding computational time, PLSKO is slower than Lasso, ElasticNet, and limma. These methods can be done running less than a second, while PLSKO might take several minutes depending on the variable size and parameters. We also tested PLSKO-SIMPLS, which implements the SIMPLS algorithm (See Supplemental Section S3.4) and drastically reduces computational time.

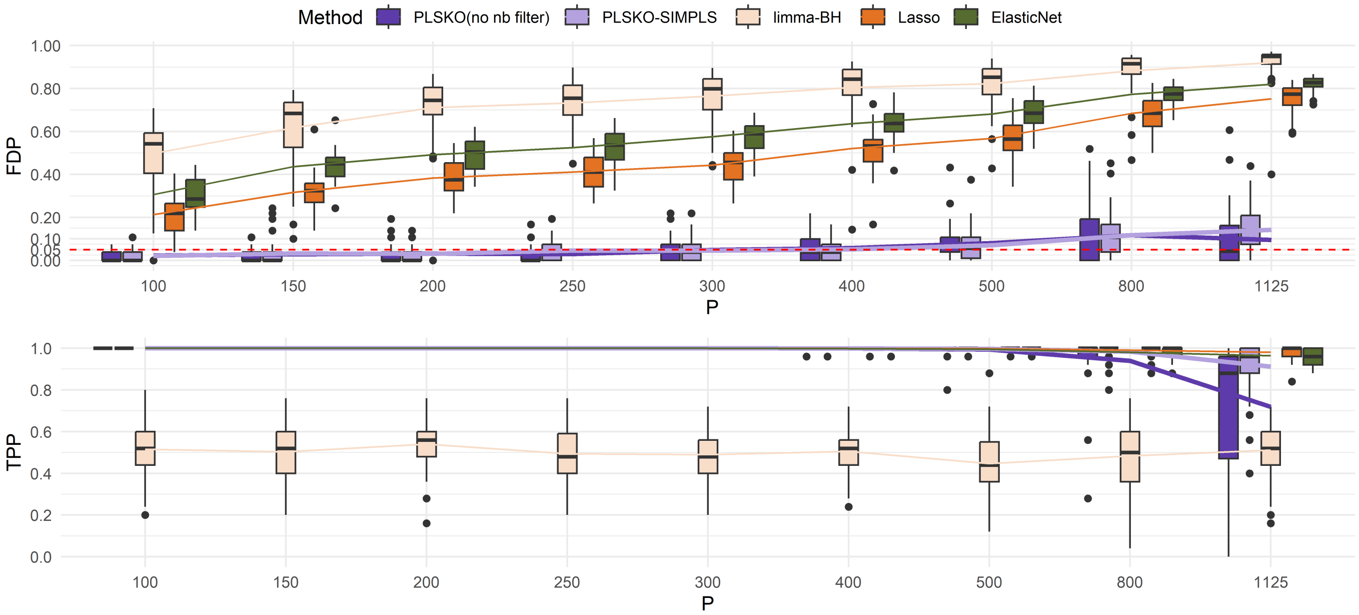


| Run time (s, Mean(SD)) | P=100 | P=150 | P=200 | P=250 | P=300 | P=400 | P=500 | P=800 | P=1125 |
|---|---|---|---|---|---|---|---|---|---|
| *PLSKO(no nb filter)* | 6.909(0.77) | 8.655(0.95) | 11.358(1.162) | 13.236(0.894) | 16.383(1.468) | 25.774(1.738) | 38.459(2.222) | 95.05(2.453) | 169.82(8.366) |
| *PLSKO-SIMPLS* | 7.354(0.657) | 8.648(0.721) | 10.809(0.636) | 12.598(0.73) | 14.924(0.555) | 22.633(1.183) | 33.183(0.651) | 76.947(1.418) | 131.79(8.074) |

Figure S15: Semi-simulation results with linear generated response variable of FDP, TPP and run time of PLSKO compared to Lasso, ElasticNet, limma and Wilcoxon test with Benjamini-Hochberg adjustment on the proteomics data w.r.t. number of variables. **Simulation setting**: $X$: $n = 166$, other settings are the same as in Figure S12. **Results:** Similar to the cfRNA data, Lasso and ElasticNet failed in FDR control, limma with BH adjustment also failed to control FDR. PLSKO has a better FDP control than Lasso, ElasticNet and limma with BH adjustment, especially when the number of variables is less than 500. Power on this data is much better than with the cfRNA data.

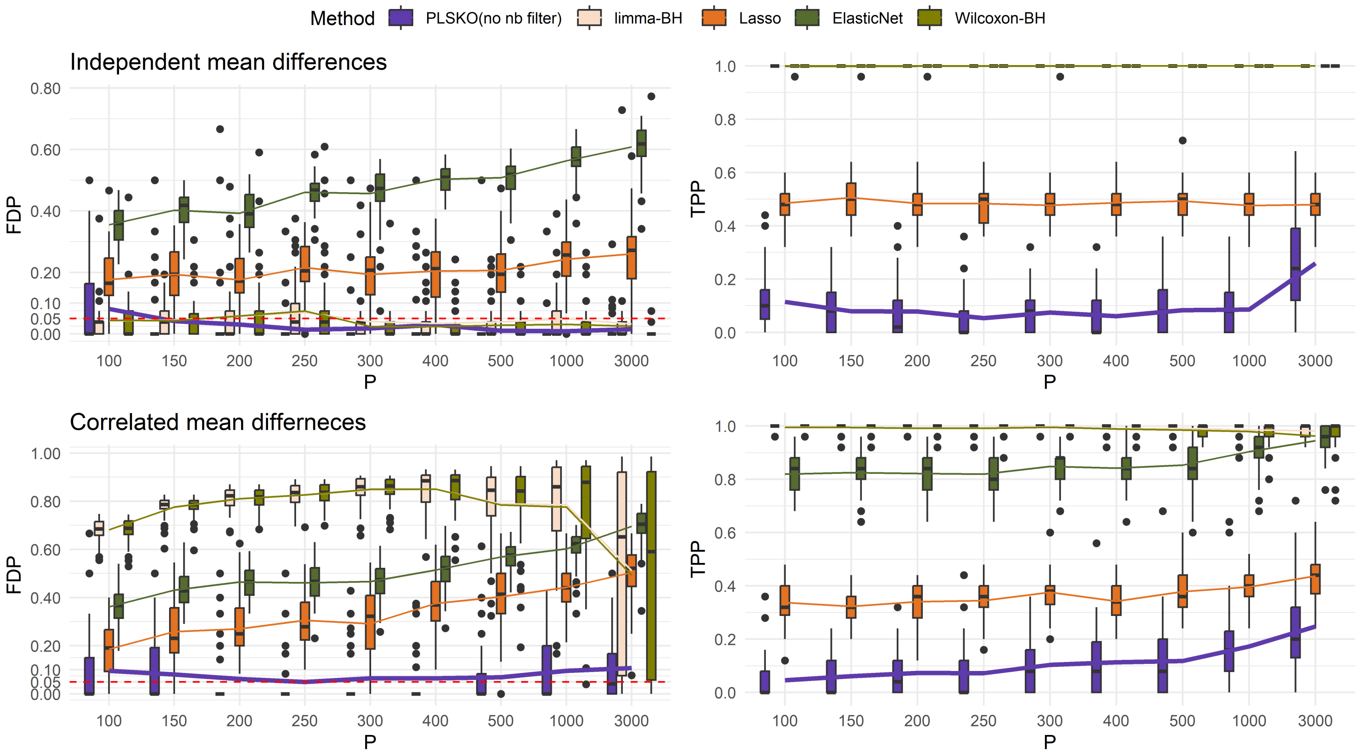


Figure S16: Figure S16: Semi-simulation results on cfRNA data, with different means of important variables between the two groups w.r.t. numbers of variables. **Simulation setting**: $y$ (group assignment): $n = 71$, sampled from $Beroulli(0.5)$; $X$ was scaled and a subset of 25 important variables was randomly selected. For the observations $y = 1$, a mean difference $Z$ was added to the important variables: In the upper panel, the mean differences are independent, sampled from $N(3, 1)$. In the lower panel, the mean differences ($\beta$) are correlated, drawn from a multivariate normal distribution $MVN(\mu, \Sigma)$, where $\mu$ is the mean vector (with elements set to 3 for important variables and 0 otherwise) and $\Sigma$ is the empirical covariance matrix of $X$, therefore, preserving the correlation structure among predictors. PLSKO configurations: $ncomp = 6, threshold.abs = 0, q = 0.05$ and no sparse PLS regression applied. $T$ is applied for knockoff filtering. The FDP and TPP are calculated over 50 repetitions. **Results:** As shown in the upper panel, when the mean differences are independent, limma and Wilcoxon test with BH adjustment generally controlled the FDR under the targeted level and achieved very high power, while Lasso and ElasticNet failed to control the FDR. PLSKO with lasso logistic regression also controlled the FDR but with very low power. In the lower panel, when the mean differences are correlated, all the other methods failed to control the FDR, while PLSKO achieved FDR generally around the targeted level and very low power.

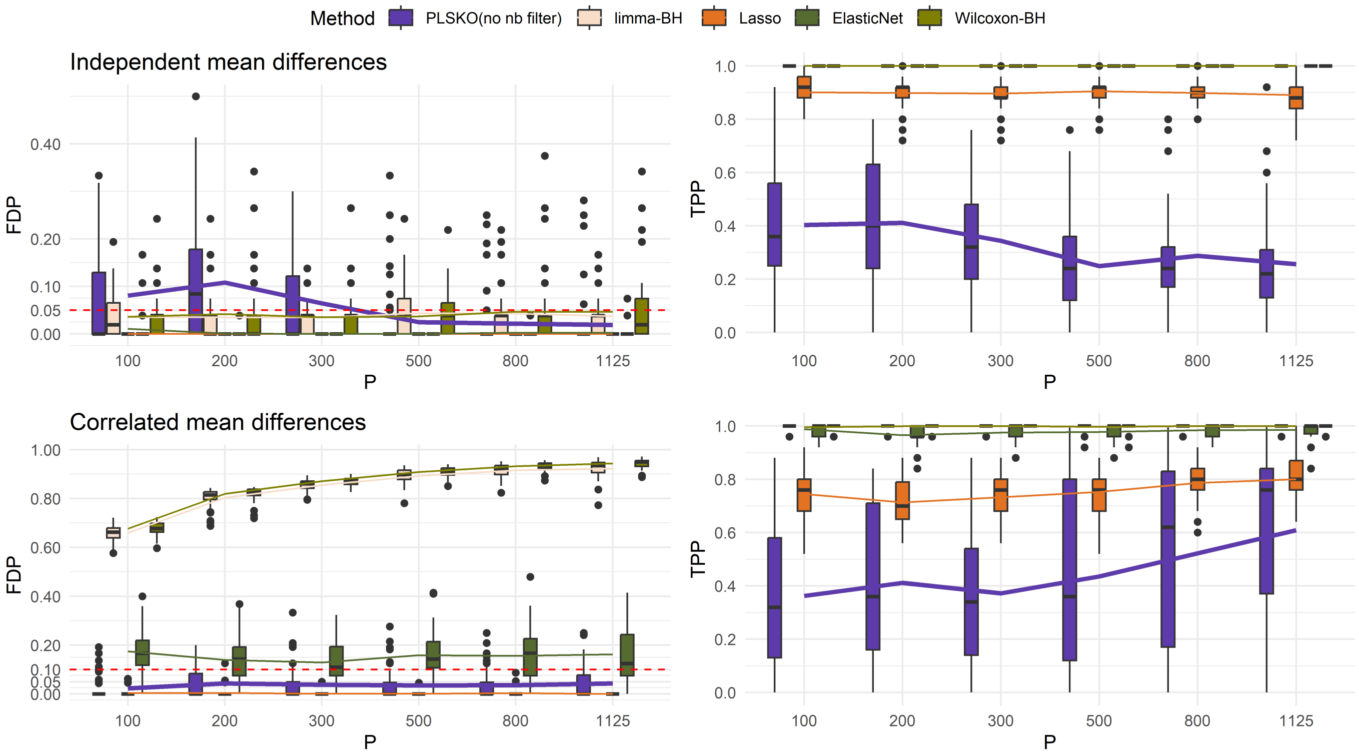


Figure S17: Figure S17: Semi-simulation on SomaLogic proteomics data, where important variables have differing means between the two groups, shown w.r.t to the number of variables. **Simulation settings:** Group assignment ($y$): $n = 166$. Other settings are identical to those in Figure S16. **Results:** The results were generally consistent with Figure S16, except that lasso exhibited improved FDR control in both conditions. This improvement is likely due to the increased separation between important and null variables. Overall, Figures S14–S17 demonstrate that PLSKO maintains robust FDR control regardless of assumptions about the relationship between $X$ and $y$ or the data type, making it a hypothesis-free approach. In contrast, the FDR control of other methods varies depending on the underlying hypothesis.

## S1.4 Tuning parameter for case studies

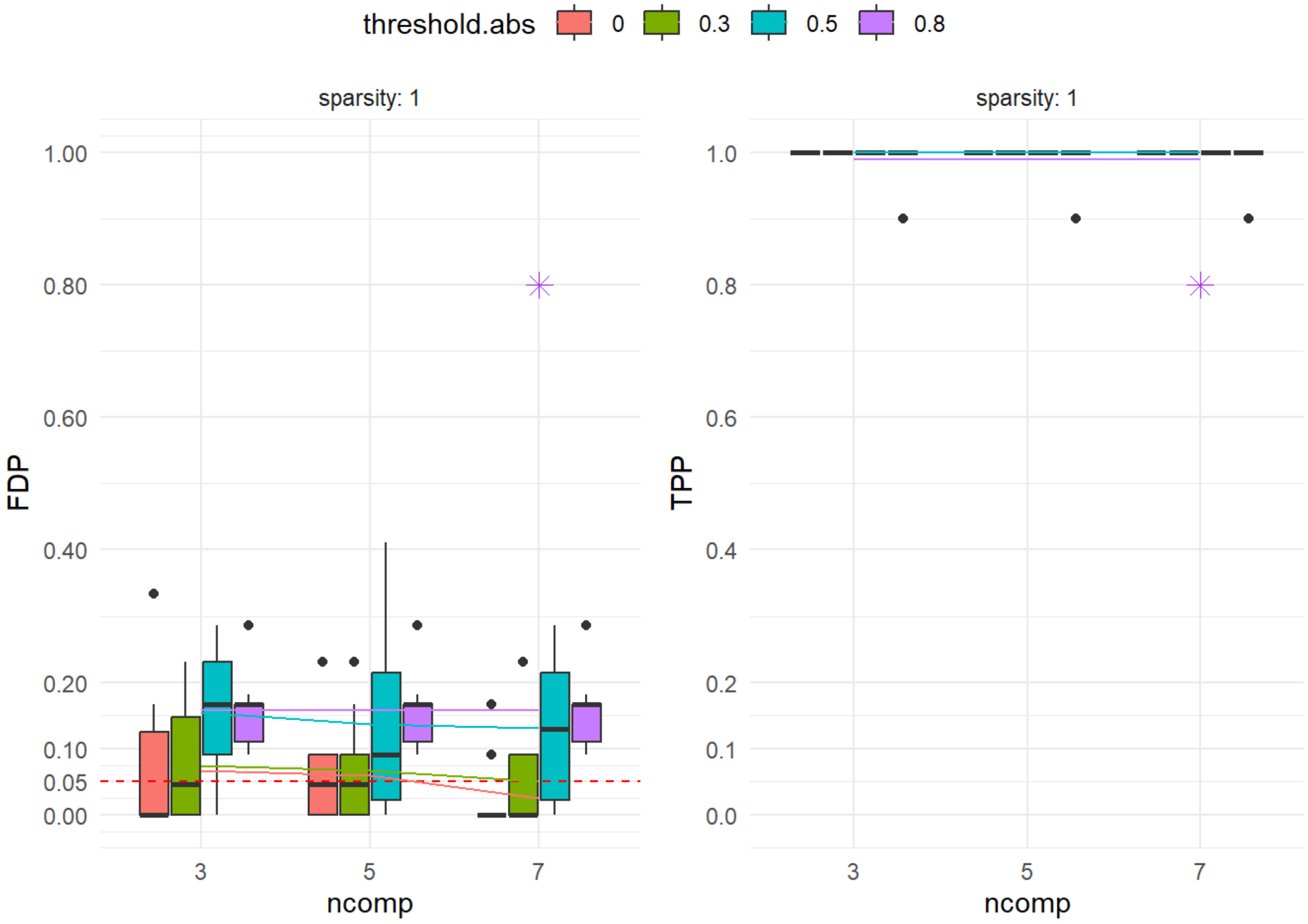


Figure S18: Tuning results of PLSKO on the cfRNA data. Tuning settings: the number of repetitions for each configuration: $n_{ko} = 25$, the proportions of important variables: 10%. The optimal configuration is marked by the purple asterisk. The optimal configuration (number of components, ncomp = 7; neighbour correlation absolute threshold, threshold.abs = 0.3) is used in the case study.

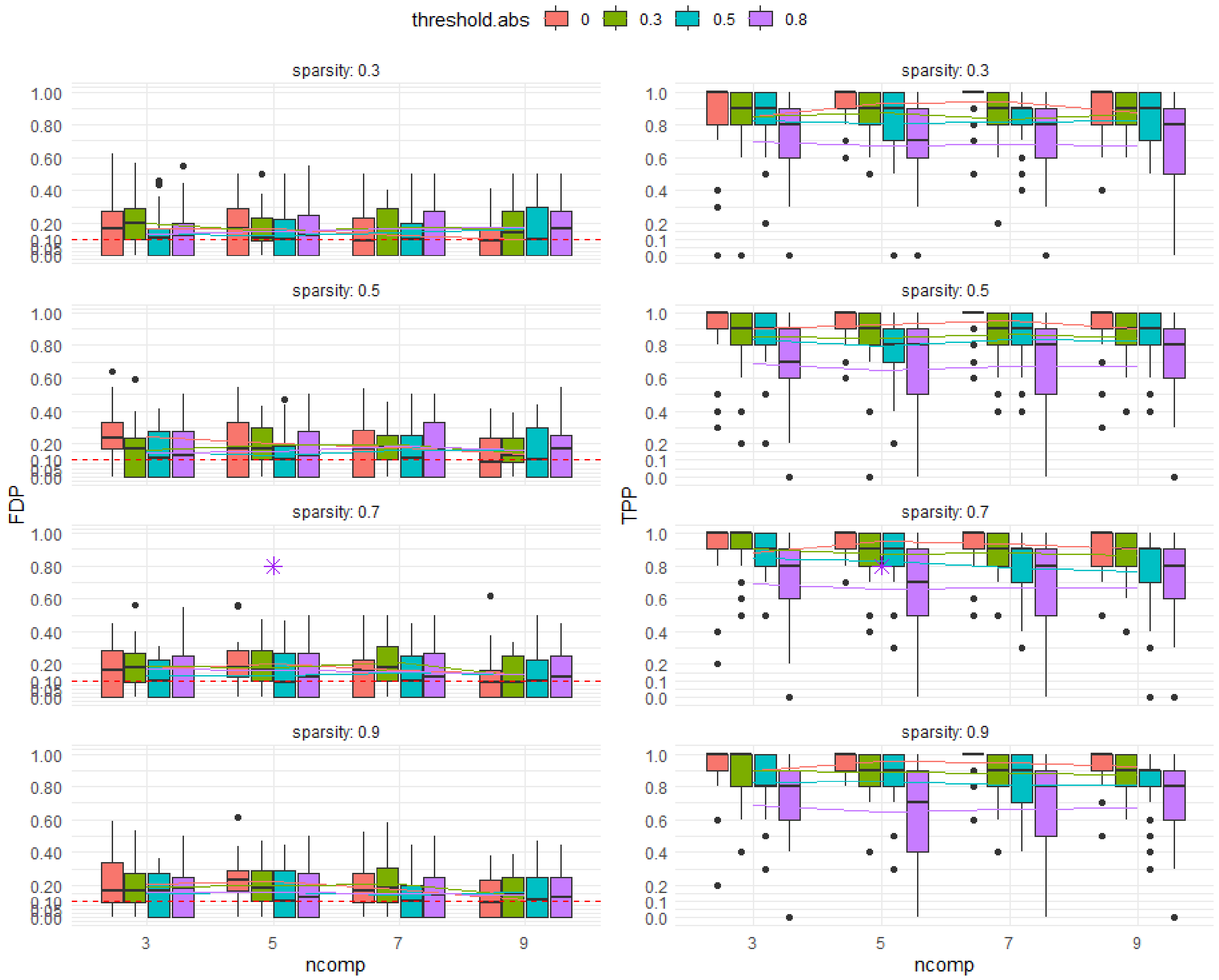


Figure S19: Tuning results of PLSKO on the proteomics data from the multiomics dataset used in the case study. Tuning settings: the number of repetitions for each configuration: $n_{ko} = 25$, the proportions of important variables: 10%. The optimal configuration (number of components, ncomp = 5; neighbour correlation absolute threshold, threshold.abs = 0.5; the proportion of variables kept in the sparse PLS regression, sparsity = 0.7) is applied in the case study.

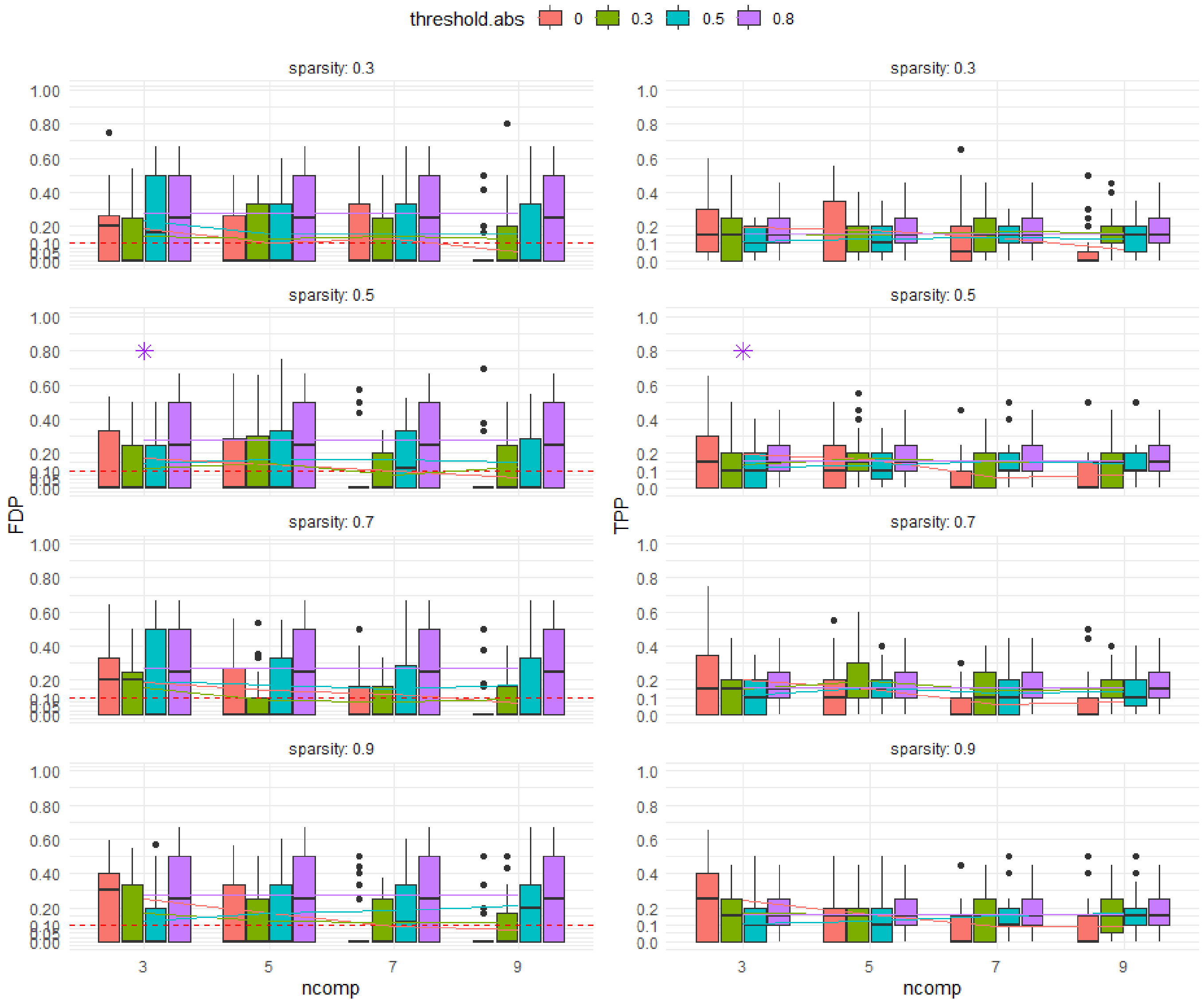


Figure S20: Tuning results of PLSKO on the microbiome data from the multiomics dataset used in the case study. Tuning settings: the number of repetitions for each configuration: $n_{ko} = 25$, the proportions of important variables: 10%. The optimal configuration (number of components, ncomp = 3; neighbour correlation absolute threshold, threshold.abs = 0.5; the proportion of variables kept in the sparse PLS regression, sparsity = 0.5) is applied in the case study.

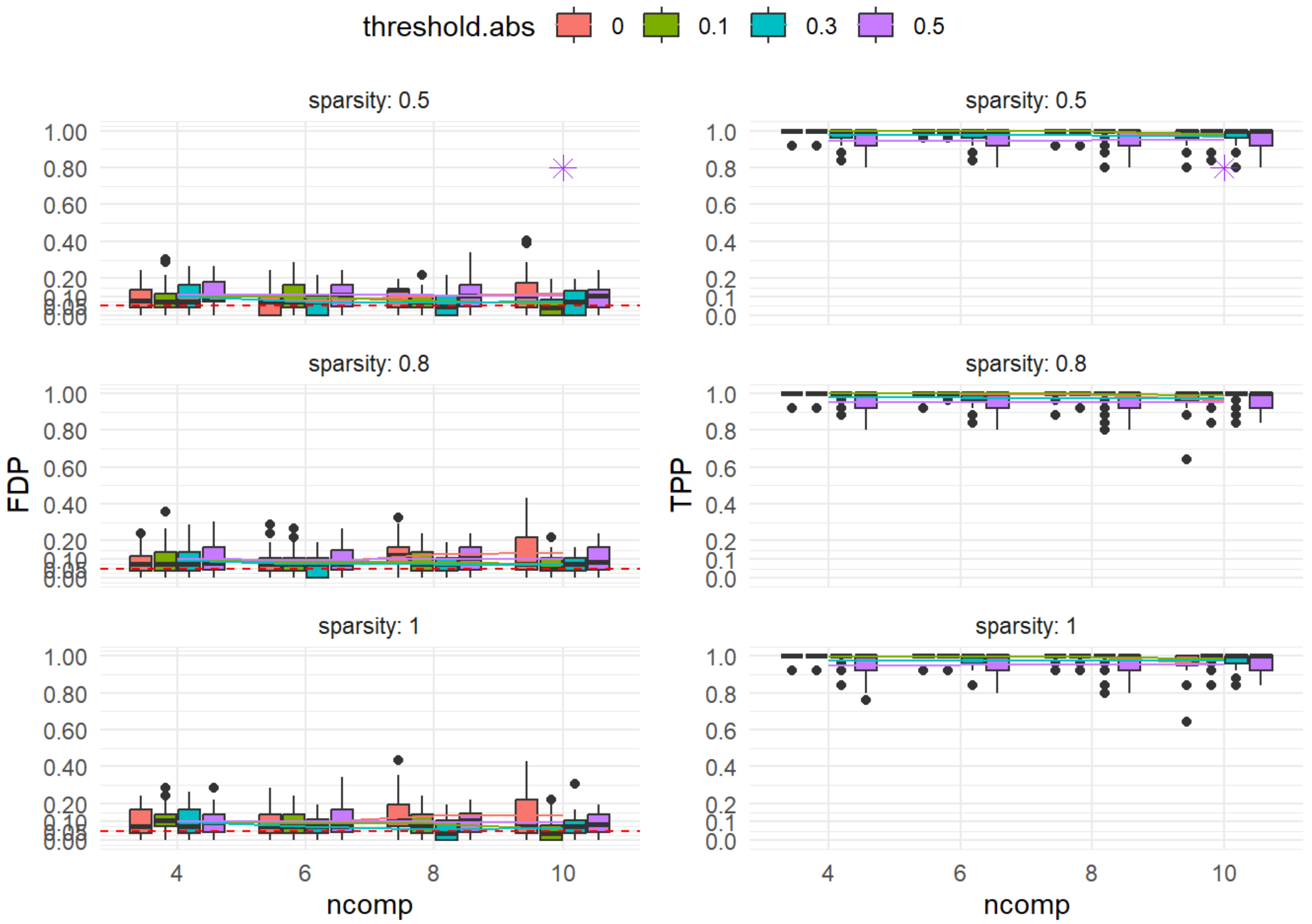


Figure S21: Tuning results of PLSKO on the SomaLogic proteomics data. Tuning settings: the number of repetitions for each configuration: $n_{ko} = 25$, the proportions of important variables: 10%. The optimal configuration is marked by the purple asterisk.

# S2 Supplementary Tables

Table S1: Selected preeclampsia-related features from alternative subsets of the omics data over 50 generated knockoffs

| **Dataset** | **Predictors** | **Selected Feature** | **PLSKO frequency** | **Selected by PLS-AKO** | **DE by other methods** |
|---|---|---|---|---|---|
| cell-free Transcriptomics from multi-omics data (n = 36) | Placenta-specific elevated gene expression (p = 77) | IGF2 | 0.04 | Yes | No |
| | | BPGM | 0.04 | No | No |
| | | HBM | 0 | No | Yes (limma) |
| | Placenta-specific (fold-change > 3) elevated gene (p = 86) (HPA v23) | IGF2 | 0.06 | Yes | No |
| | | EMILIN2 | 0.06 | No | No |
| cell-free Transcriptomics (n = 71) | Placenta-specific elevated gene expression (p = 96) (HPA v23) | MBNL3 | 0.44 | Yes | Yes (Wilcoxon) |
| | | CD36 | 0.42 | Yes | No |
| | | PHACTR2 | 0.18 | No | No |
| | | FLI1 | 0.12 | No | No |
| | | CDK6 | 0.1 | No | No |
| | Genes with the highest variance (p = 200) | CA1 | 0.42 | Yes | No |
| | | RN7SL564P | 0.38 | Yes | Yes (limma, Wilcoxon) |
| | | RN7SL381P | 0.34 | Yes | Yes (limma, Wilcoxon) |
| | | MAGI2-AS3 | 0.3 | No | No |
| | | RN7SL736P | 0.14 | No | Yes (limma, Wilcoxon) |
| | | RNA5SP387 | 0.1 | No | No |
| Proteomics from multi-omics study (n = 36) | Placenta-specific elevated gene expression (p = 101) (HPA v23) | IL1RAP | 0.36 | Yes | No |
| | | COLEC12 | 0.36 | Yes | No |
| | | HAPLN1 | 0 | No | Yes (Wilcoxon) |
| | Placenta-specific elevated gene expression (p = 63) (HPA v19) | IL1RAP | 0.58 | Yes | Yes (limma) |
| | | LEP | 0.58 | Yes | Yes (limma, Wilcoxon) |
| | | GSTA3 | 0.1 | No | No |
| | | HAPLN1 | 0 | No | Yes (Wilcoxon) |
| Microbiome data from a multi-omics study (N = 49) | 144 OTUs (TMM normalisation) | s_L. acidophilus | 0.04 | Yes | No |
| | | s_L. iners | 0.04 | No | No |

Table S2: Selected variables in the cell-free RNA dataset (without subselection on placenta elevated variables) by PLSKO, limma and Wilcoxon test [1]

| **Datasets: cell-free RNA , 3,000 genes with the highest variance, n = 71** | | | | | |
|---|---|---|---|---|---|
| **limma significant** | | **PLSKO** | | **Frequency** | **PLSKO-AKO** |
| ENSG00000211896 | IGHG1 | ENSG00000263968 | RN7SL381P | 0.22 | T |
| ENSG00000239437 | RN7SL752P | ENSG00000240606 | RN7SL564P | 0.18 | F |
| ENSG00000239607 | RN7SL573P | ENSG00000119048 | UBE2B | 0.08 | F |
| ENSG00000239899 | RN7SL674P | | | | |
| ENSG00000240606 | RN7SL564P | | | | |
| ENSG00000240869 | RN7SL128P | | | | |
| ENSG00000241529 | RN7SL767P | | | | |
| ENSG00000243352 | RN7SL8P | | | | |
| ENSG00000244642 | RN7SL396P | | | | |
| ENSG00000251705 | RNA5-8SP6 | | | | |
| ENSG00000263968 | RN7SL381P | | | | |
| ENSG00000264169 | RN7SL665P | | | | |
| ENSG00000264275 | RN7SL753P | | | | |
| ENSG00000264916 | RN7SL230P | | | | |
| ENSG00000264978 | RN7SL630P | | | | |
| ENSG00000265735 | RN7SL5P | | | | |
| ENSG00000266439 | RN7SL493P | | | | |
| ENSG00000266794 | RN7SL7P | | | | |
| ENSG00000275803 | RN7SL736P | | | | |

| **Datasets: cell-free RNA, all 7,160 genes after prefiltering, n = 71** | | | | | |
|---|---|---|---|---|---|
| **limma** | | **PLSKO** | | **Frequency** | **PLSKO-AKO** |
| ENSG00000013561 | RNF14 | ENSG00000263968 | RN7SL381P | 0.2 | F |
| ENSG00000239437 | RN7SL752P | ENSG00000013561 | RNF14 | 0.18 | F |
| ENSG00000239607 | RN7SL573P | ENSG00000103342 | GSPT1 | 0.18 | F |
| ENSG00000239899 | RN7SL674P | ENSG00000240606 | RN7SL564P | 0.16 | F |
| ENSG00000240606 | RN7SL564P | ENSG00000109787 | KLF3 | 0.12 | F |
| ENSG00000240869 | RN7SL128P | ENSG00000163875 | MEAF6 | 0.12 | F |
| ENSG00000241529 | RN7SL767P | | | | |
| ENSG00000243352 | RN7SL8P | | | | |
| ENSG00000244642 | RN7SL396P | | | | |
| ENSG00000263968 | RN7SL381P | | | | |
| ENSG00000264169 | RN7SL665P | | | | |
| ENSG00000264275 | RN7SL753P | | | | |
| ENSG00000264916 | RN7SL230P | | | | |
| ENSG00000264978 | RN7SL630P | | | | |
| ENSG00000265735 | RN7SL5P | | | | |
| ENSG00000266439 | RN7SL493P | | | | |
| ENSG00000275803 | RN7SL736P | | | | |

| **Datasets: cell-free RNA, 200 variables with the highest variance, n = 71** | | | | | | | |
|---|---|---|---|---|---|---|---|
| **limma** | | **PLSKO** | | **Frequency** | **PLSKO-AKO** | **Wilcoxon** | |
| ENSG00000200434 | RNA5-8SP2 | ENSG00000133742 | CA1 | 0.42 | T | ENSG00000239437 | RN7SL752P |
| ENSG00000239437 | RN7SL752P | ENSG00000240606 | RN7SL564P | 0.38 | T | ENSG00000239607 | RN7SL573P |
| ENSG00000239607 | RN7SL573P | ENSG00000263968 | RN7SL381P | 0.34 | T | ENSG00000239899 | RN7SL674P |
| ENSG00000239899 | RN7SL674P | ENSG00000234456 | MAGI2-AS3 | 0.3 | F | ENSG00000240606 | RN7SL564P |
| ENSG00000240606 | RN7SL564P | ENSG00000275803 | RN7SL736P | 0.14 | F | ENSG00000240869 | RN7SL128P |
| ENSG00000240869 | RN7SL128P | ENSG00000201096 | RNA5SP387 | 0.1 | F | ENSG00000241529 | RN7SL767P |
| ENSG00000241529 | RN7SL767P | | | | | ENSG00000243352 | RN7SL8P |
| ENSG00000243352 | RN7SL8P | | | | | ENSG00000244230 | RN7SL151P |
| ENSG00000244230 | RN7SL151P | | | | | ENSG00000244642 | RN7SL396P |
| ENSG00000244642 | RN7SL396P | | | | | ENSG00000251705 | RNA5-8SP6 |
| ENSG00000251705 | RNA5-8SP6 | | | | | ENSG00000263968 | RN7SL381P |
| ENSG00000263968 | RN7SL381P | | | | | ENSG00000264169 | RN7SL665P |
| ENSG00000264169 | RN7SL665P | | | | | ENSG00000264275 | RN7SL753P |
| ENSG00000264275 | RN7SL753P | | | | | ENSG00000264916 | RN7SL230P |
| ENSG00000264916 | RN7SL230P | | | | | ENSG00000264978 | RN7SL630P |
| ENSG00000264978 | RN7SL630P | | | | | ENSG00000265735 | RN7SL5P |
| ENSG00000265735 | RN7SL5P | | | | | ENSG00000266439 | RN7SL493P |
| ENSG00000266439 | RN7SL493P | | | | | ENSG00000275803 | RN7SL736P |
| ENSG00000275803 | RN7SL736P | | | | | | |

[1] a. No DEG were detected by Wilcoxon test in either 3,000 genes or the whole dataset; b. Pseudogenes (e.g. RN7SL752P) tended to be selected together by the marginal tests as the genes are highly correlated (e.g. correlation > 0.85), while PLSKO selected only one or two pseudogenes as other pseudogenes have been controlled;

Table S3: Selected genes co-expressed with gene RNF14 from the placenta-elevated genes in the cell-free plasma data. The gene RNF14 was identified as DE in preeclampsia cell-free plasma (Table S2). Marginal DE tests are not applicable to this case with a non-categorical response variable.

| Dataset | Predictors | Selected Feature | Frequency by PLSKO | Selected by PLS-AKO |
|---|---|---|---|---|
| Cell-free transcriptomics (n = 71) | Placenta-specific elevated gene (p = 81) | BPGM | 0.84 | Yes |
| | | MBNL3 | 0.8 | Yes |
| | | GM2A | 0.32 | Yes |
| | | TXK | 0.28 | No |
| | | FAM46A | 0.24 | No |
| | | MYLIP | 0.2 | No |

Table S4: Linear regression of expression level of gene *PHACTR2* on group of preeclampsia (PHACTR2 = $\alpha$+$\beta$ PE)

| | *Dependent variable:* |
|---|---|
| | PHACTR2 |
| PE | −0.428 |
| | (0.216) |
| Intercept | 6.265*** |
| | (0.103) |
| Observations | 71 |
| $R^2$ | 0.054 |
| Adjusted $R^2$ | 0.040 |
| Residual Std. Error | 0.762 (df = 69) |
| F Statistic | 3.920 (df = 1; 69) |
| *Note:* | For illustration only without any statistical claim. **p<0.05; ***p<0.01 |

Table S5: Result of logistic regression of preeclampsia on expression level of *PHACTR2* controlling on level of *FAM46A*: (logit(PE) = $\alpha$+$\beta_1$PHACTR2+$\beta_2$FAM46A)

| | *Dependent variable:* |
|---|---|
| | PE |
| PHACTR2 | −1.230** |
| | (0.503) |
| FAM46A | −1.285*** |
| | (0.374) |
| Constant | 12.054*** |
| | (4.037) |
| Observations | 71 |
| Log Likelihood | −28.280 |
| Akaike Inf. Crit. | 62.560 |
| *Note:* | For illustration only without any statistical claim. **p<0.05; ***p<0.01 |

# S3 Supplementary Methods

## S3.1 Overview of Model-X knockoff filter

Barber and Candès (2015) introduced a novel framework, "knockoffs," for obtaining FDR control in feature selection, bypassing the standard calculation of p-values. The knockoff filtering procedure can be used as a wrapper by combining it with any feature selection method that generates feature importance measures that meet certain conditions. The basic principle of the knockoff procedure is to create a (or multiple) artificial "knockoff" data based on the original data, which is similar to the original real data, without using any information from the response variable. Then, the real data ($X$) and their knockoff copy data ($\widetilde{X}$) are run together into a model to obtain an importance measure for each original or knockoff variable so that the knockoff variables then function as negative controls for the original covariates. Each original variable's estimated importance measure is then compared to that of its corresponding knockoff variable, whose actual effect is known to be zero. Variables are chosen on the basis of this means of variable importance, which is low when the influence of the real variable cannot be distinguished from the estimated effect of a knockoff variable.

Table S6: Logistic regressions of preeclampsia on all selected variables or after removing colinear variables

| | *Dependent variable:* | | | |
|---|---|---|---|---|
| | PE | | | |
| | (1) | (2) | (3) | (4) |
| MBNL3 | −3.086 | | | −4.325*** |
| | (2.205) | | | (1.657) |
| PHACTR2 | −6.348** | −2.915*** | −4.551** | −2.574** |
| | (3.187) | (1.023) | (1.933) | (1.032) |
| FAM46A | −4.295 | −1.650** | −3.847** | −2.646*** |
| | (2.204) | (0.663) | (1.496) | (0.933) |
| GSE1 | 5.671 | 2.315*** | 3.907** | 3.355*** |
| | (3.070) | (0.834) | (1.813) | (1.299) |
| HEMGN | −3.130 | | −3.801*** | |
| | (1.886) | | (1.451) | |
| BPGM | −0.854 | −2.762*** | | |
| | (1.558) | (0.908) | | |
| MAFK | 1.847 | 2.693*** | 1.406 | 1.270 |
| | (1.456) | (1.043) | (0.912) | (0.869) |
| Constant | 60.776 | 11.349 | 38.539 | 28.820 |
| | (31.972) | (8.748) | (20.500) | (15.911) |
| Observations | 71 | 71 | 71 | 71 |
| Log Likelihood | −7.850 | −12.200 | −11.797 | −10.958 |
| Akaike Inf. Crit. | 31.700 | 36.400 | 35.595 | 33.916 |
| *Note:* | For illustration only without any statistical claim. **p<0.05; ***p<0.01 | | | |

Knockoff filtering aims to select 'relevant' variables that are conditionally dependent on the response variable given all the other covariates, i.e., a subset of $X$ that affects $Y$. $X_j$ is referred to as 'null' if it is conditionally independent of the response variable $Y$ once the other $p-1$ variables are given.

Knockoff filtering for the selection of important variables consists of three main steps:

**Step 1: Knockoff variable construction.** When $n \leq p$, the knockoff variables $\widetilde{X}$ can be generated as Model-X knockoffs, which are defined as a new set of random variable $\widetilde{X}_j, j = 1, \cdots, p$ that satisfies the properties:
1) for any subset $S \subset \{1, \cdots, p\}$,

$$(X, \widetilde{X})_{\text{swap}(S)} \overset{d}{=} (X, \widetilde{X}), \tag{1}$$

where $(X, \widetilde{X})_{\text{swap}(S)}$ is obtained by swapping the $X_j$ and $\widetilde{X}_j$ for each $j \in S$;
2) $\widetilde{X} \perp\!\!\!\perp Y|X$ (guaranteed if $\widetilde{X}$ is constructed without looking at $Y$).

Existing approaches to construct valid model-X knockoff will be discussed in the next section S3.2.

**Step 2: Calculate the important statistics.** Once the knockoff variables are generated, the model for variable selection is run on an augmented data set with response $y$ and $2p$ many features $X_1, ..., X_p$, $\widetilde{X}_1, ..., \widetilde{X}_p$. Then, a pairwise statistics $W_j$ for each $j \in \{1, ..., p\}$ is computed. This statistic depends on the response, original variables, and knockoffs. A valid statistic should have *flip-sign property* that swapping the $j$th variable with its knockoff has the effect of changing the sign of $W_j$ (Candès et al., 2018). A large positive value of $W_j$ provides some evidence that the distribution of Y depends upon $X_j$, whereas under the null (for those null/noise variables), $W_j$ has a symmetric distribution and, therefore, is equally likely to take on positive and negative values. For example, a common option for valid $W_j$ can be the lasso coefficient difference (LCD) (with the value of $\lambda$ can be decided by cross-validation).

**Step 3: Find the threshold and select variables.** Based on the property of pairwise exchangeability (see Candès et al. (2018) Section 3.2) where the $W_j$ for noise variables are equally likely to be positive or negative (distributed symmetrically to zero, i.e. $\#\{null\ j : W_j \leq -t\} \overset{d}{=} \#\{null\ j : W_j \geq t\}$), the set of important features with an FDR at a pre-specified level $q \in [0, 1]$ is selected as $\hat{S} = \{j : W_j \geq t\}$ with the threshold $t = T$, defined as:

$$T = \min\left\{t > 0 : \frac{\#\{j : W_j \leq -t\}}{\#\{j : W_j \geq t\} \vee 1} \leq q\right\}.$$

This formulation relies on the symmetry of the null feature statistics around zero to estimate the false discoveries. Figure S22 illustrates how the threshold $t$ finds the feature space into regions that used as the numerator and denominator of the FDP estimator. For each threshold $t$, the corresponding false discovery proportion (FDP) of set $\hat{S}$ can be controlled, as follows:

$$\text{FDP}(t) \approx \frac{\#\{j \in \mathcal{H}_0 : W_j \geq t\}}{\#\{j : W_j \geq t\}} \approx \frac{\#\{j \in \mathcal{H}_0 : W_j \leq -t\}}{\#\{j : W_j \geq t\}} \leq \frac{\#\{j : W_j \leq -t\}}{\#\{j : W_j \geq t\}} \leq q,$$

and the procedure of selecting controls the modified FDR defined as

$$\text{mFDR} = \mathbb{E}\left[\frac{\#\{j : j \in \hat{S} \cap \mathcal{H}_0\}}{|\hat{S}| + 1/q}\right].$$

Slightly more conservatively, given by incrementing the number of negatives in discoveries by 1, knockoff+ defines the threshold as

$$T_+ = \min\left\{t > 0 : \frac{1 + \#\{j : W_j \leq -t\}}{\#\{j : W_j \geq t\} \vee 1} \leq q\right\}.$$

Selecting $\hat{S} = \{j : W_j \geq T_+\}$ controls the usual FDR as,

$$\text{FDR} = \mathbb{E}\left[\frac{\#\{j : j \in \hat{S} \cap \mathcal{H}_0\}}{|\hat{S}|}\right].$$

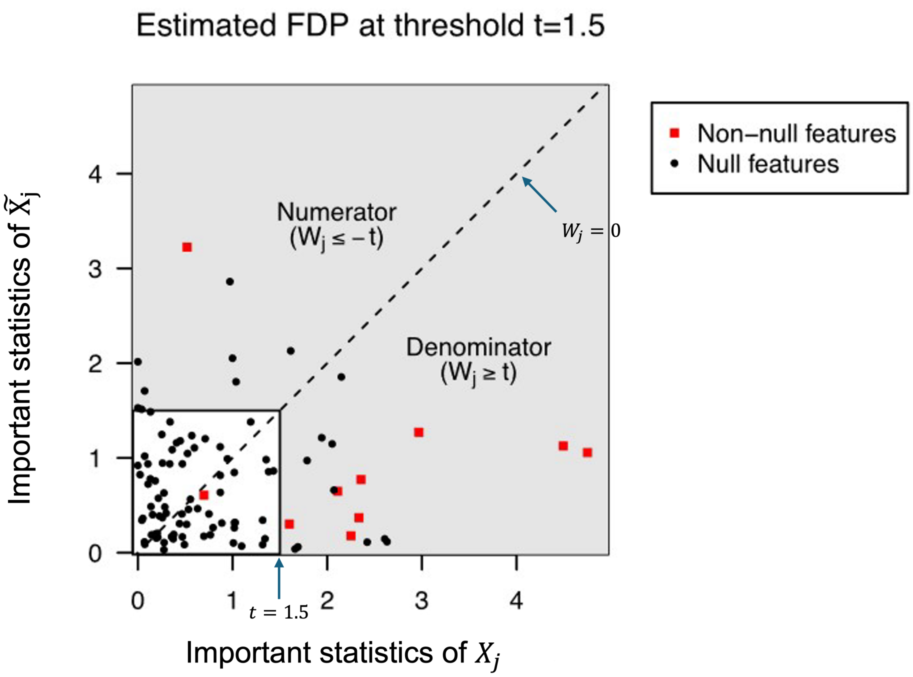


Figure S22: Illustration of the estimated false discovery proportion (FDP) at threshold $t = 1.5$. Each point corresponds to a feature, where red squares represent non-null features and black circles represent null features. The $x$-axis shows the importance statistic (e.g., coefficient from lasso) of the original variable $X_j$, and the $y$-axis shows that of the corresponding knockoff $\widetilde{X}_j$. The grey regions highlight the areas used in FDP estimation: the "Numerator" region ($W_j \leq -t$) and the "Denominator" region ($W_j \geq t$). Under the null, $W_j$ is symmetric about zero; thus, the number of null features in the numerator can be used to estimate the number of false discoveries among selected features in the denominator. For each threshold $T = t$, an estimated $\text{FDP}(t)$ is computed as the ratio of the number of features in the numerator to those in the denominator. To control the FDR at a target level $q$, the procedure selects the minimum threshold $t$ such that $\text{FDP}(t) \leq q$. For a more conservative guarantee, knockoff+ adds 1 to the numerator, resulting in a threshold $T_+$ that ensures strict FDR control. (Adapted from https://web.stanford.edu/group/candes/knockoffs/index.html)

**Algorithm S1** Sequential Conditional Independent Pairs (SCIP), Candès et al. (2018)

$j = 1$
**while** $j \leq p$ **do**
  Sample $\widetilde{X}_j$ from $\mathcal{L}(X_j | X_{-j}, \widetilde{X}_{1:j-1})$, conditionally independently from $X_j$
  $j = j + 1$
**end while**

## S3.2 Review of existing knockoff generators

Table S7: Existing knockoff generating method for benchmark in this paper

| Type | Subtype | Details | Limitations | Type limitation | In Benchmark |
|---|---|---|---|---|---|
| Second-order Approximation | SDP | oracle (simulation), JS-shrunk | Low power | Fail when $P_X$ contain higher orders; Affected by covarince estimation | T |
| | MVR | | Computation is slow in R | | T |
| | Entropy Knockoff (ME) | | Computation is slow in R | | F |
| | Deep Knockoff | | Large sample size required | Large sample size required | F |
| SCIP (Approximate) | KnockoffScreen | K-nearest neighbour; linear regression | OLS is not often valid; K is too small in high-dimension | Trade-off in speed and accuracy | F |
| | SeqKnockoff | lasso or elastic net regression | Computationally very slow | | T |
| | PCKO | PC-regression | Computationally slow | | T |
| Structured correlation | IPAD | | Assume X follows factor model | Might fail when X not follow factor model | T |

There are many possible ways to construct valid knockoff variables. Although it is theoretically ensured that knockoff variables with model-X properties controlled FDR, the power of knockoff filtering varies among different construction

approaches. An ideal knockoff construction method can identify as many variables as possible (i.e. maximum power) while controlling the FDR under the desired level. Two main groups of knockoff-generating approaches are described below (Summarised in Table S7).

### S3.2.1 Joint distribution approximation knockoff generators

The first type of knockoff variable generators are based on the approximation of the property 1 of model-X. For example, $\widetilde{X}$ is a second-order knockoff copy when the first two orders of joint distribution $(X, \widetilde{X})$ and $(X, \widetilde{X})_{swap(S)}$ are matched. This condition is equivalent to $\mathbb{E}(X) = \mathbb{E}(\widetilde{X})$ and

$$\mathrm{cov}(X, \widetilde{X}) = \begin{bmatrix} \Sigma & \Sigma - \mathrm{diag}\{s\} \\ \Sigma - \mathrm{diag}\{s\} & \Sigma \end{bmatrix}, \tag{2}$$

where $\Sigma$ is the covariance matrix of $X$ and $s \in \mathbb{R}^p$ such that 2 is positive semidefinite. Then, to improve the power of second-order approximation knockoffs, different algorithms might be applied to solve $s$. In the original model-X paper (Candès et al., 2018), semidefinite programming (SDP) tried to maximise the power by minimising the marginal correlation between the knockoff variable and its original variable. However, this method may show very low power in some settings, such as high-correlated or high-dimension settings. This can be observed in our simulation studies (e.g. Figure S3). Minimised reconstructability (MRC) knockoff (Spector and Janson, 2022) proposed another $s$-solving algorithm that maximises the conditional variance of variables conditioning on the other variables and knockoff variables or maximises the entropy of the joint distribution of $X$ and $\widetilde{X}$. Overall, second-order approximation knockoff construction requires mean and covariance of $X$ known or estimated and is the exact construction for $X$ following multivariate Gaussian and second-order approximation for other distributions. When the covariate distribution does not follow multivariate Gaussian, the second-order approximation knockoff might fail to control FDR, especially when the relationship among covariate $X$ is non-linear and cannot be fully described by the first two orders. This limitation is shown in the simulation Section 3 and Figure 2.

Deep knockoffs (Romano et al., 2020) can generate knockoff variables also based on the joint distribution approximation but with higher-order matching. Similar to other deep learning models, this knockoff construction method also requires a very large sample size to train.

### S3.2.2 SCIP knockoff generators

A general algorithm for the exact construction of the knockoff variable has been proposed for any type of $X$ (Candès et al., 2018), namely sequential conditional independent pairs (SCIP). The SCIP algorithm (Algorithm SS1) is based on the derived property of model-X knockoffs that any pair $(X_j, \widetilde{X}_j)$ is exchangeable conditioning on all other variables and their knockoffs so that for each $j \in (1, \cdots, p)$, $\widetilde{X}_j$ can be generated by sampling from $\mathcal{L}(X_j | X_{-j}, \widetilde{X}_{1:j-1})$. However, implementing this algorithm is complicated or not always practical, since the conditional distribution has to be recomputed at each step. Exact sampling methods have been implemented for Gaussian distributions or distributions where the latent variables can be represented as a Bayesian network (BN), such as Sesia et al. (2019) for discrete and hidden Markov chain models with application in GWAS and Gimenez et al. (2019) for Gaussian mixture models. This proposed knockoff sampling algorithm relaxes the conditional distribution $\mathcal{L}(X_j | X_{-j}, \widetilde{X}_{1:j-1})$ to only condition on a subset, that is, neighbours of $X_j$ on the Bayesian network, instead of all other variables and constructed knockoffs, thus reducing the computational burden.

In addition to exact SCIP sampling (e.g., for the Markov chain model in GWAS), several approximate SCIP-based methods have been proposed and applied in more general biological studies without any distribution assumption on $X$. Generally, these approximate sampling methods use different approaches to approximate the conditional distribution, i.e., $\mathcal{L}(X_j | X_{-j}, \widetilde{X}_{1:j-1})$, sequentially for each variable. For example, Sequential Knockoffs, proposed by (Kormaksson et al., 2021), use prediction residuals from lasso or elastic net (EN) as the conditional distribution. `KnockoffScreen` for GWAS (He et al., 2021) used linear regression with the most correlated variables as predictors. Jiang et al. (2021) used principal component (PC) regression in their knockoff boosted tree (`KOBT`) model to sequentially fit the dependence relationship in each variable with the other $j - 1$ variables. These approximate SCIP-based knockoff construction methods showed empirically valid in most of the cases provided in their literature. However, there are limitations to their applications in more challenging cases, such as high-dimension studies. Sequential knockoffs can be computationally slow when $X$ consists of hundreds of variables, since lasso and EN are iteratively fitted with cross-validation for every variable in the data set; OLS in `KnockoffScreen` is not always feasible because of the singularity when $X$ is high-dimensional or high-correlated; PC-regression-based SCIP knockoff generation method, `KOBT`, might be not able to capture the dependence among variables sufficiently with a low number of PCs (leading to inflated FDRs) or might capture redundant variance with a large number of PCs (leading to low powers) since PCs in PC regression are the

eigenvectors with the largest eigenvalues that capture most of the total variance in the other $j-1$ variables instead of capturing the variance that can explain the most of variance of $X_j$.

### S3.2.3 IPAD

Intertwined probabilistic factors decoupling (IPAD) (Fan et al., 2020) assumes $X$ follows the exact factor model, i.e., $X = X = TP' + E = C + E$, and estimate parameters ($\theta$) of $C$ and $E$. Then the knockoffs can be construct as $\widetilde{X}(\hat{\theta}) = \hat{C} + \widetilde{E}$, where $\widetilde{E}$ is independently sampled from estimated distribution of $E$. In the IPAD paper, the low-rank structure $C$ is estimated by SVD and the residual $E$ is estimated as an i.i.d. centred normal distribution with variance equal to the empirical variance of the residual.

## S3.3 PLSKO

Here we proposed a new knockoff variable construction method, 'PLSKO', aiming to control the FDR effectively in high-dimensional biological data and meanwhile to reach as high power as possible. PLSKO is an approximate sequential conditional independent pairs (SCIP) approach Candès et al. (2018) (described in Algorithm S1 and Figure 1).

The PLSKO procedure to generate knockoff variable $\widetilde{X}$ for a dataset $X \in \mathbb{R}^{n\times p}$ is described below (also see Algorithm S2):

- *Step 1: Generating neighbour set for each variable $X_j, j = 1, \ldots, p$.*
  To lower the computational burden and inspired by Gimenez et al. (2019), Sesia et al. (2019) and He et al. (2021), the conditioning distribution $\mathcal{L}(X_j|X_{-j}, \widetilde{X}_{1:j-1})$ can then go down to $\mathcal{L}(X_j|X_{k\in BN_j}, \widetilde{X}_{1\leq k\leq j-1,k\in BN_j})$, where $BN_j$ is the neighbours of $X_j$ in the Bayesian network of $X$. Instead of controlling all the other variables ($X_{-j}$) and knockoff variables before ($\widetilde{X}_{1:j-1}$), now the conditional distribution is only on those in the neighbourhood of $X_j$, and other variables are assumed to be independent with $X_j$. When there is no user-prespecified neighbour list, PLSKO uses the sample correlation with $X_j$ as the similarity measure and defines the nearest variables either with a correlation greater than a user-defined absolute correlation threshold or with a correlation in the top quantile of all pairwise correlations in the sample. The choice of absolute or quantile threshold is to ensure that $P(X_j|X_{k\in BN_j}, \widetilde{X}_{1\leq k\leq j-1,k\in BN_j})$ accurately mimics $P(X_j|X_{-j}, \widetilde{X}_{1:j-1})$ and to avoid overfitting.

- *Step 2: Fit $X_j$ as a function of $X_{k\in BN_j}$ and $\widetilde{X}_{1\leq k\leq j-1,k\in BN_j}$.*
  To generate knockoff variables from $\mathcal{L}(X_j|X_{k\in BN_j}, \widetilde{X}_{1\leq k\leq j-1,k\in BN_j})$, we assume a model,
  $$X_j = g(X_{k\in BN_j}, \widetilde{X}_{1\leq k\leq j-1,k\in BN_j}) + \epsilon_j,$$
  where $\epsilon_j$ is a random error term. In other words, $g(\cdot)$ is the component of the information in $X_j$ that can be explained/represented by the other variables and constructed knockoff variables; $\epsilon_j$ is the unique and irrepretentalbe information in $X_j$. PLSKO approximates $g(\cdot)$ as a linear model, that is, $g(X_{ij}|X_{k\in BN_j}, \widetilde{X}_{1\leq k\leq j-1,k\in BN_j}) = \alpha + \sum_{k\neq j,k\in BN_j} \beta_k X_{ik} + \sum_{k\leq j-1,k\in BN_j} \gamma_k \widetilde{X}_{jk}$, and uses partial least squares regression (PLS regression, described in Appendix section S3.3.4) to estimate parameters in model $g(\cdot)$ and calculate the fitted value of $X_j$, namely $\hat{X}_j$.

- *Step 3: Generate knockoffs by permuting the residuals within samples.*
  Residuals are then calculated as $\hat{\epsilon_j} = X_j - \hat{X}_j$, as an approximation of the conditional distribution $\mathcal{L}(X_j|X_{-j}, \widetilde{X}_{1:j-1})$, representing the part of $X_j$ that cannot be predicted by the other variables and the generated knockoff variables. Intuitively, we see the predictable part, $\hat{X}_j$, as the part that needs to be controlled and the part that preserves the dependency relationship among the variables, thus is to be preserved in knockoff variables as well. We see the residual as the unpresentable unique information of $X_j$, independent with the residuals of other variables. Only if the residual is correlated with the outcome, we could say (but not always without other conditions) $X_j$ might be important as it is not independent of the outcome $y$ conditioning other variables. In knockoff construction in PLSKO, we generate knockoff variable $\widetilde{X_j}$ by adding up $\hat{X}_j$ and the permuted residual $\widetilde{\epsilon_j}$. Permutation of residuals instead of sampling from a specified and parameterised conditional distribution avoids more assumptions and allows the PLSKO to be applied on data from more types of distribution.

**Algorithm S2** Partial Least Square Knockoff (PLSKO)

**Require:** Dataset $X \in \mathbb{R}^{n\times p}$; Neighbour set for each $X_j$: $BN_j,\ j = 1, \dots, p$
Number of components $r$ in PLS regression
Sparsity applied to sparse PLS regression $s$

$j = 1$
Centered $X$ with $X - \mu$, $\mu \in \mathbb{R}^p$ is the sample mean of $X$

**while** $j \leq p$ **do**
Define the neighbour knockoff set of $X_j$: $BN_{ko,j} = \{k | k \in BN_j \text{ and } k \leq j-1\}$

**if** $|BN(X_j)| = 0$ **then**
$\hat{X}_j \leftarrow 0$
**else if** $|BN(X_j)| = 1$ **then**
Fit OLS regression as $y_{OLS} = X_j$, $X_{OLS} = [X_{k\in BN_j}, \widetilde{X}_{BN_{ko,j}}]$ (augmented matrix)
Calculate $\hat{X}_j$
**else**
Fit PLS regression as $Y_{PLS} = X_j$, $X_{PLS} = [X_{k\in BN_j}, \widetilde{X}_{BN_{ko,j}}]$ (augmented matrix), with $r$ components;
Calculate $\hat{X}_j = \hat{X}_{j,PLSreg}$ on the first $r$ components
**end if**

Get $\tilde{\epsilon}_j$ by permuting the residual $\hat{\epsilon} = X_j - \hat{X}_j$;
Calculate $\widetilde{X}_j = \hat{X}_j + \tilde{\epsilon}_j$;
$j = j + 1$
**end while**

$\widetilde{X} = \widetilde{X} + \mu$
**Output:** $\widetilde{X}$

### S3.3.1 PLSKO Tuning

As listed in Section 2.2, three parameters in PLSKO are required to be specified, including the number of PLS components (ncomp), the threshold for defining variable neighbourhoods (threshold.abs or threshold.q), and the sparsity level (sparsity). These parameters influence the trade-off between power, false discovery rate (FDR) control, and computational cost. To optimise the performance of the PLSKO knockoff generator, we developed a semi-simulation-based parameter tuning framework. The tuning procedure relies on semi-simulation, wherein response variables are synthetically generated using real omics data and known true coefficients. For each parameter setting, knockoff variables are generated using the PLSKO function, and variable selection is performed to estimate empirical FDR and power by comparing selected variables with the known ground truth. the optimal configuration is selected as the one yielding the lowest mean or median FDP according to the specified measure. Among tied configurations, the one with the highest threshold.abs, lowest ncomp, and lowest sparsity will be selected as the optimal by default. More details can be found in the tuning function vignette (`https://guannan-yang.github.io/PLSKO/PLSKO_tune.html`). Supplemental Figure S18-S21 are shown as the output examples.

### S3.3.2 Computational complexity of PLSKO

As shown in the Algorithm S2 (PLSKO), PLSKO generates knockoff variables for a given $X \in \mathbb{R}^{n\times p}$ by sequentially fitting $p$ PLS regressions with number of components $r$. In our implementation, we applied the Non-linear iterative partial least-squares (NIPALS) algorithm to fit the PLS regression. The complexity of NIPALS is $O(npr)$, and the complexity of generating the knockoff variables by prediction is $O(np)$, resulting in a total complexity of approximately $O(npr)$ per knockoff variable at most. Therefore, the overall complexity of PLSKO is at most $O(np^2r)$, when no neighbour variable filtering is applied at the first step of PLSKO. When applying neighbour filtering, for example, defining the 50% most correlated variables as neighbours, the number of variables to be fitted in each regression can be reduced to $p/2$, effectively halving the computational cost. In practice, we found that the neighbour filtering can significantly improve the computational efficiency of PLSKO, especially when the number of variables is large.

Instances of computation times of PLSKO on real datasets with varying variable sizes are shown in Supplemental Figure S11a, S12, S14, S15. Additionally, we implemented PLSKO with SIMPLS algorithm (See Supplemental Section S3.3.4), which is slightly faster than NIPALS (Supplemental Figure S14, S15). We found that the computational time of PLSKO-SIMPLS was about 2/3 of PLSKO-NIPALS, which is aligned with the theoretical complexity: NIPALS has $3O(np)$ in the inner-loop of each component, whereas SIMPLS directly calculates scores and loadings in $2O(np)$.

In summary, the computational complexity of PLSKO $O(np^2r)$ can be further reduced via neighbour filtering. Implementing PLSKO with SIMPLS algorithm can further reduce the computational time to about 2/3 of PLSKO with NIPALS.

### S3.3.3 PLS-AKO

In case studies, we generated multiple knockoff variables for each observed fixed dataset and incorportated by aggragation of multiple knockoff (AKO) (Nguyen et al., 2020) to accommodate for the randomness and improve the stability from knockoff variable generation. To distinguish between PLSKO with a single run and multiple PLSKO incorportated by AKO, we refer the later one as 'PLS-AKO'. The PLS-AKO used in this study is briefly described as the following steps:

- **Step 1: Construct multiple knockoff by PLSKO.** $B$ knockoff variables are constructed by PLSKO parallel as $\widetilde{X^{(b)}} \in \mathbb{R}^{n\times p}$, $b = 1, \dots, B$.

- **Step 2: Calculate the importance statistics.** $X$ and each constructed knockoff variable set $\widetilde{X^{(b)}}$ are run into the (logistic) lasso regression, and the LCD is used as the importance statistics $W^{(b)} \in \mathbb{R}^p, b = 1, \dots, B$.

- **Step 3: Calculate intermediate p-value.** For each $W^{(b)}$, calculate the empirical $p-$value for $X_j, j = 1, \dots, p$ as:

$$\pi_j^{(b)} = \begin{cases} \frac{1+\#\{k: W_k \leq -W_j\}}{p} & \text{if } W_j > 0 \\ 1 & \text{if } W_j \leq 0 \end{cases} \tag{3}$$

- **Step 4: Aggregate empirical $p-$value.** For each $X_j$, $B$ empirical $p-$values, $\pi_j^{(b)}$, are aggregated by the quantile aggregation procedure as:

$$\tilde{\pi}_j = \min\left\{1, \frac{q_\gamma\left(\{\pi_j^{(b)} : b \in [B]\}\right)}{\gamma}\right\} \tag{4}$$

where $q_\gamma(\cdot)$ is the $\gamma$-quantile function. As tested and recommended in Nguyen et al. (2020), we fix $\gamma = 0.3$, and $B = 50$ as 50 knockoff variables has been generated.

- **Step 5: Adjust $p-$value to control the FDR.** With $\tilde{\pi} \in \mathbb{R}^p$, the Benjamini-Hochberg (BH) procedure is used to control the FDR. Variables with the $\tilde{\pi}$ less or equal than the BH threshold are selected by PLS-AKO.

In practice, we found that PLS-AKO generally had lower FDR and higher power compared to the average of PLSKO before aggregation.

#### S3.3.4 Review of Partial Least Square (PLS) regression

Partial Least Squares (PLS) (Wold, 1966) is a wide class of techniques for modelling relations between sets of observed variables by means of latent variables, assuming that observed data, including predictors and multiple responses, are all driven by a small number of latent variables and the latent variables from the two datasets are highly-related. PLS has been widely applied to high-dimensional biological data for a variety of research questions such as classification and biomarker identification, due to the ability of PLS to work very well for data with very small sample sizes and a large number of parameters and high computational and statistical efficiency (Boulesteix and Strimmer, 2007).

The general underlying model of multivariate PLS is: consider predictor $X \in \mathbb{R}^{n\times p}$ and response $Y \in \mathbb{R}^{n\times q}$, we assume both of them follow the factor model, that is:

$$\begin{aligned} X &= TP^\top + E \\ Y &= UQ^\top + F, \end{aligned} \tag{5}$$

where the $T$and $U \in \mathbb{R}^{n\times r}$ are matrices of the $r$ extracted score vectors (components, latent factors), the matrix $P \in \mathbb{R}^{p\times r}$ and $Q \in \mathbb{R}^{q\times r}$ are the matrices of loadings, and $E \in \mathbb{R}^{n\times p}$ and $F \in \mathbb{R}^{n\times q}$ are the matrices of residuals, assumed to be independent and identically distributed random normal variables. The decompositions of $X$ and $Y$ are made so as to maximise the covariance between $T$ and $U$, i.e., iteratively find vectors $w, c$, such that $\arg\max_{\|w\|=1,\|c\|=1} w^\top X^\top Y^\top c$, and then $t = Xw, u = Yc$.

PLS is an iterative process. After the extraction of the score vectors $t$, $u$ the matrices $X$ and $Y$ are deflated by subtracting their rank-one approximations based on $t$ and $u$. Different forms of deflation define several variants of PLS (Rosipal and Krämer, 2006). For example, canonical PLS (Mode A) deflates both $X$ and $Y$ in each iteration. The relation between the two blocks is symmetric in this mode, and it is more appropriate for modelling existing relations between sets of variables (e.g., investigating relationships between RNA and proteins) in contrast to prediction purposes. In contrast, PLS regression (Höskuldsson, 1988) assumes $T = \{t_i\}_{i=1}^r$ (the latent variables of $X$) are good predictors of $U$ (the latent variables of $Y$). Deflation in regression mode is to remove a component of the regression of $Y$ on $t$ (the latent variables of $X$, instead of $u$) from $Y$ at each iteration.

**PLS regression** To introduce our new PLSKO method, we briefly describe the steps of PLS regression and prediction. When predicting $Y$ from $X$, PLS regression assumes that $T$ are good predictors of $U$, i.e., $U = TD + H$, where $D \in \mathbb{R}^{p\times p}$ is a diagonal matrix, so the equation 5 can be written as:

$$Y = UQ^\top + F = (TD + H)Q^\top + F = TDQ^\top + (HQ^\top + F) = TC^\top + F^*, \tag{6}$$

where $C^\top = DQ^\top \in \mathbb{R}^{n\times q}$ is the regression coefficients and $F^* = HQ^\top + F$ is the residual matrix. Again, from equation 5, we have $T = XW(P^\top W)^{-1}$, so when fitting $Y = XB + F^*$, the regression coefficients $B = W(P^\top W)^{-1}C^\top = X^\top U(T^\top XX^\top U)^{-1}T^\top Y$. For a new set of $X_{new}$,

$$\hat{Y}_{PLSreg} = X_{new}\hat{B} = X_{new}X^\top U(T^\top XX^\top U)^{-1}T^\top Y. \tag{7}$$

In this paper, PLS regression is calculated by Non-linear iterative partial least-squares (NIPALS) algorithm (Wold, 1975) by defalt. PLSKO-SIMPLS is implemented using The Statistically Inspired Modification of Partial Least Squares (SIMPLS) algorithm to fit PLS regression, which can be computationally faster than the iterative approach of NIPALS (De Jong, 1993).

**Validity and advantages of PLS regression for SCIP** In high-dimensional data, where $n < p$, or in highly correlated data, where the rank of the data $r < p$, the usual linear regression with ordinary least squares (OLS) cannot be applied, since the covariance matrix of $X$ (which can have a maximum rank $n-1$) is singular. That is, OLS cannot be applied to conditional distribution calculation in the SCIP algorithm when neighbour variables and their knockoffs outnumber the sample size. Otherwise, reducing the neighbour numbers lower than the sample size by placing

a sparser neighbour filter might lead to incorrect conditional distribution calculation due to insufficient covariates, then the violation of the exchangability requirement of the model-X knockoff, and thus inflated FDR. In contrast, PLS regression can be applied to cases in which $n < p$ and $r < p$ without reducing the number of predictors. When using PLS regression to fit the original variable with a number of components no less than the true number, the prediction error converges to 0 at a rate of root-$n$, even when $n << p$ (**?**). This property does not require the normality of data as an essential assumption. Therefore, the knockoff variable generated by PLSKO and the original variables are asymptotically exchangeable in the number of samples $n$. In turn, the property 1 in model-X knockoff (Supplemental Section S3.1) holds.
Principal component regression (PC regression) is also a reduced rank regression method, which firstly extracts the first components (called principal components) that capture most of the total variance and then uses the first principal components in the data as the predictor variables in the regression. Principal component extraction in PC regression does not use the response for component construction. In contrast, PLS regression takes the response variable $y$ into account for the construction of the components. This feature allows PLS regression to capture more information in the response variable $y$ with a lower number of components than PC regression and better performance in prediction problems. Moreover, in PLS regression, dimension reduction and regression are performed simultaneously. For example, consider a scenario where $y$ is a univariate response variable and $X$ is a predictor matrix with a rank of 3. PC regression would require computing at least 3 components to fully represent the predictor space. In contrast, PLS regression can fit the model using just 1 component, which is a linear combination of the three latent factors that maximizes the covariance with $y$. As the SCIP algorithm is iterative on each variable in the dataset, the computational efficiency for conditional distribution is high and therefore quite advantageous for high-dimensional data. The lower number of components and the simultaneous fitting make PLS regression more efficient than PC regression. In summary, the use of PLS regression in the computation of the conditional distribution ensures accuracy with enhanced efficiency.

### S3.4 Benchmark methods

In the simulation experiments in this study, second-order approximation (SOA) knockoff generators for benchmark include: (they may categorised by the algorithms for solving $s$ and the covariance estimators)

- *oracle*: Semidefinite programming (SDP) with the known oracle covariance matrix, implemented by the `R` package `knockoff`;
- *JS*: SDP with the covariance estimated by James-Stein-type Shrinkage (JS) (Ledoit and Wolf, 2003) (JS), implemented by the `R` package `knockoff` and `corpcor`;
- *mvr-oracle*: Minimised the variances-based reconstructability (MVR) algorithm with the oracle covariance, implemented by the `Julia` package `Knockoffs`;
- *mvr-JS*:MVR with the JS-type estimated covariance, implemented by the `Julia` package `Knockoffs`

In the simulation experiments in this study, SCIP knockoff generators for benchmark include:

- *PC*/*PCKO* (in simulation): SCIP with prior neighbourhood selection and with PC regression as the conditional distribution approximation. Same parameter settings (i.e., number of components and neighbour threshold) used with PLSKO;
- *PCKO*(in semi-simulation): SCIP without neighbour selection and 'full' components, with PC regression as the conditional distribution approximation, implemented by the `R` package `KOBT`. Same parameter setting with PLSKO-full.
- *seqko*: SCIP with lasso or elastic net as the conditional distribution approximation, implemented by the `R` package `seqknockoff` (https://github.com/kormama1/seqknockoff).

In this paper, the numbers of components used in IPAD were the true rank of simulation data, or determined by the $PC_{p1}$ criterion in the semi-simulation study, same as PLSKO-full.
Other non-knockoff methods for benchmarking (results in Supplemental Figures) include:

- Lasso: The regularisation parameter $\lambda$ is gained via cross-validation to minimise the prediction error.
- ElasticNet: The mixing parameter is fixed as $\alpha = 0.5$ and $\lambda$ is gained via cross-validation with the minimum error.
- limma: Linear models were fitted to log-transformed expression data, and empirical Bayes moderation was applied to obtain moderated t-statistics. P-values were adjusted for multiple testing using the Benjamini–Hochberg (BH) method to control the false discovery rate.
- Wilcoxon test: A Wilcoxon rank-sum test was performed. The BH method was used to adjust p-values.

## S3.5 Simulation studies

Below we describe in detail the simulation of a single data set $(X, y)$ for a given parameter configuration. For each parameter configuration we simulate $n_{sim} = 100$ data sets and apply the knockoff filtering with different knockoff variable generating methods.

### S3.5.1 Data generation

- **Simulation of $X$.** We simulate the $n \times p$ design matrix $X$ from a block factor models. Each block of $X$ is generated from $X_b = F_b\Lambda_b' + E_b$, where latent factors $F_b \in \mathbb{R}^{n\times r_b}$ the factor loadings $\Lambda_b \in \mathbb{R}^{p_b\times r_b}$, and $E \in \mathbb{R}^{n\times p_b}$ $\sim \mathcal{N}(0, I_{p_b})$. The correlation structure of $X$ from the same block is represented by the low-rank factor component $F_b\Lambda_b'$ and is expected to be controlled in the knockoff filter. Variables from different blocks are independent. We set 5 blocks and the number of variables in each block as $p_b = 100$ and the number of latent factors as $r_b = 3$. Factor loadings are sampled independently from a uniform distribution $U(1, 2)$ with a random sign of equal probability.
- **Simulation of $X$ for robustness tests.** We simulate $X$ from a block multivariate Gaussian and block quadratic factor model. The blocks of multivariate normal $X$ are generated from $\mathcal{N}(\mathbf{0}, \mathbf{\Sigma_{p_b}})$, with $\Sigma = (\sigma_{ij})$, $\sigma_{ij} = \rho^{|i-j|}$ (autoregressive process of order one, AR1) or $\sigma_{ij} = \rho$ (equicorrelation setting). $\rho$ is set to be 0.5. In each block of the quadratic factor model, half of the $p_b$ variables are generated from the factor model (as described above) and the other half of the $p_b$ variables are generated as the square of the variables from the generated half. This quadratic model provides a scenario in which the dependency among variables is non-linear, i.e., $X$ is not sufficiently described by the first two moments.
- **Simulation of $y|X$.** The response variable $y$ is simulated from $y_i = f(x_i) + (c \times s \times p)\epsilon_i$. $c$ is a constant controlling the noise-to-signal ratio, $s \times p$ is the number of true signal variables, and *epsilon* is the model error following $\mathcal{N}(0, 1)$. $X$ is rescaled column-wise before generating $y$. Without further specification, $c = 0$ (zero noise in $y$ generation) and $s = 0.1$ (10% of variables are important). For continuous $y$, $f(x) = x\beta$, where $\beta$ is the coefficients sampled from $U(3, 5)$ with a sign following Bin(0.5) for the signal variables and $\beta = 0$ for the null variables; for the categorical $y$, $f(x) = \text{Bin}(\pi(x\beta))$, where $\pi$ is the Sigmoid function.

### S3.5.2 Simulations setting

- **Varying sample size** In this experiment we vary the sample size $n \in \{150, 160, \ldots, 250\}$ and compare the FDP and TPP across the benchmark methods.
- **Varying sparsity** We vary the sparsity $s \in \{0.1, 0.2, 0.3, 0.4\}$ (i.e., 50, 100, 150, 200 important variables are used to generate $y$) with fixed sample size $n = 500$.
- **Varying signal-noise ratio** We vary the signal-noise ratio $c \in \{1, 2, \ldots, 5\}$, corresponding to the approximate ratio of unmeasured important variables to the measured important variables $1 : 16, 1 : 8, \ldots, 5 : 16$ with fixed sample size $n = 500$.
- **Robustness test with varying sample size** We vary the sample size $n \in \{150, 160, \ldots, 250\}$ in two multivariate gaussian design $X$ and quadratic factor model to examine the robustness of PLSKO and other methods in different data distribution.
- **Binary response variable $y$** We generate the binary response $y$ by applying the sigmoid function on the centred linear combination of randomly selected important variables, and test on varying the sample size from 200 to 1000. Due to the near-zero power when controlling on the usual FDR (with $T_+$ threshold), we only control the modified FDR.

A summary is available in Table S8.

### S3.5.3 Performance measures

We use the false discovery proportion (FDP) and the true positive proportion (TPP) as the measure of performance for the knockoff generators, which are defined as:

$$\text{FDP} = \frac{V}{R} \tag{8}$$

where $V$ is the number of false positives in the selected variable set and $R$ is the total number of rejected hypotheses (selected variables), and

Table S8: Summary of configurations in simulation experiments

| Experiment | $X$ | | | | | | $y$ | | | Report in |
|---|---|---|---|---|---|---|---|---|---|---|
| | Distribtution | Number of latent factor per block, $r$ | Correlation, $\rho$ | Sample size, $n$ | Blocks, $b$ | Variables in each block, $p_b$ | Type | Sparsity, $s$ | Noise, $c$ | |
| Varying sample size | Factor model | 3 | NA | 150-250 | | | linear | 0.1 | 0 | Figure S3 |
| Varying sparity | Factor model | 3 | NA | 500 | | | linear | 0.1 - 0.4 | 0 | Figure S4 |
| Varying sigal-noise ratio | Factor model | 3 | NA | 500 | 5 | 100 | linear | 0.1 | 1:16- 5:16 | Figure S4 |
| Robustness test | Multivariate Gaussian: AR1 and Equicorr | NA | 0.5 | 150-250 | | | linear | 0.1 | 0 | Figure S5 |
| Robustness test | Quadratic factor model | 3 | NA | 150-500 | | | linear | 0.1 | 0 | Section 3 (Figure S2) |
| Binary $y$ | Factor model | 3 | NA | 200-1000 | | | binary | 0.1 | 0 | Figure S6 |

$$\mathrm{TPP} = \frac{S}{m_1} \tag{9}$$

where $S$ is the number of true positives in the selected variable set and $m_1$ is the total number of true alternative hypotheses (i.e., the known important variables).

As mentioned in Section 2.1, FDR of knockoff filtering with different knockoff generating methods are estimated as the average FDP and power are estimated as the average TPP over the repeated experiments.

A good knockoff variable generating method should control FDR under the nominal level and achieve as high power as possible.

## S3.6 Preeclampsia datasets

### S3.6.1 Data description

A summary of the data used for semi-simulation and the case studies analyses is presented in Table S9.

**Cell-free RNA sequence data in human serum with preeclampsia** The dataset contains circulating cell-free RNA-seq (cfRNA) data previously collected as part of a prospective longitudinal study of 71 pregnant women (19 with preeclampsia, 52 normotensive controls) by Moufarrej et al. (2022). The filtered count matrix was obtained from GSE192902, including 7,160 genes with a level of at least 0.5 count-per-million (CPM) in at least 75% of samples. The details of the study design, the sample collection, the cfRNA library preparation and data quality assessment were previously described in Moufarrej et al. (2022). We only used samples that were sampled before 12 weeks of gestation (sampling time 1) to avoid repeated measurements in the same individual in our analysis, given the assumption of *i.i.d.* in the knockoff filtering.

**Multi-omics study in human with preeclampsia** To test the robustness of knockoff generating methods in different types of data, we use data from a multi-omics longitudinal study for preeclampsia of 36 pregnant women (18 with preeclampsia and 18 normotensive controls) from cohort 1 or 49 women (29 preeclampsia, 20 normotensive controls, microbiome only) from both cohorts (Marić et al., 2022). The datasets include transcriptomic, metabolic, proteomic data from serum, metabolic data from urine and microbiome data from virginal swap which were sampled from the same individual at the same time point, consisting of 37184 genes, 3621, 1305, 8717 and 1255 variables. Same as the cfRNA-seq data, we only use samples from the first cohort and the first time point (generally before 12 weeks of gestation) in our analysis. Variable prefiltering is applied to the transcriptome data and microbiome data. In transcriptomics data, 6394 genes with a level of at least 0.5 count-per-million (CPM) in at least 75% of samples are kept for the following analysis. In microbiome data, only one OTU from each almost identical pair (sample correlation $>0.95$) is kept. Then, we remove OTUs for which the sum of counts is below a set threshold (0.01%) compared to the total sum of all counts, leaving 144 OTUs kept for the following analysis. For the comparison with *limma* in the case study, we also normalised the microbiome count matrix with TMM.

**SomaLogic proteomics for late-onset preeclampsia** To demonstrate the practical application of our method across datasets with a high number of variables, we used proteomic data measured by the SomaLogic platform from a longitudinal study on late-onset preeclampsia involving 166 women (76 diagnosed with preeclampsia and 90 normotensive controls) Erez et al. (2017). The dataset includes measurements for 1,125 proteins. We used only the samples collected at the first time point for each individual. Prior to analysis, protein abundance values were log-transformed.

Table S9: Summary of data used in semi-simulation and case study.

| Data | Samples | Type | Predictors | Reseponse | Used in |
|---|---|---|---|---|---|
| Moufarrej et al. (2022)<br>Circulating cell-free RNA for<br>preeclampsia prediction<br>(GSE192902) | n = 71<br>(16 preeclamptic,<br>55 normotensive) | Trascriptomics:<br>RNA-seq | 7160 genes | Synthetic | Semi-simulation<br>in Section 4;<br>Figure 3 and S11 |
| | | | 200 most variable genes | Disease condition | Case Study<br>in Section 5;<br>Table S2 |
| | | | 81 genes<br>elevated in placenta | Disease condition | Case Study<br>in Section 5;<br>Table 1 |
| | | | | RNF14 gene<br>expression level | Case Study<br>in Section 5;<br>Table S3 |
| Marić et al. (2022)<br>Multiomics for<br>preeclampsia prediction | n = 36<br>(18 preeclamptic,<br>18 normotensive);<br>n = 49<br>(29 preeclamptic,<br>20 normotensive,<br>only microbiome ) | Proteomics | 1303 proteins | Synthetic | Semi-simulation<br>in Section 4;<br>Figure S13 |
| | | Urine Metabolics | 8171 metabolites | | |
| | | Microbiomics 16s rRNA | 144 OTUs | | |
| | | Plasma Transcriptomics:<br>RNA-seq | 74 genes<br>elevated in placenta | Disease condition | Case Study in<br>Section 5;<br>Table 1 and S1 |
| | | Proteomics | 36 proteins released<br>by placenta | | |
| | | Microbiomics 16s rRNA | 144 OTUs | | |
| Erez et al. (2017)<br>SomaLogic proteomics for late-onset preeclampsia | n = 166 | Proteomics | 1225 proteins | Synthetic | Semi-simulation<br>Figure S12, S15 and S17 |

### S3.6.2 Data preprocessing

For transcriptome data (RNA-seq), Trimmed mean of M-values (TMM) and Counts per million (CPM) are used for normalisation to account for differences in library sizes potentially caused by different sequence depth between samples. Imputed and normalised proteome and metabolome data are log-2 transformed (described in Marić et al. 2022). Log-ratio transformation with Centered Log Ratio (CLR) was applied to microbime data. Taxonomic assignment was performed against the Silva v138.1 database using the `R` package dada2 (Callahan et al., 2016).

## S3.7 Semi-simulation: real $X$ with synthetic $y$

Given the validity of knockoff generators only relies on the distribution of $X$ (see the model-X knockoff definition in Section 2.1), we check the robustness of knockoff generators on real biological data by simulating artificial response data using the real covariate data. The true important variable sets in this semi-simulation are still known, so we are able to assess the FDPs and TPPs for knockoff generators.

**Generating linear $y$ from real data** According to the results of simulation experiments where $y$ is linearly dependent on $X$, with the fixed sample sizes of the real datasets, we set up the number of variables ($p$) for semi-simulation experiments with the $n : p$ ratio ranging from 5:5 to 2:5 in order to keep the TPPs above zero. For the cfRNA dataset with a sample size of 71, we run the semi-simulation with $p = 100, 150, 200$; for the multi-omics dataset with a sample size of 36, we run with $p = 40, 80, 120$. For each iteration, a subset of variables is randomly selected as the dataset for testing to mimic the distribution of the real data. Artificial important variables (25 for scRNA data and 15 for multi-omics) are randomly selected and used for generating the artificial response $y$ by linear combination.

**Generating two-group comparison based on real data** To benchmark the performance in FDR control in limma and Wilcoxon test, which are commonly used to detect the variables with different means between two groups, we conducted semi-simulation experiments on the cfRNA data and SomaLogic proteomics data.

For each run, we generated the group assignment variable $y$ from a Bernoulli (0.5). A subset of 25 important predictors was randomly selected from scaled $X$. For observations where $y = 1$ these important predictors from $X$ were added by mean differences $Z$. We considered two different distribution assumptions for $Z$:

- The mean differences are independent for each important predictor: In this case, the shift applied to each important predictor was independently sampled from a normal distribution $Normal(\beta, 1/n)$, where $\beta$ is the predefined mean difference and $\sigma_j$ is the standard deviation of the corresponding variable $X_j$.
- The mean differences are correlated: In this case, the shift was instead drawn from a multivariate normal distribution $MVN(\mu, \Sigma)$, where $\mu$ is the mean vector (with elements set to $\beta$ for important variables and 0 otherwise) and $\Sigma$ is the empirical correlation matrix of $X$. This preserves the correlation structure among predictors, ensuring that with the shift, the covariance structure of $X$ is preserved.

We set $\beta = 3$ in both cases, which means the mean of the important variables in group 1 is 3 units higher than in group 0. The FDP and TPP were calculated over 50 repetitions.

**Semi-simulation experiments** Knockoff filtering with different knockoff-generating methods for the benchmark is then applied with lasso (or logistic lasso) LCD as importance statistics and the more liberal threshold $T$ to control the modified FDR (mFDR) (Candès et al., 2018). Selected variables are then compared with the known true variables to calculate the FDP and TPP. For each real dataset, semi-simulation experiments are run 50 times with the target FDR at 0.05 for cfRNA data or 0.10 for datasets from the multi-omics study.

## S3.8 Case studies

### S3.8.1 Subsets of variables

We further lowered the number of variables before applying knockoff filtering given the observed limitation of near-zero power when the $p : n$ ratio is too large. For the cfRNA data, we selected 200 genes with the highest variance and apply PLSKO and knockoff filtering to identify variables associated with preeclampsia. Semi-simulation is also run to confirm the validity.

Alternatively, we also reduced variable size according to biological knowledge, leading to more specific biological questions. As cfRNA is derived from many tissues in the body, we also select genes that are placental-specific elevated measured in the HPA(v.19; https://v19.proteinatlas.org/humanproteome/tissue/placenta; or the most updated v23, results showed in Supplementary)(Uhlen et al., 2019), aiming to identify physiological changes associated with preeclampsia in the first-trimester placenta. Eighty-one placental-specific elevated genes are found in the prefiltered cfRNA dataset. Similarly, 36 placenta-derived proteins were kept in the proteomics data from the multi-omics study, according to the identification study by Degnes et al. (2022).

### S3.8.2 Application of knockoff filtering

For each dataset, knockoff filtering with the difference of coefficients in the logistics lasso regression as the importance statistics was run 50 times with the target FDR level 0.05. The more liberal threshold $T$ to control the modified FDR (mFDR) (Candès et al., 2018) was used for higher power. Selection frequency of the variables is reported when their selection frequency higher than 0.1. Aggregation of multiple knockoffs (AKO, Nguyen et al. 2020) was then used to integrate the repeatedly generated knockoff variables to improve the stability with $\gamma = 0.3$, namely PLS-AKO.

### S3.8.3 Including additional covariates

In practice, other covariates (e.g., clinical variables) may be included in the variable selection model. These covariates are typically not targets for selection or inference but may help reduce confounding effects or improve power.

Within the knockoff framework, knockoff variables are not needed to generate for the covariates. Instead, we include them directly in the selection model, i.e., we fit the model on $[C, X, \widetilde{X}]$, where $C \in \mathbb{R}^{n \times m}$ is the $m$ covariate variables, instead of $[X, \widetilde{X}]$. In sparse models like lasso, covariates are not regularised and the coefficients of the covariates are estimated as usual.

Although we did not include the covariates in the case studies due to data unavailability, this feature is implemented in our R package. See Example 5 in our vignette: https://guannan-yang.github.io/PLSKO/PLSKO.html.

## S3.9 Another strategy of multiple-knockoff: PLSKO-GZ and comparison to PLS-AKO

Besides AKO (Section S3.3.3), Gimenez and Zou (2019) proposed another strategy to integrate multiple knockoffs, in order to address the instability of the knockoffs, and to improve power. The idea extends the original knockoff framework by: (1) extending *swap* exchangeability in the the model-X knockoff definition to *permute* exchangeability among multiple knockoffs; (2) adapt the filtering threshold from the difference between the original and knockoff statistics to the difference between the maximum of statistics and the second maximum of statistics; and using the indicator whether the original variable has the maximum statistic instead of using the sign of the statistic to estimate the number of false positives. They demonstrated power improvement when the number of non-null variables is small, e.g. less than $1/q$ (, where $q$ is the target FDR).

### S3.9.1 PLSKO-GZ knockoff generator

Following Gimenez and Zou (2019), we extended our PLSKO to generate multiple knockoffs, which we refer to as PLSKO-GZ (See pseudo code in Algorithm S3 below).

Algorithm S3: Sequentially Multi-knockoff Generation with PLSKO (PLSKO-GZ)

**Require:** Centred dataset $X \in \mathbb{R}^{n\times p}$
Neighbour sets $\{BN_j\}_{j=1}^{p}$
Number of PLS components $r$
Number of knockoff copies $\kappa$

**for** $i = 1$ **to** $\kappa$ **do** ▷ sequential over knockoff sets
  **for** $j = 1$ **to** $p$ **do** ▷ sequential over variables
    $BN_{ko,j}^{(1:i)} \leftarrow \{k \in BN_j : k < j, i < \kappa\}$ ▷ knockoff neighbours already available
    Fit (possibly sparse) PLS regression on $X_j$ versus $[\, X_{BN_j},\ \widetilde{X}^{(1:i)}_{BN_{ko,j}} \,]$ using $r$ components
    Obtain fitted values $\hat{X}_j^{(i)}$
    Compute residuals $\hat{\varepsilon}_j^{(i)} = X_j - \hat{X}_j^{(i)}$
    $\tilde{\varepsilon}_j^{(i)} \leftarrow$ random permutation of $\hat{\varepsilon}_j^{(i)}$
    $\widetilde{X}_j^{(i)} \leftarrow \hat{X}_j^{(i)} + \tilde{\varepsilon}_j^{(i)}$
  **end for**
**end for**
**Return** $\widetilde{X}^{(1:\kappa)} = \big(\widetilde{X}^{(1)}, \ldots, \widetilde{X}^{(\kappa)}\big)$

### S3.9.2 Simulations

We conducted simulations on $X$ that simply follow multivariate Gaussian distributions to compare the performance of multiple knockoffs, single knockoffs and PLSAKO, in both low-dimensional (Figure S23) and high-dimensional (Figure S24) settings. Multiple knockoffs generated by multiple SDP (SDP-GZ), multiple MVR (MVR-GZ) were used as the baseline. We generated multiple knockoffs, up to 3, as in the original paper.

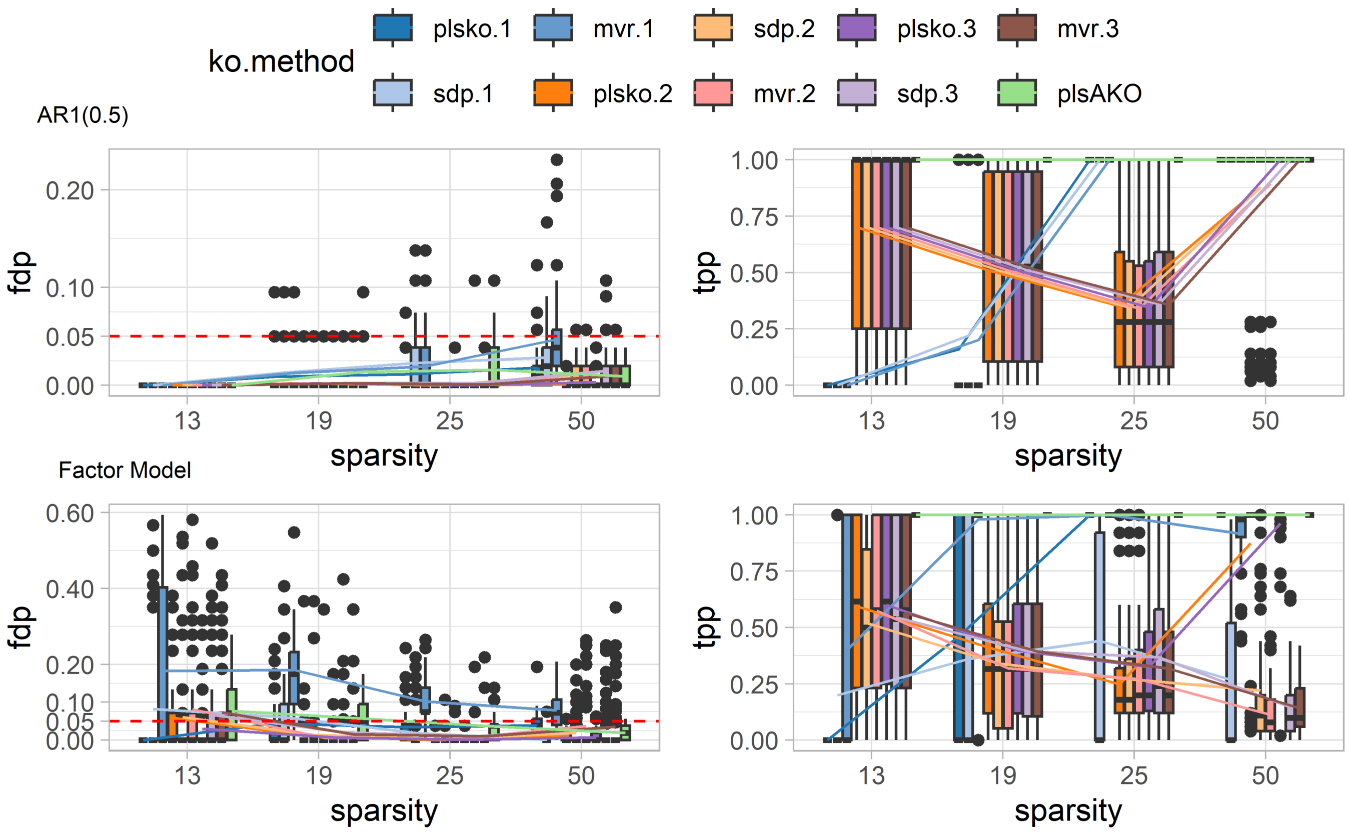


Figure S23: Simulation results of PLSKO-GZ, SDP-GZ, MVR-GZ, as well as PLS-AKO on the block multivariate Gaussian with AR(1), $\rho = 0.5$ (upper panel) and factor model (lower panel), sample size $n = 250$ and number of variables $p = 250$, w.r.t. different signal variable numbers used to generate the linear response variable $y$, corresponding to $5\%, 7.5\%, 10\% and 20\%$. The results are averaged over 50 replications. Target FDR is set to 0.05. plsko.1: single PLSKO, plsko.2: 2 multiple-knockoffs generated by PLSKO-GZ, plsko.3: 3 multiple-knockoffs generated by PLSKO-GZ. fdp: false discovery proportion, tpp: true positive proportion.

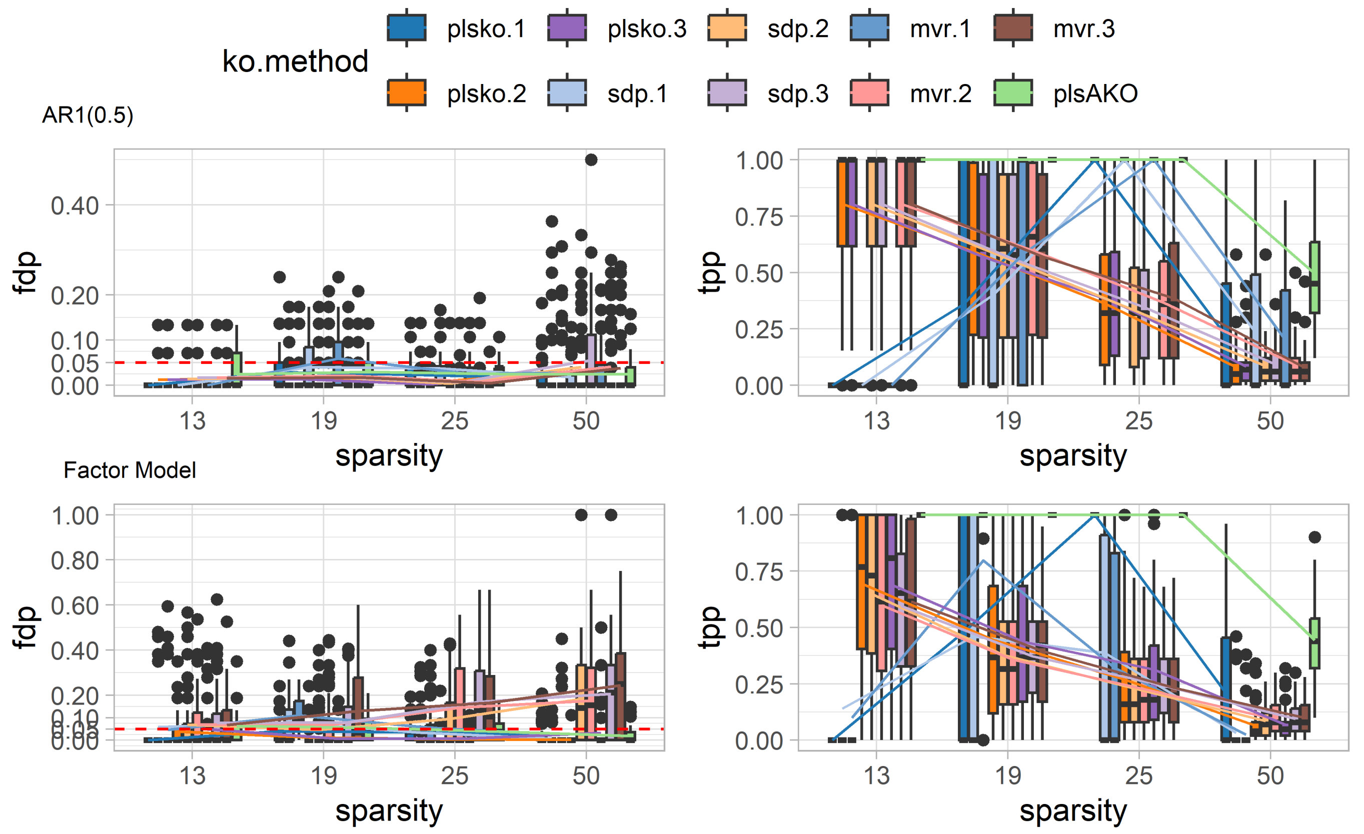


Figure S24: Simulation results of multiple knockoffs. Simulation settings are as in Figure S23, but with sample size $n = 150$.

We found that the results of different knockoff generators PLSKO-GZ, SDP-GZ and MVR-GZ were generally similar in terms of power and FDR control for the same number of knockoffs. FDR was controlled in most cases, except for SDP and MVR multiple knockoffs where FDR was inflated in the high-dimensional factor model when the number of non-null variables increased.

In terms of power, we found similar results to Figure 2 in the original paper, i.e., multiple knockoffs can improve power when the number of non-null variables is small: all single knockoffs had no power when the number of non-null variables is 13, whereas the multiple knockoffs had powers at least above 0.5. However, as the number of non-null variables increases, the power of multiple knockoffs started to decrease, and the single knockoffs started to have power. The decrease trend was also found in the original multi-knockoffs paper, although they did not show the results when the number of non-null variables is larger. Power decreases when the non-null proportion increases was observed in our previous single knockoff simulation (Figure S4). We speculate that this effect in multiple knockoffs might happen earlier than the single knockoffs due to their conservative nature.

We found that the PLS-AKO has the best power over the single knockoffs and multiple knockoffs in all scenario, regardless of the number of non-null variables.

**Application on real data** We applied PLSKO-GZ (3 knockoffs) to the same cfRNA data from our case studies. We did not conducted analysis with multiple-knockoffs more than three, since the power would be likely to decrease further (discussed in the original paper). We repeated the PLSKO-GZ 25 times, and compared with single PLSKO. Selection frequencies are shown in Supplemental Table S10. PLSKO-GZ generally selected fewer variables, and with lower frequency than the single PLSKO. We also applied AKO to integrate the importance statistics from the 25 generated PLSKO-GZ.

Table S10: Selection frequency of pre-eclampsia-related genes from PLSKO-GZ (3 knockoffs) and PLSKO (single knockoffs). AKO selection indicated in bold text.

| **Gene name** | **Selection Freq: 3 Knockoffs** | **Selection Freq: 1 Knockoffs** |
|---|---|---|
| FAM46A | **0.84** | **1** |
| PHACTR2 | **0.6** | **0.8** |
| MBNL3 | **0.56** | **0.84** |
| MAFK | **0.32** | **0.6** |
| GSE1 | **0.24** | **0.56** |
| BPGM | **0.24** | **0.48** |
| CSF2RB | 0.08 | **0.32** |
| HEMGN | **0.08** | **0.36** |
| TRIM10 | 0.04 | **0.2** |
| COBLL1 | 0 | **0.2** |
| AFF1 | 0 | **0.16** |

**Conclusion** In summary, we found that PLSKO-GZ can generate multiple knockoffs, but the results are similar to the single PLSKO. The multiple knockoffs can improve power when the number of non-null variables is smaller than $1/q$, but the power decreases as the number of non-null variables increases. PLS-AKO has the best power over all scenarios. Multiple knockoffs did not improve the stability of variable selection in our case studies, therefore we recommend using single PLSKO or PLS-AKO instead of PLSKO-GZ. We provide our R code to assist readers reproducing the results and investigating multiple knockoffs: https://github.com/guannan-yang/PLSKO/tree/main/paper%20codes.

## S3.10 Software packages used

All the experiments were run in `R` (v 4.3.0) and `Julia` (v 1.9.3), with following listed packages:

- **mixOmics** `R` package (Rohart et al., 2017) (v 6.25.1): used for PLS regression and sparse PLS regression.
- **knockoff** `R` package (Patterson and Sesia, 2022) (v 0.3.6): used for the second-order approximation knockoff construction with SDP and generate LCD.
- **glmnet** `R` (Friedman et al., 2010; Tay et al., 2023) package (v 4.1-8) : used to generate LCD.
- **RSpectra** `R` package (v 0.16-1): used for IPAD knockoff and PCKO.
- **JuliaCall** `R` package (v 0.17.5): used to call Julia package in R environment.
- **Knockoffs** `Julia` package (Chu et al., 2024) (v 1.1.5): used for the second-order approximation knockoff construction with MVR and ME.
- **Grace-AKO** `R` package (Tian et al., 2022): used for knockoff aggregation (AKO).
- **edgeR** `R` package (v 3.42.4) (Robinson et al., 2010): used for real data preprocess.
- **limma** `R` package (v 3.56.2) (Smyth, 2005): used for case studies.
- **doParallel** `R` package (v 1.0.17): used for parallel computing in simulation and generating multiple knockoffs in case studies.
- **tidyverse** `R` package (v 2.0.0).
- **Dada2** `R` package (v 1.28.0) Callahan et al. (2016): used for microbiome data taxonomic assignment.
- **cheapknockoff** `R` functions (Yu et al., 2022): used for multiple knockoffs implementation.

# Bibliography

Rina Foygel Barber and Emmanuel J. Candès. Controlling the false discovery rate via knockoffs. The Annals of Statistics, 43(5):2055–2085, 2015. ISSN 0090-5364, 2168-8966. doi:10.1214/15-AOS1337. URL https://projecteuclid.org/journals/annals-of-statistics/volume-43/issue-5/Controlling-the-false-discovery-rate-via-knockoffs/10.1214/15-AOS1337.full.

Anne-Laure Boulesteix and Korbinian Strimmer. Partial least squares: A versatile tool for the analysis of high-dimensional genomic data. Briefings in Bioinformatics, 8(1):32–44, January 2007. ISSN 1467-5463. doi:10.1093/bib/bbl016.

Benjamin J Callahan, Paul J McMurdie, Michael J Rosen, Andrew W Han, Amy Jo A Johnson, and Susan P Holmes. Dada2: High resolution sample inference from illumina amplicon data. Nature methods, 13(7):581–583, July 2016. ISSN 1548-7091. doi:10.1038/nmeth.3869. URL https://www.ncbi.nlm.nih.gov/pmc/articles/PMC4927377/.

Emmanuel Candès, Yingying Fan, Lucas Janson, and Jinchi Lv. Panning for gold: 'model-X' knockoffs for high dimensional controlled variable selection. Journal of the Royal Statistical Society: Series B (Statistical Methodology), 80(3):551–577, 2018. ISSN 1467-9868. doi:10.1111/rssb.12265. URL https://onlinelibrary.wiley.com/doi/abs/10.1111/rssb.12265.

Benjamin B. Chu, Jiaqi Gu, Zhaomeng Chen, Tim Morrison, Emmanuel Candes, Zihuai He, and Chiara Sabatti. Second-order group knockoffs with applications to gwas. (arXiv:2310.15069), March 2024. doi:10.48550/arXiv.2310.15069. URL http://arxiv.org/abs/2310.15069. arXiv:2310.15069 [q-bio, stat].

Sijmen De Jong. Simpls: an alternative approach to partial least squares regression. Chemometrics and intelligent laboratory systems, 18(3):251–263, 1993.

Maren-Helene Langeland Degnes, Ane Cecilie Westerberg, Manuela Zucknick, Theresa L. Powell, Thomas Jansson, Tore Henriksen, Marie Cecilie Paasche Roland, and Trond Melbye Michelsen. Placenta-derived proteins across gestation in healthy pregnancies—a novel approach to assess placental function? BMC Medicine, 20(1):227, July 2022. ISSN 1741-7015. doi:10.1186/s12916-022-02415-z. URL https://doi.org/10.1186/s12916-022-02415-z.

Offer Erez, Roberto Romero, Eli Maymon, Piya Chaemsaithong, Bogdan Done, Percy Pacora, Bogdan Panaitescu, Tinnakorn Chaiworapongsa, Sonia S. Hassan, and Adi L. Tarca. The prediction of late-onset preeclampsia: Results from a longitudinal proteomics study. PLOS ONE, 12(7):e0181468, July 2017. ISSN 1932-6203. doi:10.1371/journal.pone.0181468. URL https://journals.plos.org/plosone/article?id=10.1371/journal.pone.0181468.

Yingying Fan, Jinchi Lv, Mahrad Sharifvaghefi, and Yoshimasa Uematsu. IPAD: Stable Interpretable Forecasting with Knockoffs Inference. Journal of the American Statistical Association, 115(532):1822–1834, 2020. ISSN 0162-1459. doi:10.1080/01621459.2019.1654878. URL https://doi.org/10.1080/01621459.2019.1654878.

Jerome Friedman, Trevor Hastie, and Rob Tibshirani. Regularization paths for generalized linear models via coordinate descent. Journal of Statistical Software, 33(1):1–22, 2010. ISSN 1548-7660.

Jaime Roquero Gimenez and James Zou. Improving the Stability of the Knockoff Procedure: Multiple Simultaneous Knockoffs and Entropy Maximization. In Proceedings of the Twenty-Second International Conference on Artificial Intelligence and Statistics, pages 2184–2192. PMLR, 2019. URL https://proceedings.mlr.press/v89/gimenez19b.html.

Jaime Roquero Gimenez, Amirata Ghorbani, and James Zou. Knockoffs for the mass: New feature importance statistics with false discovery guarantees, 2019. URL http://arxiv.org/abs/1807.06214.

Zihuai He, Linxi Liu, Chen Wang, Yann Le Guen, Justin Lee, Stephanie Gogarten, Fred Lu, Stephen Montgomery, Hua Tang, Edwin K. Silverman, Michael H. Cho, Michael Greicius, and Iuliana Ionita-Laza. Identification of putative causal loci in whole-genome sequencing data via knockoff statistics. Nature Communications, 12(1):3152, May 2021. ISSN 2041-1723. doi:10.1038/s41467-021-22889-4.

Agnar Höskuldsson. PLS regression methods. J. Chemom., 2(3):211–228, June 1988.

Tao Jiang, Yuanyuan Li, and Alison A Motsinger-Reif. Knockoff boosted tree for model-free variable selection. Bioinformatics, 37(7):976–983, April 2021. ISSN 1367-4803. doi:10.1093/bioinformatics/btaa770. URL https://doi.org/10.1093/bioinformatics/btaa770.

Matthias Kormaksson, Luke J. Kelly, Xuan Zhu, Sibylle Haemmerle, Luminita Pricop, and David Ohlssen. Sequential knockoffs for continuous and categorical predictors: With application to a large psoriatic arthritis clinical trial pool. Statistics in Medicine, 40(14):3313–3328, 2021. ISSN 1097-0258. doi:10.1002/sim.8955. URL https://onlinelibrary.wiley.com/doi/abs/10.1002/sim.8955.

Olivier Ledoit and Michael Wolf. Improved estimation of the covariance matrix of stock returns with an application to portfolio selection. Journal of Empirical Finance, 10(5):603–621, December 2003. ISSN 0927-5398. doi:10.1016/S0927-5398(03)00007-0.

Ivana Marić, Kévin Contrepois, Mira N. Moufarrej, Ina A. Stelzer, Dorien Feyaerts, Xiaoyuan Han, Andy Tang, Natalie Stanley, Ronald J. Wong, Gavin M. Traber, Mathew Ellenberger, Alan L. Chang, Ramin Fallahzadeh, Huda Nassar, Martin Becker, Maria Xenochristou, Camilo Espinosa, Davide De Francesco, Mohammad S. Ghaemi, Elizabeth K. Costello, Anthony Culos, Xuefeng B. Ling, Karl G. Sylvester, Gary L. Darmstadt, Virginia D. Winn, Gary M. Shaw, David A. Relman, Stephen R. Quake, Martin S. Angst, Michael P. Snyder, David K. Stevenson, Brice Gaudilliere, and Nima Aghaeepour. Early prediction and longitudinal modeling of preeclampsia from multiomics. Patterns, 3(12), December 2022. ISSN 2666-3899. doi:10.1016/j.patter.2022.100655.

Mira N. Moufarrej, Sevahn K. Vorperian, Ronald J. Wong, Ana A. Campos, Cecele C. Quaintance, Rene V. Sit, Michelle Tan, Angela M. Detweiler, Honey Mekonen, Norma F. Neff, Courtney Baruch-Gravett, James A. Litch, Maurice L. Druzin, Virginia D. Winn, Gary M. Shaw, David K. Stevenson, and Stephen R. Quake. Early prediction of preeclampsia in pregnancy with cell-free rna. Nature, 602(7898):689–694, February 2022. ISSN 1476-4687. doi:10.1038/s41586-022-04410-z.

Tuan-Binh Nguyen, Jerome-Alexis Chevalier, Bertrand Thirion, and Sylvain Arlot. Aggregation of Multiple Knockoffs. In Proceedings of the 37th International Conference on Machine Learning, pages 7283–7293. PMLR, November 2020.

Evan Patterson and Matteo Sesia. knockoff: The Knockoff Filter for Controlled Variable Selection, 2022. URL https://CRAN.R-project.org/package=knockoff. R package version 0.3.6.

Mark D. Robinson, Davis J. McCarthy, and Gordon K. Smyth. edger: a bioconductor package for differential expression analysis of digital gene expression data. Bioinformatics, 26(1):139–140, January 2010. ISSN 1367-4803. doi:10.1093/bioinformatics/btp616. URL https://doi.org/10.1093/bioinformatics/btp616.

Florian Rohart, Benoît Gautier, Amrit Singh, and Kim-Anh Lê Cao. mixOmics: An R package for ’omics feature selection and multiple data integration. PLoS computational biology, 13(11):e1005752, November 2017. ISSN 1553-7358. doi:10.1371/journal.pcbi.1005752.

Yaniv Romano, Matteo Sesia, and Emmanuel Candès. Deep Knockoffs. Journal of the American Statistical Association, 115(532):1861–1872, 2020. ISSN 0162-1459. doi:10.1080/01621459.2019.1660174. URL https://doi.org/10.1080/01621459.2019.1660174.

Roman Rosipal and Nicole Krämer. Overview and Recent Advances in Partial Least Squares. In Craig Saunders, Marko Grobelnik, Steve Gunn, and John Shawe-Taylor, editors, Subspace, Latent Structure and Feature Selection, Lecture Notes in Computer Science, pages 34–51, Berlin, Heidelberg, 2006. Springer. ISBN 978-3-540-34138-3. doi:10.1007/11752790_2.

M Sesia, C Sabatti, and E J Candès. Gene hunting with hidden Markov model knockoffs. Biometrika, 106(1):1–18, 2019. ISSN 0006-3444. doi:10.1093/biomet/asy033. URL https://doi.org/10.1093/biomet/asy033.

G. K. Smyth. Limma: Linear Models for Microarray Data. In Robert Gentleman, Vincent J. Carey, Wolfgang Huber, Rafael A. Irizarry, and Sandrine Dudoit, editors, Bioinformatics and Computational Biology Solutions Using R and Bioconductor, pages 397–420. Springer-Verlag, New York, 2005. ISBN 978-0-387-25146-2. doi:10.1007/0-387-29362-0_23.

Asher Spector and Lucas Janson. Powerful knockoffs via minimizing reconstructability. The Annals of Statistics, 50(1):252–276, February 2022. ISSN 0090-5364, 2168-8966. doi:10.1214/21-AOS2104. URL https://projecteuclid.org/journals/annals-of-statistics/volume-50/issue-1/Powerful-knockoffs-via-minimizing-reconstructability/10.1214/21-AOS2104.full.

J. Kenneth Tay, Balasubramanian Narasimhan, and Trevor Hastie. Elastic net regularization paths for all generalized linear models. Journal of Statistical Software, 106:1–31, March 2023. ISSN 1548-7660. doi:10.18637/jss.v106.i01. URL https://doi.org/10.18637/jss.v106.i01.

Peixin Tian, Yiqian Hu, Zhonghua Liu, and Yan Dora Zhang. Grace-AKO: A novel and stable knockoff filter for variable selection incorporating gene network structures. BMC Bioinformatics, 23(1):478, November 2022. ISSN 1471-2105. doi:10.1186/s12859-022-05016-y.

Mathias Uhlen, Max J. Karlsson, Wen Zhong, Abdellah Tebani, Christian Pou, Jaromir Mikes, Tadepally Lakshmikanth, Björn Forsström, Fredrik Edfors, Jacob Odeberg, Adil Mardinoglu, Cheng Zhang, Kalle von Feilitzen, Jan Mulder, Evelina Sjöstedt, Andreas Hober, Per Oksvold, Martin Zwahlen, Fredrik Ponten, Cecilia Lindskog, Åsa Sivertsson, Linn Fagerberg, and Petter Brodin. A genome-wide transcriptomic analysis of protein-coding genes in human blood cells. Science (New York, N.Y.), 366(6472):eaax9198, December 2019. ISSN 1095-9203. doi:10.1126/science.aax9198.

Herman Wold. Estimation of principal components and related models by iterative least squares. Multivariate Analysis, pages 391–420, 1966.

Herman Wold. Path models with latent variables: The NIPALS approach., 1975.

Guo Yu, Daniela Witten, and Jacob Bien. Controlling costs: Feature selection on a budget. Stat, 11(1):e427, 2022. ISSN 2049-1573. doi:10.1002/sta4.427. URL https://onlinelibrary.wiley.com/doi/abs/10.1002/sta4.427.
